# DNA methylation landscapes in DIPG reveal methylome variability that can be modified pharmacologically

**DOI:** 10.1093/noajnl/vdae023

**Published:** 2024-02-19

**Authors:** Ashley R Tetens, Allison M Martin, Antje Arnold, Orlandi V Novak, Adrian Idrizi, Rakel Tryggvadottir, Jordyn Craig-Schwartz, Athanasia Liapodimitri, Kayleigh Lunsford, Michael I Barbato, Charles G Eberhart, Adam C Resnick, Eric H Raabe, Michael A Koldobskiy

**Affiliations:** Center for Epigenetics, Johns Hopkins University School of Medicine, Baltimore, Maryland, USA; Pediatric Oncology, Sidney Kimmel Comprehensive Cancer Center, Johns Hopkins University School of Medicine, Baltimore, Maryland, USA; Pediatric Hematology-Oncology, Albert Einstein College of Medicine, Bronx, New York, USA; Pediatric Oncology, Sidney Kimmel Comprehensive Cancer Center, Johns Hopkins University School of Medicine, Baltimore, Maryland, USA; Pediatric Oncology, Sidney Kimmel Comprehensive Cancer Center, Johns Hopkins University School of Medicine, Baltimore, Maryland, USA; Center for Epigenetics, Johns Hopkins University School of Medicine, Baltimore, Maryland, USA; Center for Epigenetics, Johns Hopkins University School of Medicine, Baltimore, Maryland, USA; Center for Epigenetics, Johns Hopkins University School of Medicine, Baltimore, Maryland, USA; Pediatric Oncology, Sidney Kimmel Comprehensive Cancer Center, Johns Hopkins University School of Medicine, Baltimore, Maryland, USA; Center for Epigenetics, Johns Hopkins University School of Medicine, Baltimore, Maryland, USA; Pediatric Oncology, Sidney Kimmel Comprehensive Cancer Center, Johns Hopkins University School of Medicine, Baltimore, Maryland, USA; Center for Epigenetics, Johns Hopkins University School of Medicine, Baltimore, Maryland, USA; Pediatric Oncology, Sidney Kimmel Comprehensive Cancer Center, Johns Hopkins University School of Medicine, Baltimore, Maryland, USA; Center for Epigenetics, Johns Hopkins University School of Medicine, Baltimore, Maryland, USA; Pediatric Oncology, Sidney Kimmel Comprehensive Cancer Center, Johns Hopkins University School of Medicine, Baltimore, Maryland, USA; Neuropathology, Johns Hopkins University School of Medicine, Baltimore, Maryland, USA; Center for Data-Driven Discovery in Biomedicine, The Children’s Hospital of Philadelphia, Philadelphia, Pennsylvania, USA; Division of Neurosurgery, The Children’s Hospital of Philadelphia, Philadelphia, Pennsylvania, USA; Pediatric Oncology, Sidney Kimmel Comprehensive Cancer Center, Johns Hopkins University School of Medicine, Baltimore, Maryland, USA; Neuropathology, Johns Hopkins University School of Medicine, Baltimore, Maryland, USA; Center for Epigenetics, Johns Hopkins University School of Medicine, Baltimore, Maryland, USA; Pediatric Oncology, Sidney Kimmel Comprehensive Cancer Center, Johns Hopkins University School of Medicine, Baltimore, Maryland, USA

**Keywords:** diffuse intrinsic pontine glioma, diffuse midline glioma, DNA hypomethylating agents, epigenetic variability

## Abstract

**Background:**

Diffuse intrinsic pontine glioma (DIPG) is a uniformly lethal brainstem tumor of childhood, driven by histone H3 K27M mutation and resultant epigenetic dysregulation. Epigenomic analyses of DIPG have shown global loss of repressive chromatin marks accompanied by DNA hypomethylation. However, studies providing a static view of the epigenome do not adequately capture the regulatory underpinnings of DIPG cellular heterogeneity and plasticity.

**Methods:**

To address this, we performed whole-genome bisulfite sequencing on a large panel of primary DIPG specimens and applied a novel framework for analysis of DNA methylation variability, permitting the derivation of comprehensive genome-wide DNA methylation potential energy landscapes that capture intrinsic epigenetic variation.

**Results:**

We show that DIPG has a markedly disordered epigenome with increasingly stochastic DNA methylation at genes regulating pluripotency and developmental identity, potentially enabling cells to sample diverse transcriptional programs and differentiation states. The DIPG epigenetic landscape was responsive to treatment with the hypomethylating agent decitabine, which produced genome-wide demethylation and reduced the stochasticity of DNA methylation at active enhancers and bivalent promoters. Decitabine treatment elicited changes in gene expression, including upregulation of immune signaling such as the interferon response, STING, and MHC class I expression, and sensitized cells to the effects of histone deacetylase inhibition.

**Conclusions:**

This study provides a resource for understanding the epigenetic instability that underlies DIPG heterogeneity. It suggests the application of epigenetic therapies to constrain the range of epigenetic states available to DIPG cells, as well as the use of decitabine in priming for immune-based therapies.

**Key Points**

- We generate comprehensive maps of the dynamic DNA methylation landscape in DIPG that captures epigenetic variation.
- DIPG has a markedly disordered epigenome, with increasingly stochastic (random) DNA methylation localizing to key regulatory elements and genes.
- The DIPG methylation landscape can be modified with hypomethylating drugs to constrain plasticity, alter gene expression, and induce immune signaling.

**Importance of Study**

DIPG is understood to be an epigenetically driven disease, but conventional analyses that provide a static look at the epigenome do not capture the epigenetic variability that underlies intratumoral heterogeneity. We have generated a large whole-genome bisulfite sequencing dataset (23 primary specimens, 4 patient-derived neurosphere cell lines, and 4 normal controls) and applied novel analytical tools that generate comprehensive genome-wide maps of epigenetic variability. We show that DIPG has a markedly destabilized epigenome, with increasingly stochastic DNA methylation at genes regulating pluripotency and developmental identity, suggesting a mechanism for cellular heterogeneity and plasticity. We show that the DIPG epigenetic landscape can be modified with hypomethylating drugs to constrain plasticity, alter gene expression, and induce immune activation and expression of novel antigens. This study provides a resource for dissecting the epigenetic instability that underlies DIPG heterogeneity, and suggests a strategy to pharmacologically constrain epigenetic plasticity in DIPG.

Diffuse intrinsic pontine glioma (DIPG), also known as diffuse midline glioma (DMG), is an incurable childhood brainstem tumor with median survival of <1 year. Chemotherapy is ineffective and palliative radiotherapy remains the only standard-of-care treatment, creating an urgent need for novel therapies.^1,2^ A breakthrough in the understanding of DIPG biology was the discovery of driver mutations in histone H3 in most cases.^3,4^ The histone H3 K27M mutation produces a dominant-negative effect on gene-repressive H3K27 trimethylation by EZH2, a component of the Polycomb Repressive Complex 2 (PRC2). This leads to defective chromatin spread of the repressive H3K27me3 mark, thereby resulting in a global loss in H3K27me3, accompanied by DNA hypomethylation and aberrant gene expression.^5–9^ Although these findings point to a critical role for epigenetic dysregulation in DIPG, the link between epigenetic alterations and tumorigenesis remains unclear, creating a critical barrier to progress. Additionally, studies investigating directional changes in epigenetic marks across bulk cell populations do not fully capture the heterogeneity and variability intrinsic to cancer.^10^

Epigenetic variability is a major driving force in tumor evolution.^11–14^ The emergence of epigenetic stochasticity, or non-deterministic changes in epigenetic marks giving rise to epigenetic variation, underlies the phenotypic plasticity of cancer cells and allows for selection of cellular traits that promote survival in a changing environment, allowing cells to evade therapies.^13^ Studies providing a static view of the DIPG epigenome may fail to capture the diversity and plasticity of DIPG epigenetic states. This is supported by single-cell RNA-seq studies in DIPG that reveal marked intratumoral heterogeneity, with subpopulations of malignant cells encompassing a range of transcriptional programs reflecting distinct differentiation states.^15^ Mapping the dynamic epigenetic landscape in a manner that captures intrinsic epigenetic variation is necessary to understand the underpinnings of phenotypic plasticity in DIPG.

Conventional methods for DNA methylation analysis are limited in several ways. Methods utilizing array-based DNA methylation analysis evaluate only a small fraction of CpG sites in the genome and measure only differences in mean methylation levels.^16^ Next-generation sequencing-based DNA methylation analyses can assess many more CpG sites, with whole-genome bisulfite sequencing (WGBS) serving as the “gold standard,” capable of assessing essentially all genomic CpGs. WGBS affords multiple advantages, including single-base resolution, quantitative nature, lack of bias for CpG-rich promoter regions, and ability to capture variability of methylation patterns. However, existing methods of WGBS analysis largely rely on smoothing/averaging methylation data and comparing differences in mean levels, dismissing much of the statistical information contained within WGBS reads. Feinberg and Goutsias recently developed a novel approach employing principles from statistical physics and information theory to derive DNA methylation potential energy landscapes from WGBS data, enabling quantification of methylation variability genome-wide using Shannon’s entropy.^17^ We have subsequently carried out a proof-of-principle application of this method to identify non-mutated, epigenetically altered driver genes in childhood leukemia, and have developed tools for comparative analysis of epigenetic potential energy landscapes to rank genes and genomic regions by epigenetic discordance.^18,19^

Here, we sought to characterize the dynamic DNA methylation landscape of DIPG in a manner that captures epigenetic stochasticity, by carrying out WGBS coupled with DNA methylation potential energy landscape analysis.^17,20^ This permits evaluation of mean methylation levels, methylation stochasticity (quantified as methylation entropy), and identification of differentially methylated regions based on discordance in DNA methylation probability distributions. We generated a large WGBS dataset of 23 primary DIPG patient samples, 4 normal fetal brain controls, and 4 patient-derived neurosphere DIPG cell lines to generate comprehensive maps of the dynamic DNA methylation landscape of DIPG. This revealed a markedly destabilized epigenome in DIPG, with increasingly stochastic DNA methylation at key regulatory regions and genes. We investigated whether the DNA methylation landscape could be modified with the hypomethylating drug decitabine (DAC), showing that this can constrain plasticity, alter gene expression, and induce immune signaling in DIPG cells.

## Materials and Methods

### Ethics Statement

Human subjects were de-identified prior to analysis, and this research does not constitute human subjects research under DHHS/FDA regulations.

### Sample Preparation and Nucleic Acid Extraction

Primary patient samples were obtained from the Children’s Brain Tumor Network (CBTN). Sample characteristics are provided in Supplementary Table 1. Methods for nucleic acid isolation and quality control are provided in the Supplementary Materials.

### WGBS Library Preparation and Sequencing

WGBS library preparation and sequencing were carried out as previously described.^21^ Detailed methods are provided in the Supplementary Materials. Coverage is indicated in Supplementary Table 1.

### RNA Sequencing

Strand specific mRNA libraries were generated using the NEBNext Ultra II Directional RNA library prep Kit for Illumina (New England BioLabs #E7760), mRNA was isolated using Poly(A) mRNA magnetic isolation module (New England BioLabs #E7490). Preparation of libraries followed the manufacturer’s protocol (Version 1.0 4/17). Detailed methods are provided in the Supplementary Materials.

### Genomic Features and Annotations

Genomic features and annotations were defined as previously described.^21^ Files and tracks utilize genomic coordinates for hg19. CGIs were obtained from Wu et al.^22^ Chromatin functional annotations were obtained using the ChromHMM 25-state reference model.^23^ ChromHMM 25-state enhancer and promoter annotations with definitions and emission parameters as previously described were employed.^24^ Detailed methods are provided in the Supplementary Materials.

### WGBS Analysis and PEL Computation

We computed DNA methylation potential energy landscapes (PELs) from WGBS data using informME (v0.3.3), an information theoretic pipeline for methylation analysis based on the one-dimensional Ising model of statistical physics, with methods previously described.^21^ We performed differential analysis between test (DIPG) and reference (normal fetal brain) WGBS samples using informME.^19^ Within a given region of analysis, we computed Jensen-Shannon distances (JSDs) between the corresponding methylation level probability distributions, as well as differences between mean methylation levels (dMMLs) and normalized methylation entropies (dNMEs). Gene and genomic feature ranking by methylation discordance was achieved using the “jsGrank” utility of informME, which uses the JSD to identify regions of the genome with statistically significant discordance in methylation stochasticity. Detailed methods are provided in the Supplementary Materials.

### Cell Culture and Drug Treatments

Cell lines and culture conditions are described in the Supplementary Methods. Decitabine (DAC) and vorinostat (SAHA) were purchased from Cayman Chemical (#11166, #10009929). Cells were treated with 100 nm DAC or DMSO for 5 days, with media and drug change every 24 h. For the RNA-Seq and WGBS experiments, the DMSO and DAC-treated cells were harvested at the 72-h time point (day 8). For flow cytometry, apoptosis, and proliferation assays, 300 nm SAHA was added on the fifth day to one of the DMSO-treated conditions and one of the 5-day DAC-treated conditions following drug and media change.

### Flow Cytometry, Western Blot, BrdU Incorporation, and Cleaved Caspase 3 Detection

Detailed methods for flow cytometry and Western blotting, including all antibodies used, are provided in the Supplementary Materials. Bromodeoxyuridine (BrdU) assays and cleaved caspase 3 immunofluorescence detection were performed as previously described.^25,26^

## Results

### Potential Energy Landscapes Encapsulate DNA Methylation Stochasticity in DIPG.

**Figure 1.**
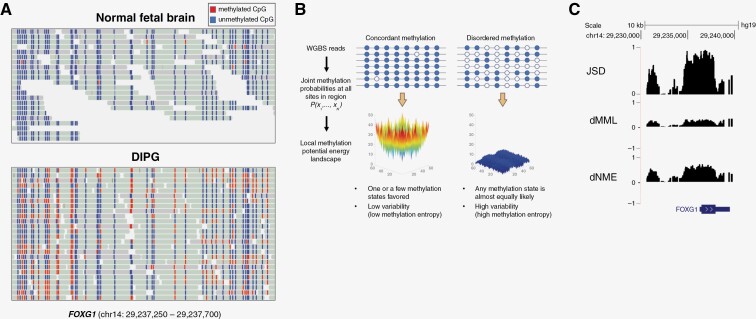
DNA methylation potential energy landscapes encapsulate methylation stochasticity in DIPG. (a) WGBS reads within the promoter of FOXG1 from normal fetal brain and a representative H3 K27M glioma. Red indicates a methylated CpG, while blue indicates an unmethylated CpG. In this region, DIPG exhibits mean hypermethylation, but also a marked increase in DNA methylation as compared to normal fetal brain. (b) Schematic summarizing the informME analytic pipeline. WGBS reads are used to model the joint probability distribution of methylation using the Ising model of statistical physics, permitting evaluation of DNA methylation stochasticity as quantified by methylation entropy. (c) Genome browser view of the *FOXG1* promoter showing near-maximal discordance between DNA methylation probability distributions, as quantified by the Jensen-Shannon distance (JSD). Mean hypermethylation of the region in DIPG vs normal is shown by the differential mean methylation level (dMML). A marked increase in DNA methylation entropy is quantified by the differential normalized methylation entropy (dNME), showing an increase in DIPG as compared to normal.

WGBS reads contain DNA methylation patterning information that may be used to evaluate DNA methylation stochasticity. Initial studies of DNA methylation in H3 K27M glioma employed methylation arrays and identified a distinct methylation signature, including specific DMRs such as higher levels of methylation at the *FOXG1* promoter associated with lower expression in K27M tumors.^27^ A small panel of primary glioma samples was subsequently assessed by WGBS, confirming a trend of genome-wide DNA hypomethylation in K27M glioma, and confirming that *FOXG1* was among the most differentially down-regulated genes in histone H3 K27M as compared to H3 WT pediatric high-grade glioma.^8^ By inspecting the raw WGBS reads in a region of the *FOXG1* gene (Figure 1a) in a representative histone H3 K27M glioma sample from this study (DKFZ-11-005) as compared to normal fetal brain,^28^ it is evident that there is not only a gain in mean DNA methylation in this region in K27M glioma, but also a dramatic increase in the variability or stochasticity of methylated (red) or unmethylated (blue) CpG sites in this region in glioma cells as compared to normal brain. Whereas in a normal brain most reads are fully unmethylated and concordant with one another, in the K27M glioma sample the likelihood that a given CpG is methylated or unmethylated varies greatly between reads (Figure 1a). Feinberg et al. recently described a powerful approach to quantify DNA methylation stochasticity by assessing the joint probability distribution of DNA methylation levels across WGBS reads using the Ising model of statistical physics (Figure 1b).^18,20^ Patterning information in WGBS data in a genomic region is used to compute a potential energy landscape for DNA methylation, wherein each methylation pattern is assigned a potential value, with smaller values indicating that the methylation pattern has a higher probability of being observed. The presence of a deep, narrow “potential well” in the DNA methylation potential energy landscape indicates low methylation stochasticity, such that only a small number of methylation patterns are observed with high probability. In contrast, a wide and flat (shallow) potential energy landscape implies high methylation stochasticity, with many methylation patterns that may be observed with nearly equal probability (Figure 1b). The probability distribution of methylation levels is used to calculate the mean methylation level (MML) within a region, and the stochasticity of methylation levels is summarized by a version of Shannon’s entropy, termed normalized methylation entropy (NME).^18,20^ The Jensen–Shannon distance (JSD) is used to measure differences between two probability distributions of DNA methylation levels between a test (DIPG) and a reference (normal) sample within each analysis region, whether driven by differences in mean methylation, entropy, or other factors. The JSD ranges from 0 to 1 and takes its maximum value when the two probability distributions are maximally discordant. In the region of *FOXG1* shown (Figure 1c), a “peak” of JSD is observed approaching a value of 1, indicating a near-maximal discordance between DNA methylation probability distributions, driven by a small increase in mean methylation in DIPG as compared to normal (shown by the positive deflection in differential MML, dMML), and a large increase in normalized methylation entropy in DIPG as compared to normal (shown by a positive deflection in differential NME, dNME). Given the power of WGBS and DNA methylation potential energy landscape analysis to map the dynamic epigenetic landscape in DIPG in a manner that encapsulates epigenetic heterogeneity/variability, we sought to apply these methods in a large panel of primary DIPG samples.

### WGBS Analysis Reveals Global Hypomethylation and Increased Epigenetic Stochasticity in DIPG Primary Samples

**Figure 2.**
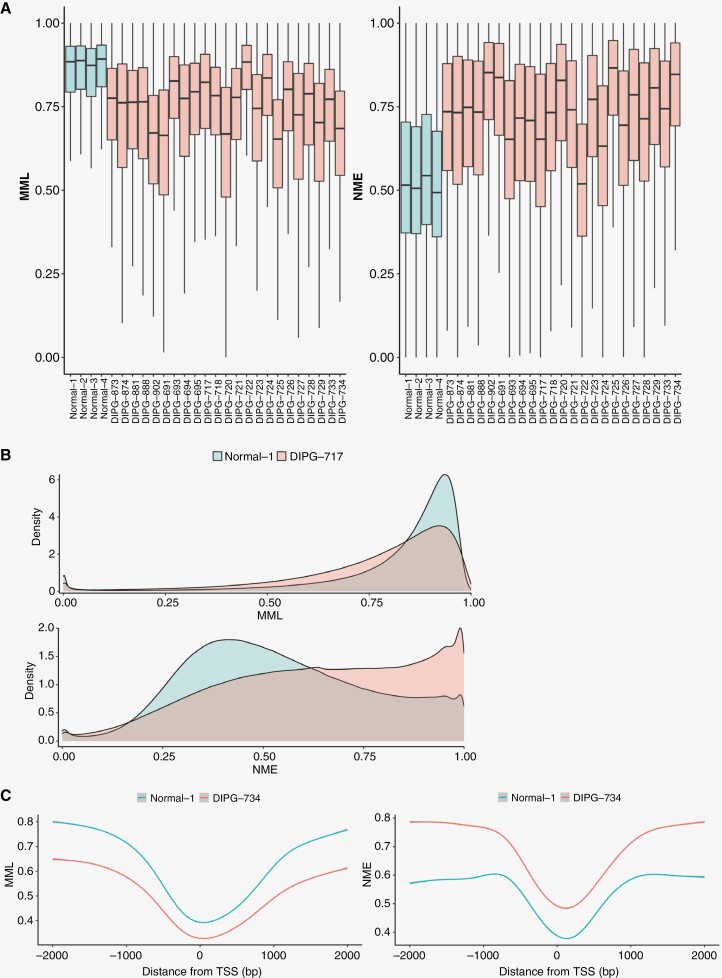
Global hypomethylation and increased epigenetic stochasticity in DIPG. (a) Genome-wide boxplots of mean methylation level (MML) and normalized methylation entropy (NME) in 4 normal fetal brain controls (normal 1-4; blue) and 23 primary DIPG patient samples (pink). Center line, median; box, interquartile range (IQR); whiskers, 1.5 × IQR. Median differences between DIPG samples and controls were evaluated using the Wilcoxon signed-rank test with *p*-values < 2.2E−16. (b) Genome-wide density distribution plots of NME and MML for a representative DIPG sample (DIPG-717) vs a normal fetal brain control (normal-1). (c) Mean MML (top) and NME (bottom) values in representative sample DIPG-734 and normal fetal brain control in promoters (± 2 kb from TSS).

WGBS and subsequent processing through the informME pipeline were performed on 23 primary DIPG patient samples, with participant information as listed in Supplementary Table 1, and on existing WGBS data from normal fetal brain samples. Methylation probability distributions within each genomic region are summarized using the mean methylation level (MML) and normalized methylation entropy (NME), and discordance in epigenetic stochasticity between two samples is quantified by the JSD. We evaluated genome-wide distributions of MML (Figure 2a, left) and NME (Figure 2a, right) for all DIPG and normal specimens. This revealed a dramatic shift toward global hypomethylation and elevated DNA methylation entropy in DIPG (*p* < 2.2E−16 for all comparisons; Wilcoxon signed-rank test). Density distributions of MML and NME for our representative sample, DIPG-717, further highlight this finding (Figure 2b), demonstrating a shift toward near-maximal NME values in a portion of the DIPG epigenome. This finding is consistent with our other primary patient samples, as shown in Supplementary Figure 1a and b. Additionally, we sought to assess changes in MML and NME as a function of distance from transcription start sites (TSS). A decrease in MML and an increase in NME were observed, shown for a representative sample in Figure 2c. This was confirmed with all other samples, as shown in Supplementary Figure 1c. These results reveal that, relative to normal controls, DIPG has a profound increase in DNA methylation stochasticity, with a genome-wide increase in methylation entropy.

### PEL Analysis Reveals that Notable Alterations in Methylation Patterns Map to Key Regulatory Regions

**Figure 3.**
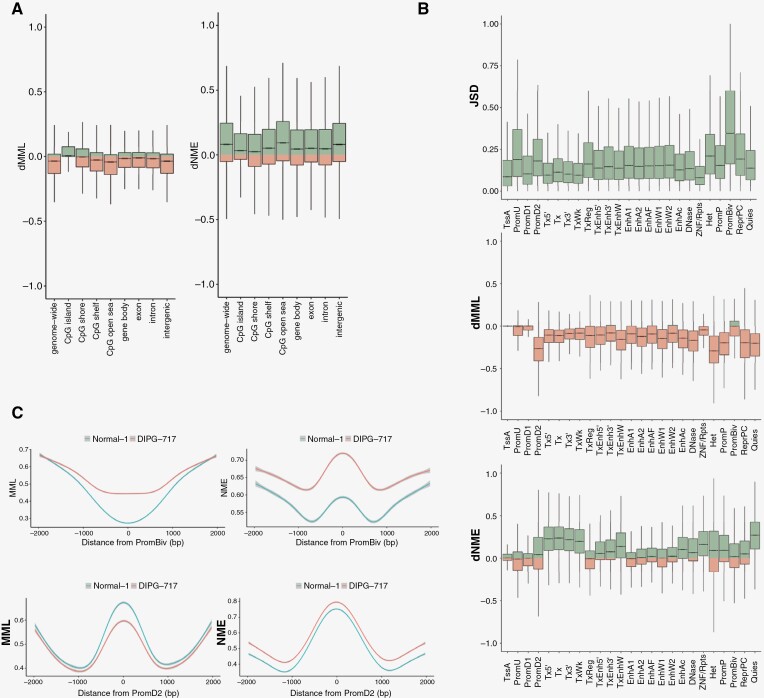
Alterations in DNA methylation stochasticity map to specific gene-regulatory regions. (a) Boxplots of dNME (right) and dMML (left) in DIPG-717 and normal fetal brain control shown genome-wide or at genomic features: CpG islands, shores, shelves, open seas, gene bodies, exons, introns, and intergenic regions. (b) Boxplots of dMML, dNME, and JSD for a representative DIPG vs normal fetal brain comparison (DIPG-717 and normal-1) within 25 ChromHMM genomic annotations. (c) MML and NME for DIPG (DIPG-717) and normal fetal brain over indicated regulatory regions: promoters downstream of TSS (PromD2), bivalent promoters (PromBiv).

Following global methylation analysis, we also sought to assess focal changes in MML, NME, and JSD over specific gene-regulatory regions. Evaluation of differential MML (dMML) and differential NME (dNME) between a DIPG sample and normal control over features related to gene regulation, shows that CpG islands exhibit hypermethylation in DIPG in contrast to hypomethylation of other genomic regions (Figure 3a). This is accompanied by increased DNA methylation entropy in DIPG. This finding is confirmed across other samples (Supplementary Figure 2a).

For a more granular analysis of the epigenetic landscape in DIPG, we also assessed dMML, dNME, and JSD over different classes of regulatory genomic elements, based on chromatin states as defined in ChromHMM annotations.^23^ Generally, most regulatory regions were hypomethylated in DIPG as compared to normal, with the “Promoter Downstream TSS 2” (PromD2) and “Heterochromatin” regions having the most dramatic shift towards hypomethylation. An exception was observed at bivalent promoters (PromBiv), which were relatively hypermethylated (Figure 3b). The most prominent increases in methylation entropy occurred over features associated with transcription (Tx5’, Tx, Tx3’, TxWk, TxEnh3’) and quiescent chromatin (Quies) (Figure 3b). These findings were generally consistent across samples (Supplementary Figure 2b).

JSD measures DNA methylation discordance between DIPG and normal. We found that upstream promoters (PromU), promoters downstream of TSS2 (PromD2), heterochromatic regions (Het), and bivalent promoters (PromBiv) exhibited the most notable methylation discordance (Figure 3b). We then visualized MML and NME patterns around the center of all PromBiv and PromD2 features, with MML showing hypermethylation at PromBiv and hypomethylation at PromD2, but NME demonstrating increased methylation entropy in both classes (Figure 3c, with additional samples shown in Supplementary Figure 2c). This analysis permits localization of DNA methylation stochasticity to gene-regulatory regions in DIPG.

### Genes Subjected to Increased Methylation Stochasticity Are Implicated in Key Pathways

After evaluating global and focal changes in methylation levels and methylation stochasticity, we sought to identify genes and pathways that serve as targets of methylation disruption in DIPG. We ranked all genes by methylation discordance between DIPG and normal over gene bodies and promoters.^19^ We evaluated for enrichments among the top 1000 genes in this list using gene set enrichment analysis (GSEA)^29,30^ (Supplementary Table 2). In the Molecular Signatures Database (MSigDB) curated gene sets (C2), we noted enrichments in genes marked by H3K27me3 in embryonic stem cells and targets of EED and SUZ12 of the Polycomb Repressive Complex 2 (PRC2). Among the Gene Ontology gene sets (C5) in MSigDB, we found enrichments in pathways associated with biological adhesion, synapse formation, neuron differentiation, and regulation of immune response. We used EnrichR to identify transcription factor targets and pathway enrichments.^30–32^ Notably, ENCODE and CHEA Consensus TFs from ChIP-X gene set library had consistent enrichment for SUZ12 targets.

Next, we ranked all genes from DIPG primary samples by highest JSD within 2 kb of the TSS. This allowed identification of genes and pathways with the greatest methylation discordance around promoter regions, whether driven by changes in mean methylation or entropy. We focused on the top 1000 genes in each sample based on their promoter JSD and subsequently looked for enrichments using GSEA and EnrichR (Supplementary Table 3).^29–32^ We noted enrichments in C2 gene sets related to embryonic stem cell promoter H3K27me3, and targets of EED and SUZ12 of the Polycomb Repressive Complex 2 (PRC2). C5 enrichments included gene sets involved in development and morphogenesis, neurogenesis, and neuron differentiation. EnrichR revealed enrichments in specific transcription factor networks related to PRC2 and developmental pluripotency. Notably, the ENCODE and CHEA Consensus TFs from ChIP-X gene set had consistent enrichment for SUZ12, EZH2, KLF4, SOX2, and NANOG targets. Additionally, the TRANSFAC and JASPAR PWMs revealed enrichments in our data set for targets of KLF11 and KLF4. Other sets revealed enrichments for key signaling pathways such as Wnt signaling, Hedgehog signaling, mTOR signaling, and signaling events related to neural crest differentiation as well as stem cell pluripotency (Supplementary Table 3).

After identifying enriched pathways, we focused on individual genes that consistently exhibited near-maximal JSD over their promoters across DIPG samples. While the relationship between promoter methylation level and gene expression is well known, our recent work in pediatric leukemia also demonstrated a relationship between DNA methylation entropy and the variability of gene expression as assessed by single-cell RNA-seq.^18^ JSD is a sensitive measure of methylation discordance which incorporates both mean changes and stochasticity. This motivated us to use JSD to identify epigenetically disrupted genes that may have functional roles in DIPG.

**Figure 4.**
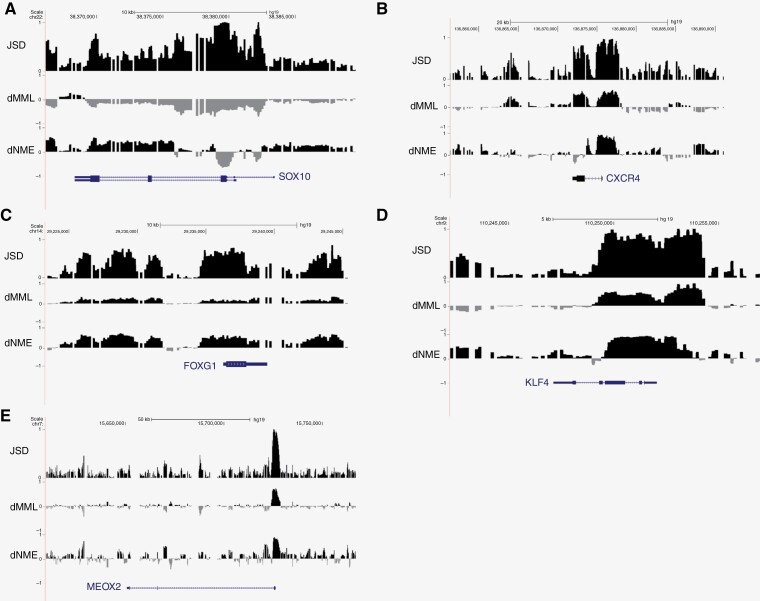
Examples of genes exhibiting methylation discordance between DIPG and normal fetal brain. (a–e) UCSC genome browser view showing differential mean methylation (dMML), differential methylation entropy (dNME), and methylation discordance (JSD) over genes that exhibit significant differences in DNA methylation stochasticity in DIPG as compared to normal fetal brain: *SOX10* (a), *CXCR4* (b), *FOXG1* (c), *KLF4* (d), and *MEOX2* (e).

One example that consistently ranked as a top gene (appearing among the top 100 genes by highest promoter JSD for 16 primary samples) was SOX10. We find that SOX10 exhibits consistent hypomethylation over both the promoter and the gene body compared to normal fetal brain control (Figure 4a).^33^ This is coupled with increased entropy over the gene body and over the promoter region. SOX10 is a transcription factor implicated in oligodendroglial differentiation and is overexpressed and hypomethylated in histone-mutant glioma, with a key functional role in viability, migration, and invasion of DIPG cells.^34,35^ Another gene of interest that was identified among the top genes by promoter JSD was CXCR4. CXCR4 has striking hypermethylation, increased JSD, and increased methylation entropy directly over the promoter region (Figure 4b). Previous gene expression studies identified CXCR4 as one of the most down-regulated genes in H3 K27M glioma as compared to H3 K27 wild-type pediatric high-grade gliomas.^8^ Likewise, FOXG1 was identified as having near maximal JSD among the primary samples and has been implicated as one of the most down-regulated genes in histone mutant glioma (Figure 4c). The near-maximal JSD over the FOXG1 gene is driven by increased promoter methylation accompanied by a dramatic increase in methylation entropy (Figure 4c). Similarly, KLF4 stood out as a top gene by JSD, and its methylation discordance was driven by hypermethylation accompanied by markedly increased methylation entropy (Figure 4d). KLF4 is a transcription factor with a prominent role in cellular dedifferentiation and stemness, functioning as one of the “reprogramming factors” used to induce pluripotency in differentiated cells.^36^ Interestingly, differential over-expression of KLF4 in a subset of ALDH + DIPG stem-like cells following MAPK or PI3K/mTOR inhibition was recently reported in DIPG, suggesting a potential mechanism of stress-induced plasticity.^37^ Finally, the homeobox transcription factor MEOX2 provides an example of focal epigenetic disruption in DIPG, with pronounced hypermethylation and increased methylation entropy specifically over its promoter (Figure 4e). It has been demonstrated that MEOX2 expression level is correlated with its methylation status in glioma samples and that downregulation of MEOX2 leads to increased viability in glioma stem cells through modulation of the ERK/MAPK pathway.^38^

### DNA Methyltransferase Inhibition by Decitabine Alters DNA Methylation Levels and Entropy Globally and at Key Target Genes

WGBS of primary DIPG samples indicates that DIPG is characterized by global DNA hypomethylation and increased methylation stochasticity. Additionally, DIPG is characterized by hypermethylation at genomic regions that are bivalent promoters in normal cells, hypomethylation at heterochromatin regions, and increased methylation entropy at transcription-regulatory regions. This suggests that DIPG has a preferred epigenetic configuration that might be responsive to pharmacological modulation of the DNA methylation machinery. We sought to investigate the responsiveness of DIPG DNA methylation landscapes and gene expression to DNA hypomethylating drugs.

**Figure 5.**
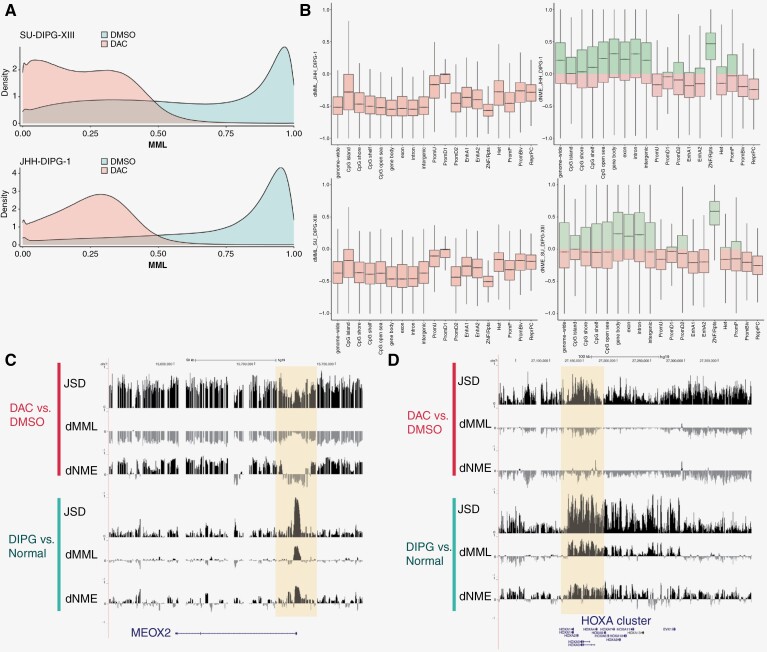
Gene expression in DIPG neurosphere cell lines is sensitive to pharmacologic modulation of DNA methylation. (a) Genome-wide density plots of MML in DIPG patient-derived neurosphere cell lines (SU-DIPG-XIII, upper panel; JHH-DIPG-1, bottom panel;) treated with DMSO (blue) or the hypomethylating agent decitabine (DAC; pink). (b) Boxplots showing differential mean methylation (dMML) and methylation entropy (dNME) over indicated genomic features following treatment with decitabine in two DIPG neurosphere cell lines (JHH-DIPG-1 and SU-DIPG-XIII). (c) UCSC Genome Browser view contrasting the DMR in MEOX2 for primary DIPG sample DIPG-717 vs normal fetal brain (green bar; as in Figure 4e), and in DIPG neurosphere cell line SU-DIPG-XIII following treatment with DAC vs DMSO control (red bar). The highlighted region showed hypermethylation and increased methylation entropy in DIPG vs normal, and coordinately shows hypomethylation and reduction in methylation entropy following DAC treatment. (d) UCSC Genome Browser view contrasting a DMR in the HOXA cluster on chromosome 7 for primary DIPG sample DIPG-717 vs normal fetal brain (green bar), and in DIPG neurosphere cell line SU-DIPG-XIII following treatment with DAC vs DMSO control (red bar). The highlighted region showed hypermethylation and increased methylation entropy in DIPG vs normal, and coordinately shows hypomethylation and reduction in methylation entropy following DAC treatment.

We subjected 4 patient-derived DIPG cell lines, JHH-DIPG-1, HSJD-DIPG-007, SU-DIPG-XIII, and SF7761 to treatment with 100 nM decitabine for 5 days followed by WGBS. This demonstrated a dramatic reduction in CpG methylation genome-wide, as compared to DMSO-treated controls (Figure 5a). While decitabine treatment resulted in hypomethylation genome-wide, the effect on methylation entropy in different genomic regions varied. Decitabine treatment led to dramatically increased methylation entropy over Zinc Finger Repeats (ZNF/Rpts) regions, but an overall decrease in entropy at active enhancers (Enh1, Enh2), promoter elements (PromU, PromD1, PromD2), heterochromatin, bivalent promoters (PromBiv), and Repressed Polycomb (ReprPC) (Figure 5b). This suggests that treatment with DAC might reverse elevated DNA methylation stochasticity over gene-regulatory regions in DIPG, possibly constraining the range of available gene expression states. Additionally, decitabine-induced hypomethylation and elevated entropy in ZNF/Rpts regions suggests the possibility of inducing expression of immunogenic targets such as endogenous retroviral elements, cryptic transcripts, and other normally suppressed neoantigens, potentially providing a therapeutic vulnerability in DIPG. This is in line with findings by Krug et al. that hypomethylating agents can induce the expression of endogenous retroviral elements.^39^

We sought to assess whether DAC treatment of DIPG cell lines decreases methylation entropy at genes that exhibited increased epigenetic stochasticity in primary DIPG samples. We first ranked the genes in our primary samples according to the greatest dNME within 2 kb of the TSS, as compared to a normal fetal brain, and then focused on the top 1000 genes in each set (Supplementary Table 4). We evaluated the ability of DAC to reduce methylation stochasticity at specific genes that were identified as targets of elevated NME in primary DIPG samples. MEOX2 was identified as a gene target with a reversal in DNA methylation entropy following DAC treatment. MEOX2 demonstrates hypomethylation and a focal decrease in methylation entropy over its promoter region following decitabine treatment (Figure 5c). This reduction in entropy at the promoter reflects a lower number of available methylation patterns, which in turn would constrain available gene expression states. Similarly, the HOXA cluster stood out as a set of genes that experienced near maximal methylation discordance and markedly increased methylation entropy in primary DIPG samples compared to normal brains. Following treatment with DAC, we noted a substantial decrease in methylation entropy over this region, as shown in Figure 5d. Previous studies have demonstrated that HOXA cluster genes are aberrantly expressed in DIPG, and that MEOX2 and numerous HOXA cluster genes are among the transcription factors correlated with patient survival in glioma.^38,40^ In summary we show that DNA hypomethylating drug treatment can reverse focal epigenetic dysregulation that is characteristic of DIPG.

### Gene Expression in DIPG Patient Derived Neurosphere Cell Lines Is Sensitive to Pharmacologic Modulation of DNA Methylation

**Figure 6.**
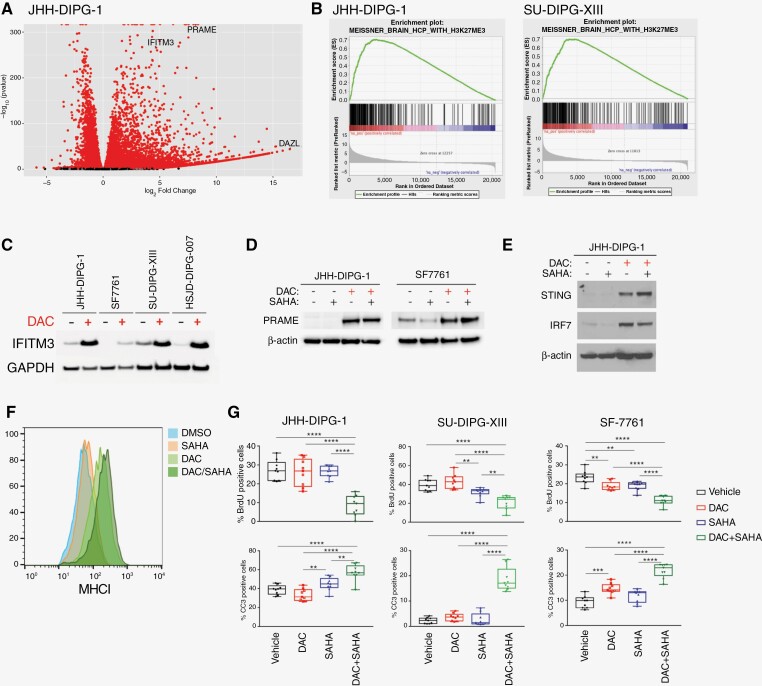
Pharmacologic modulation of DNA methylation in DIPG neurosphere cell lines alters gene expression, immune signaling, and survival. (a) Volcano plot of RNA-seq data for decitabine vs DMSO treatment of JHH-DIPG-1 cells. Red indicates FDR-adjusted *p*-value < .05, black indicates a difference that is not significant. IFITM3, PRAME, and DAZL were noted to be significantly upregulated following decitabine treatment. (b) Polycomb repressive complex 2 related gene sets are among the top gene set enrichment analysis (GSEA) results for differential gene expression (ordered by log2 fold change, from greatest to least) among DAC vs DMSO treated JHH-DIPG-1 (left) and SU-DIPG-XIII cells (right). (c) Western blot analysis of IFITM3 expression in the indicated DIPG cell lines following treatment with 100 nM decitabine (DAC) or control (DMSO). GADPH is shown as a loading control. (d) Western blot analysis of PRAME expression in the indicated DIPG cell lines following treatment with 100 nM decitabine (DAC) or control (DMSO) with or without SAHA, as indicated. Beta-actin is shown as a loading control. (e) Western blot analysis of IRF7 and STING expression in the JHH-DIPG-1 cell line following treatment with 100 nM decitabine (DAC) or control (DMSO) with or without SAHA, as indicated. Beta-actin is shown as a loading control. (f) Flow cytometry showing increased expression of MHC Class-I following treatment with 100 nM decitabine (DAC) or control (DMSO) with or without SAHA, in HSJD-DIPG-007 cells. (g) Quantification of cells positive for BrdU (top) or Cleaved Caspase 3 (CC3; bottom), in three patient-derived DIPG cell lines, JHH-DIPG-1, SF7761, and SU-DIPG-XIII, following treatment with vehicle, DAC, SAHA, or combination DAC and SAHA.

Following confirmation that DAC alters the DNA methylation landscape in DIPG cell lines, we investigated its effect on gene expression using RNA-seq. The volcano plot for JHH-DIPG-1 (Figure 6a) indicates a robust change in gene expression following treatment with DAC. Some of the top upregulated genes included Interferon Induced Transmembrane Protein 3 (IFITM3), Preferentially Expressed Antigen in Melanoma (PRAME), and Deleted in Azoospermia-like (DAZL) (Supplementary Table 5). PRAME is a cancer testis antigen (CTA) that is expressed in germline tissues, but not differentiated tissues, and has been investigated as a target for cancer immunotherapy in extracranial pediatric malignancies.^41^ Similarly, expression of gamete-specific genes including DAZL has been reported upon deletion of DNA methyltransferases in murine embryonic stem cells, consistent with our findings of its upregulation upon decitabine-induced hypomethylation.^42^

We next wanted to investigate concrete ways in which decitabine treatment impacts methylation levels and gene expression. Previous studies by us and others have shown that the DNA methylation level at promoters is related to gene expression level and variability in cancer.^21^ We investigate this relationship in DIPG cell lines genome-wide by exploring the relationship between mean methylation level around gene promoters and gene expression levels. We find that, as expected, promoter hypomethylation is linked to higher gene expression (Supplementary Figure 3a).

Accordingly, we find that decitabine-induced demethylation at gene promoters can lead to increased gene expression. This is shown for CXCR4, a gene that we show in Figure 4 is downregulated and hypermethylated in primary DIPG samples. Following treatment with DAC, the CXCR4 promoter is demethylated and gene expression is dramatically upregulated (Supplementary Figure 3b and c). A similar direct effect is observed for CDKN2a and CDKN2b. CDKN2a/b has previously been shown to be hypermethylated and downregulated in models of H3 K27M glioma.^43^ We show that decitabine induces hypomethylation over the CDKN2a/b promoters, accompanied by significant over-expression (Supplementary Figure 3c and d). This is supported by prior work by Becher et al. that H3K27M-induced suppression of p16 in DIPG is mediated by DNA hypomethylation in a mouse model. Decitabine treatment can similarly induce the expression of potential neoantigens, such as PRAME and DAZL (Figure 5a). We also find that their expression is accompanied by marked hypomethylation following decitabine treatment (Supplementary Figure 3f and g).

Aside from direct promoter demethylation by decitabine, hypomethylating drugs have been shown to impact gene expression through an indirect mechanism. Work by Baylin et al. has demonstrated that hypomethylating agents lead to the induction of endogenous retroviral elements (ERVs) and downstream induction of interferon and innate immune signaling.^44,45^ This has also been shown to be particularly important in DIPG due to aberrant retention of activating histone acetylation marks over ERVs.^39^ We hypothesize that the up-regulation of interferon genes, such as IFITM3, that we see in our RNA-Seq results is the result of indirect stimulation by ERVs. Indeed, we find that ERV genes, such as ERVMER34-1 and ERVW-1 are hypomethylated and upregulated after decitabine treatment (Supplementary Figure 4a–d). These findings suggest that treatment with hypomethylating agents induces alterations in gene expression in both direct and indirect manners. Many of these alterations occur in pathways related to immune activation and neoantigen expression, which may be therapeutically relevant.

We additionally investigated pathways or gene sets enriched among genes differentially expressed following decitabine treatment of DIPG cell lines. Gene set enrichment analysis (GSEA) of genes ordered by log2 fold change in the curated (C2) gene set library revealed enrichments for gene sets relevant to PRC2 regulation, such as Meissner Brain H3K27me3 genes (shown for JHH-DIPG-1 and SU-DIPG-XIII in Figure 6b). This gene set highlights genes that have H3K27me3 and high CpG density in normal brain.^29,30^ Given that we also demonstrated that bivalent promoters are hypomethylated (Figure 5b), this finding is consistent with the interplay between histone methylation and DNA methylation in the regulation of bivalent gene expression.^46^ We further assessed changes in expression at the protein level. JHH-DIPG-1, HSJD-DIPG-007, SU-DIPG-XIII, and SF7761 cells were harvested 72 h after a 5-day course of 100 nM decitabine. Western blot demonstrates that DAC treatment dramatically induced protein levels of the immune marker Interferon-Induced Transmembrane Protein 3 (IFITM3) (Figure 6c).

Following our observation that DAC could induce robust changes in gene expression and immune signaling in DIPG, we wanted to assess whether DAC could synergize with other epigenetic-targeted agents to enhance this response. Previous drug screening efforts in DIPG/DMG identified vorinostat (suberoylanilide hydroxamic acid, SAHA) an HDACi, as a candidate.^47,48^ Additionally, it has been shown that H3 K27M DIPG aberrantly retains histone acetylation marks over ERVs and repetitive elements, supporting a rationale for combining hypomethylating drugs and HDAC inhibitors.^39^ Work in other cancers suggests that HDAC inhibition synergizes with DNA hypomethylation in inducing ERV reactivation/viral mimicry and downstream immune activation^44^ This led us to investigate whether hypomethylation of DIPG cells with decitabine primes for further cytotoxic effects of HDAC inhibition by SAHA. DIPG neurosphere cell lines were subjected to a 5-day treatment with 100 nM decitabine or DMSO vehicle (replaced every 24 hours), and thereafter treated with DMSO control or 300 nM SAHA for an additional 72 h. The Western blot in Figure 6d shows that DAC treatment dramatically increases the expression of the cancer testis antigen protein PRAME, in agreement with its differential over-expression following decitabine treatment in our RNA-Seq data. Consistent with activation of innate immune signaling and interferon signaling, we also found that DAC dramatically induces expression of Interferon Regulatory Factor 7 (IRF-7) and Stimulator of Interferon Genes (STING) (Figure 6e). Importantly, STING activation has been shown to promote NK cell-mediated antitumor response and tumor regression in glioblastoma models.^49^

Additionally, we investigated whether DAC, alone or in combination with SAHA, would increase the level of MHC class I expression in this immunologically “cold” tumor. Using flow cytometry, we demonstrate that DAC treatment, alone and in combination with SAHA, increases MHC class I expression in DIPG cells (Figure 6f). DNMT inhibition has been shown to induce upregulation of MHC Class I in other cancer types.^50^ Together, these data demonstrate that pharmacologic modulation of DNA methylation in DIPG can have a profound impact on gene expression and immunogenicity of DIPG.

We next investigated whether treatment with DAC and SAHA could decrease cell viability or induce apoptosis. DIPG neurosphere cell lines were again subjected to the previous 5 day treatment with 100 nM decitabine or DMSO vehicle, with or without 300 nM SAHA for an additional 72 h. We then quantified cells positive for BrdU as a measure of proliferation and Cleaved Caspase 3 staining as a measure of apoptosis. We observed a statistically significant reduction in proliferation with combination of DAC/SAHA, relative to all other treatment conditions (Figure 6g). Similarly, a significant increase in Cleaved Caspase 3 staining, was shown for combination DAC/SAHA relative to all treatment conditions (Figure 6g).

## Discussion

Previous work has implicated the mutant histone as a key driver of epigenetic dysregulation in DIPG. Prior work has shown that H3K27M mutation leads to a global reduction in H3K27me3 accompanied by global DNA hypomethylation, together resulting in an aberrant gene expression signature.^8,51^ We build on these prior studies to map the dynamic DNA methylation landscape to capture epigenetic stochasticity, which underlies cellular heterogeneity and plasticity. This can identify ways in which DIPG cells erode epigenetic barriers to dedifferentiation and phenotypic plasticity.^11,13^ Since the histone mutation drives but is not sufficient for tumorigenesis, this analysis is also crucial for identifying epigenetically altered driver genes that serve as the link between histone mutation and malignant transformation.^7,52^

To address these gaps, we performed WGBS on a large set of DIPG samples (23 primary patient samples and 4 patient-derived neurosphere cell lines), generating an important resource for dissecting epigenetic regulation in this disease. We used the informME analysis pipeline to construct DNA methylation potential energy landscapes, allowing comprehensive quantification of methylation stochasticity genome-wide. Our analysis reveals that DIPG cells have a stochastically disordered methylation landscape, with markedly increased DNA methylation entropy as compared to normal controls. We found that epigenetic disruption as measured by JSD mapped most strongly to focal regulatory regions, including promoters downstream of TSSs (PromD2), heterochromatic regions, and bivalent promoters (PromBiv). This was driven by focal hypomethylation at downstream promoters and heterochromatic regions, hypermethylation at bivalent promoters, and elevated methylation entropy. We found an enrichment of epigenetic dysregulation in pathways and genes associated with neurogenesis and neural differentiation, providing a resource for further investigation of the altered dynamic epigenetic landscape in DIPG.

Our prior work in other cancer types showed that increased methylation entropy at gene promoters is linked to increased variability of gene expression.^21^ We hypothesized that the gene expression profile of DIPG would be sensitive to pharmacological modulation of the epigenome. We employed the DNA hypomethylating agent decitabine and found that it induces profound genome-wide hypomethylation and can reverse locally increased methylation entropy at specific regulatory regions and genes, such as MEOX2 and the HOXA cluster. Given the contribution of epigenetic variability to tumor evolution and plasticity that can underlie therapeutic resistance, agents that can reduce the number of epigenetic configurations and gene expression states available to DIPG cells can constrain DIPG plasticity for therapeutic benefit.

We demonstrate a wide-ranging impact of decitabine on gene expression in DIPG neurospheres, by both direct and indirect mechanisms. Demethylation of hypermethylated promoters can lead to re-expression of suppressed genes, as shown for CXCR4 and CDKN2a/b. Regulation of CDKN2a/b by promoter methylation is supported by studies in a mouse model by Becher and colleagues, showing that H3 K27M suppresses p16 via a mechanism responsive to hypomethylating drugs.^43^ Among genes whose expression is most upregulated following decitabine treatment, the highest ranking gene set enrichments are genes that in normal brains have H3 K27me3 methylation and high CpG density. Interestingly, our WGBS analysis found that chromatin regions that are normally bivalent are uniquely hypermethylated in DIPG, in a background of overall hypomethylation. Given the inhibition of H3 K27 trimethylation by the H3 K27M mutation,^7^ and the known mechanistic interplay between histone methylation and DNA methylation in regulating the expression of bivalent genes,^46^ this suggests a mechanism by which decitabine treatment can specifically induce expression of repressed bivalent regions, which would warrant further detailed investigation including ChIP-seq studies.

Treatment with decitabine led to activation of genes related to interferon and immune signaling, consistent with prior work,^39^ and induced aberrant expression of germ cell-specific genes and putative neoantigens. Induction of cancer testis antigens and neoantigens is of clinical significance. We show that putative novel antigens can be induced by decitabine-mediated demethylation, as shown for DAZL and PRAME. A phase I study of PRAME-targeted T-cell infusion for aggressive pediatric brain tumors is currently accruing patients (NCT03652545). Given that DIPG is classically recognized as an immunologically cold tumor, re-expression of germ cell-specific genes and neoantigens could facilitate immune targeting of DIPG. Increased expression of STING following decitabine treatment could also be therapeutically relevant, potentially increasing sensitivity to radiation and cell therapies.^53,54^ STING activation has been shown to promote NK cell-mediated antitumor responses in glioblastoma models, such that use of a STING agonist led to upregulation of NK cell and T-cell infiltration and subsequent tumor cell killing, while in vivo depletion of the NK cell population abolished the efficacy of the STING agonist. Our results show that DAC leads to pronounced STING activation, suggesting a potential influence on NK cell-mediated cell killing. Further work, particularly using immunocompetent in vivo models, will be necessary to fully address these mechanisms. Taken together, our data supports the investigation of decitabine pre-treatment in combination with cell-based immunotherapies, immune checkpoint inhibitors, or conventional chemotherapy and radiotherapy.^55^

## Supplementary Material

vdae023_suppl_Supplementary_Table_S1

vdae023_suppl_Supplementary_Materials

vdae023_suppl_Supplementary_Figure_S1

vdae023_suppl_Supplementary_Figure_S2

vdae023_suppl_Supplementary_Table_S2

vdae023_suppl_Supplementary_Table_S3

vdae023_suppl_Supplementary_Table_S4

vdae023_suppl_Supplementary_Table_S5

vdae023_suppl_Supplementary_Figure_S3

vdae023_suppl_Supplementary_Figure_S4

## Data Availability

The datasets generated in the current study are available in the Gene Expression Omnibus and Sequence Read Archive repositories, with accession number GSE224500.
