# Supplementary material for: DNA methylation landscapes in DIPG reveal methylome variability that can be modified pharmacologically
Source: Neurooncol Adv. 2024 Feb 19;6(1):vdae023. doi: 10.1093/noajnl/vdae023 (PMC10926944; doi:10.1093/noajnl/vdae023)
Supplement: vdae023_suppl_Supplementary_Materials [file vdae023_suppl_supplementary_materials.docx]

**Supplementary Material**

*Supplementary Methods*

Sample preparation and nucleic acid extraction

Primary patient samples were obtained from the Children's Brain Tumor Network (CBTN) at the Children’s Hospital of Philadelphia (CHOP). Sample characteristics are provided in Table S1. For cell lines, genomic DNA isolation was carried out using the MasterPure DNA Purification kit (Epicentre). RNA extraction was carried out using the RNeasy Mini kit (Qiagen), including on-column DNase I digestion (Invitrogen). Integrity of genomic DNA was confirmed by gel electrophoresis. RNA was assessed on an Agilent 2100 Bioanalyzer using the Agilent RNA 6000 Nano kit.

WGBS library preparation and sequencing

WGBS library preparation and sequencing were carried out as previously described [24]. Briefly, WGBS single indexed libraries were generated using NEBNext Ultra DNA library Prep kit for Illumina (New England BioLabs). 500 ng input gDNA was quantified by Qubit dsDNA BR assay (Invitrogen) and spiked with 1% unmethylated Lambda DNA (Promega, cat # D1521) to monitor bisulfite conversion efficiency. Input gDNA was fragmented by Covaris S220 and LE220 Focused-ultrasonicator to an average insert size of 350 bp. Size selection was performed using AMPure XP beads and insert sizes of 300-400bp were isolated. Samples were bisulfite converted after size selection using EZ DNA Methylation-Gold Kit or EZ DNA Methylation-Lightning Kit (cat#D5030) following the manufacturer’s instructions. Amplification was performed after the bisulfite conversion using Kapa Hifi Uracil+ (Kapa Biosystems, cat# KK282) polymerase based on the following cycling conditions: 98°C 45s / 8cycles: 98°C 15s, 65°C 30s, 72°C 30s / 72°C 1 min. AMPure cleaned-up libraries were run on the 2100 Bioanalyzer (Agilent) High-Sensitivity DNA assay, and samples were also run on the Bioanalyzer after shearing and size selection for quality control. Libraries were quantified by qPCR using the Library Quantification Kit for Illumina sequencing platforms (Kapa Biosystems, cat#KK4824) and the CFX384 Real-Time PCR Detection System( Bio-Rad). WGBS libraries were sequenced on an Illumina HiSeq4000 instrument using 150 bp paired end indexed reads and 5% of non-indexed PhiX library control (Illumina). Coverage is indicated in Supplementary Table 1.

FASTQ files were processed using Trim Galore! v0.3.6 (Babraham Institute) to perform single-pass adapter- and quality-trimming of reads. FastQC v0.11.2 was employed for quality control of reads. Reads were aligned to the hg19/GRCh37 genome using Bismark v0.14.5 and Bowtie2 v2.2.6. Separate M-bias plots for read 1 and read 2 were generated by running the Bismark methylation extractor using the ‘mbias_only’ flag, and these plots were used to determine how many bases to remove from the 5’ end of reads. The number was generally higher for read 2, known to exhibit a lower quality. The amount of 5’ trimming ranged from 4 bp to 20 bp, consistent with Jenkinson *et al.* [20]. BAM files were subsequently processed with Samtools v0.1.19 for sorting, merging, duplicate removal, and indexing.

RNA sequencing

Strand specific mRNA libraries were generated using the NEBNext Ultra II Directional RNA library prep Kit for Illumina (New England BioLabs #E7760), mRNA was isolated using Poly(A) mRNA magnetic isolation module (New England BioLabs #E7490). Preparation of libraries followed the manufacturer’s protocol (Version 1.0 4/17).  Input was 1000ng and samples were fragmented for 15 min for RNA insert size of ~200 bp.  The following PCR cycling conditions were used: 98°C 30s / 8 cycles: 98°C 10s, 65°C 75s / 65°C 5 min. Stranded mRNA libraries were sequenced on an Illumina HiSeq4000 instrument,  using 47bp paired-end dual indexed reads and 1% PhiX control. mRNA sequencing depth ranged from 90-125M reads. We aligned reads to GRCh37 using STAR version 2.4.2a [7] with the following options --readFilesCommand zcat --outSAMtype BAM Unsorted SortedByCoordinate --quantMode TranscriptomeSAM GeneCounts. We generated summarized experiment (se) objects using the gtf file Homo_sapiens.GRCh37.75.gtf and the following command from the Bioconductor package ‘GenomicAlignments’: summarizeOverlaps(features=exonsByGene, reads=bamfiles, mode="Union", singleEnd=FALSE, ignore.strand=FALSE, fragments=TRUE).

Genomic features and annotations

Genomic features and annotations were defined as previously described [24]. Files and tracks utilize genomic coordinates for hg19. CGIs were obtained from Wu *et al.* [50]. CGI shores were defined as sequences flanking 2-kb on either side of islands, shelves as sequences flanking 2-kb beyond the shores, and open seas as everything else. The R Bioconductor package ‘TxDb.Hsapiens.UCSC.hg19.knownGene’ was used for defining genes, exons, introns, and gene bodies. The promoter region of a gene was defined as the 4-kb window centered at the TSS and the gene body region was defined as the remainder of the gene. Chromatin functional annotations were obtained using the ChromHMM 25-state reference model [8]. ChromHMM 25-state enhancer and promoter annotations with definitions and emission parameters as previously described were employed [9].

WGBS analysis and PEL computation

We computed DNA methylation potential energy landscapes (PELs) from WGBS data using informME (v0.3.3), a freely available information theoretic pipeline for methylation analysis based on the one-dimensional Ising model of statistical physics, with methods previously described [24]. As previously described, genomic regions of length 3-kb were used for parameter estimation; to avoid statistical overfitting, this approach does not model regions that contain fewer than 10 CpG sites, for which less than 2/3 of the CpG sites were observed, or for which the average depth of coverage was less than 2.5 observations per CpG site. After dividing the genome into non-overlapping genomic windows of 3-kb for PEL construction, we further partitioned each 3-kb estimation window into 20 non-overlapping analysis regions of size 150 bp each. Within each analysis region, we computed the probability distribution of the methylation level, through the mean methylation level (MML) and the normalized methylation entropy (NME), directly from the associated PEL using informME, as previously described [24]. The normalized methylation entropy is a normalized version of Shannon’s entropy, calculated as described, and used to quantify the amount of methylation stochasticity observed within an analysis region. It ranges between 0 and 1, achieving its maximum value when all methylation levels within an analysis region are equally likely (i.e., fully stochastic methylation), and achieving its minimum value only when a single methylation level is observed (i.e., perfectly ordered methylation). We computed the Jensen-Shannon distance (JSD) between two probability distributions of the methylation level in a test (DIPG) and a reference (normal) sample within an analysis region by methods previously described [24]. The JSD ranges between 0 and 1, taking its minimum value only when the two probability distributions are identical, in which case no statistical discordance in methylation level is present, and its maximum value of 1 only when the supports of the two probability distributions do not intersect each other, in which case a maximum statistical discordance in methylation level is observed.

Differential methylation analysis

We performed differential analysis between test (DIPG) and reference (normal fetal brain) WGBS samples using informME [19]. Within a given region of analysis, we computed Jensen-Shannon distances (JSDs) between the corresponding methylation level probability distributions, as well as differences between mean methylation levels (dMMLs) and normalized methylation entropies (dNMEs). Gene and genomic feature ranking by methylation discordance was achieved using the ‘jsGrank’ utility of informME, which uses the JSD to identify regions of the genome with statistically significant discordance in methylation stochasticity. In a single test/reference (DIPG/normal) comparison, we computed, within the promoter and body regions of each gene in the genome, the magnitude of the Jensen-Shannon distance (JSD), calculated as the square root of the average of the squared JSD values within all analysis regions that overlap each feature. As previously described, we performed hypothesis testing to test against the null hypothesis that the JSD magnitude within a particular genomic feature (promoter or body) can be explained by normal technical, statistical, or biological variability. We did so by empirically constructing a null distribution for the values of all JSD magnitudes genome-wide, which we obtained by comparing between biological replicates of normal samples [19, 25]. Each gene is scored using the computed *p*-value for rejecting the null hypothesis and produced a ranked list of genes with increasing *p*-values; statistical significance of each ranking was evaluated while controlling for the false-discovery rate (FDR) at 0.05 using *q*-values computed by the Benjamini-Hochberg (BH) procedure.

Cell Culture

The JHH-DIPG-1 cell line was established from a rapid autopsy sample, as previously described [44]. The HSJD-DIPG-07 cell line was provided by Dr. Montero Carcaboso (Hospital Sant Joan de Deu Barcelona, Spain) [45]. The SF7761 cell line was a gift from Dr. Hashizume (Northwestern University, Chicago, IL) [16]. The SU-DIPG-XIII cell line was kindly provided by Michelle Monje (Stanford University School of Medicine, Stanford, CA) [12]. All lines were ensured to be mycoplasm-free via PCR testing. Cell lines were cultured in “EF media”: 30% Hams F12 [Gibco 11765-062], 70% DMEM [Gibco 11965-118], 1% PSA [Gibco-BRL 15420-062], 2% B27 [Gibco-BRL 17504-044], 20 ng/ml EGF [Peprotech AF-100-15], 20 ng/ml FGF-2 [Peprotech 100-18B], and 5 micrograms/ml heparin [Millipore Sigma H3149]. All cell lines were kept in 25 cm^2^ culture flasks at 37°C and 5% CO_2_.

Drug Treatments

The hypomethylating agent, 5-aza-2’-deoxycitidine (Decitabine [DAC]; Cayman Chemical; #11166), and the HDACi, suberoylanilide hydroxamic acid (Vorinostat [SAHA]; Cayman Chemical; #10009929) were used in our cell treatments. Cells were treated with 100 nm DAC or DMSO for 5 days, with media and drug change every 24 hours. For the RNA-Seq and WGBS experiments, the DMSO and DAC treated cells were harvested at the 72-hour time point (day 8).

For flow cytometry, apoptosis, and proliferation assays, 300 nm SAHA was added on the fifth day to one of the DMSO treated conditions and one of the 5-day DAC treated conditions following drug and media change. Cells were harvested at the 72-hour point.

Flow Cytometry - Approximately 1x10^6^ cells were plated into T25 flasks with EF media and supplemented with no treatment, DMSO (vehicle), Decitabine, Vorinostat, Decitabine and Vorinostat, or IFNγ prior to staining for flow cytometry. Cells were stained with LIVE/DEAD® Fixable Near-IR Dead Cell Stain Kit, for 633 or 635 nm excitation (Fisher Scientific) at a concentration of 1:1500 at RT in PBS for 30 min. They were subsequently washed and then stained with Percp/Cy5.5-conjugated HLA A, B, C (Clone W6/32, BioLegend, San Diego, CA, USA) or Percy/Cy5.5 labeled mouse IgG2 kappa isotype antibody (BioLegend, San Diego, CA, USA) at 1:200 at RT for 15 minutes. Cells were analyzed using a BD FACS Calibur (Becton Dickinson, Franklin Lakes, New Jersey, USA) and FlowJo software (Tree Star, Ashland, Oregon, USA).

Analysis of protein expression by western blotting and cell viability by Bromodeoxyuridine (BrdU) Incorporation Detection and Cleaved-Caspase-3 Immunofluorescence Detection

At the 72-hour time point, cells were harvested by centrifugation at 3.3 rcf for 5 minutes at 4°C, washed twice in cold 1X PBS pH 7.4 (Quality Biological) by centrifugation at 3.3 rcf at 4°C for 5 minutes, flash-frozen in liquid nitrogen and stored at -80°C. Cell pellets were resuspended in Mammalian Lysis Buffer (50mM Tris-HCl pH 7.4, 150 mM NaCl, 1% Triton X-100, 10% glycerol, supplemented with cOmplete EDTA-free Protease Inhibitor Cocktail tablet [Roche]) for 60 minutes. Lysed cells were then spun down at 16.2 rcf and 4°C for 5 minutes and the whole cell lysate was used. Protein concentrations were assessed using the Pierce Coomassie Plus (Bradford) Protein Assay Kit (Thermo). Primary antibodies were used as follows: IFITM3 (1:1000; Cell Signaling; #59212), GAPDH (1:10,000; Santa Cruz; #SC-47724), PRAME (1:1000; Millipore Sigma, HPA041153), IRF7 (1:1000; Cell Signaling, #13014), STING (1:1000; Cell Signaling, #13647), beta-actin (ThermoFisher, MA1-140). Bromodeoxyuridine (BrdU) assays and cleaved caspase 3 immunofluorescence detection were performed as previously described with the following modifications [38] [48]. Cells were pulsed with BrdU (Sigma-Aldrich) for 6 hours, centrifuged, and washed with cold PBS. The pellet was resuspended in cytospin collection fluid (ThermoFisher Scientific) then cells were spun onto slides. After washing with PBST (1x PBS Tween-20), cells were permeabilized with 0.1% TritonX/PBST for 15 minutes, washed with PBST, and blocked with 5% normal goat serum/PBST. For BrdU, cells were denatured with 2N HCl for 30 minutes prior to blocking. BrdU primary antibody (Cell Signaling; #5292) was used at 1:500 dilution for 1 hour at 37°C. Following three washes with PBST, cells were incubated with Cy-3 anti-mouse secondary (Jackson ImmunoResearch) at 37°C for 45 minutes at 1:500 dilution. Cleaved caspase-3 primary antibody (Cell Signaling; #9661) was used at 1:400 dilution for 1 hour at room temperature or overnight at 4°C. After being washed with PBST, cells were incubated with Cy-3 anti-rabbit secondary (Jackson ImmunoResearch) at room temperature for 45 minutes at 1:500 dilution. Once washed, cells were counterstained with DAPI (Roche) at 1:1000 dilution, mounted with anti-fade (ThermoFisher Scientific), and visualized by fluorescent microscopy.

**Supplementary Tables**

Supplementary Table 1: DIPG Sample Annotation and WGBS Statistics for all primary patient samples.

Supplementary Table 2: Top 1000 genes by methylation discordance at promoters and gene bodies between all DIPG samples and normal fetal brain, utilizing jsGrank output if informME, and gene set enrichment analyses within these 1000 genes.

Supplementary Table 3: Top 1000 genes ranked by highest JSD over promoter regions +/- 2 kb of the TSS for each DIPG sample as compared to normal fetal brain, and GSEA enrichment analysis for representative sample DIPG-717.

Supplementary Table 4: Top 1000 genes ranked by dNME +/- 2kb of the TSS in all primary patient samples as compared to normal fetal brain control.

Supplementary Table 5a-d: RNA-seq differential gene expression for all cell lines (JHH-DIPG-1, HSJD-DIPG-007, SF7761, SU-DIPG-XIII) for DAC treatment vs DMSO control.

**Supplementary Figures**

Supplementary Figure 1: Evaluation of mean methylation and methylation entropy in all DIPG primary samples.

a) Genome-wide density plots of MML for all DIPG primary patient samples versus a normal fetal brain control (Normal-1).

b) Genome-wide density plots of NME for all DIPG primary patient samples versus a normal fetal brain control (Normal-1).

c) Mean MML (left) and NME (right) values in all DIPG primary patient samples and normal fetal brain control at CpG sites in promoter regions (+/- 2 kb from TSS).

Supplementary Figure 2: Evaluation of differential methylation and differential methylation entropy for all DIPG primary samples as compared to normal fetal brain.

a) Boxplots of dMML and dNME in all DIPG primary patient samples and normal fetal brain control, Normal-1, genome-wide, and within CpG islands, shores, shelves, open sea, gene bodies, exons, introns, and intergenic regions.

b) Boxplots of Distributions of dMML, dNME, and JSD values observed in all DIPG primary patient samples and a normal fetal brain comparison (Normal-1) within 25 ChromHMM genomic annotations.

c) Mean MML and NME values in all DIPG primary patient sample and a normal fetal brain sample (Normal-1) at CpG sites within promoters downstream of TSSs (PromD2) and bivalent promoters (PromBiv).

Supplementary Figure 3: Relationship of DNA methylation and gene expression in DIPG neurosphere cell lines.

a) Mean MML upstream of the transcription start site (+/- 2 kb) as it relates to gene expression quartiles (0-25%, 25-50%, 50-75%, 75-100%).

b) UCSC genome browser view showing methylation discordance (JSD), differential mean methylation (dMML), and differential methylation entropy (dNME) over CXCR4 after treatment with decitabine.

c) Expression plot by RNA-Seq counts of CXCR4 before and after treatment of neurosphere cell lines with DAC.

d) UCSC genome browser view showing methylation discordance (JSD), differential mean methylation (dMML), and differential methylation entropy (dNME) over CDKN2a/b after treatment with decitabine.

e) Expression plot by RNA-Seq counts of CDKN2a and CDKN2b before and after treatment of neurosphere cell lines with DAC.

f) UCSC genome browser view showing methylation discordance (JSD), differential mean methylation (dMML), and differential methylation entropy (dNME) over DAZL after treatment with decitabine.

g) UCSC genome browser view showing methylation discordance (JSD), differential mean methylation (dMML), and differential methylation entropy (dNME) over PRAME after treatment with decitabine.

Supplementary Figure 4: Decitabine Treatment Induces Hypomethylation and Upregulation of ERVs

a) UCSC genome browser view showing methylation discordance (JSD), differential mean methylation (dMML), and differential methylation entropy (dNME) over ERVMER34-1 after treatment with decitabine.

b) Expression plot by RNA-Seq counts of ERVMER34-1 before and after treatment of neurosphere cell lines with DAC.

c) UCSC genome browser view showing methylation discordance (JSD), differential mean methylation (dMML), and differential methylation entropy (dNME) over ERVW-1 after treatment with decitabine.

d) Expression plot by RNA-Seq counts of ERVW-1 before and after treatment of neurosphere cell lines with DAC.
