# Supplementary figures and images for: DNA methylation landscapes in DIPG reveal methylome variability that can be modified pharmacologically
Source: Neurooncol Adv. 2024 Feb 19;6(1):vdae023. doi: 10.1093/noajnl/vdae023 (PMC10926944; doi:10.1093/noajnl/vdae023)

Supplementary Figure 1A

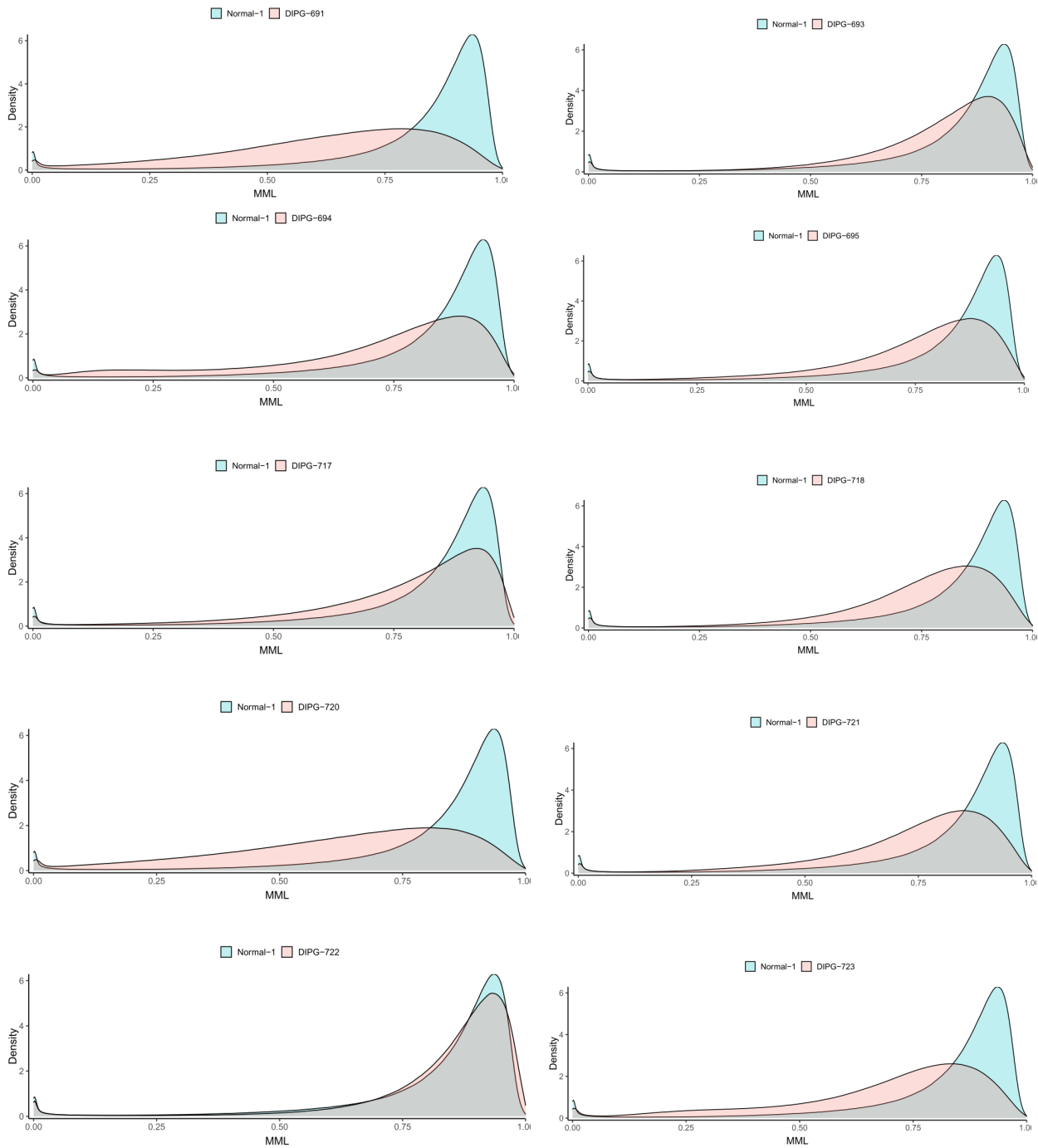

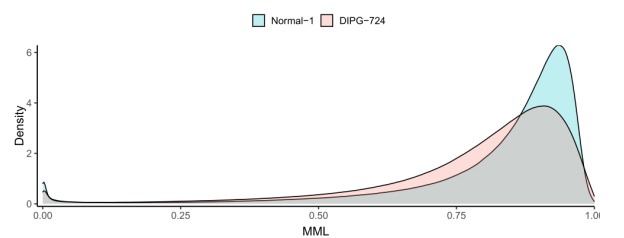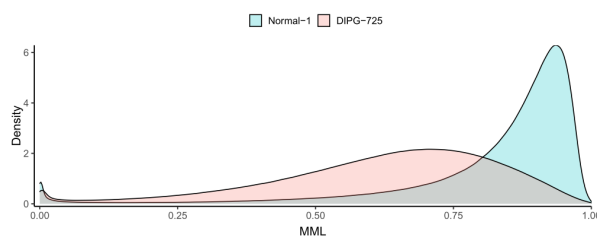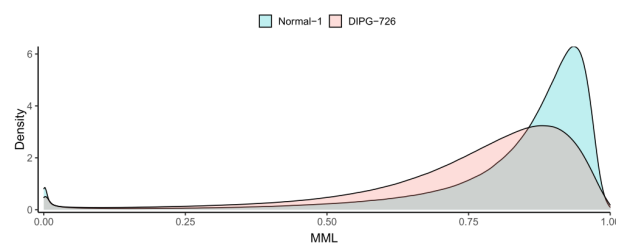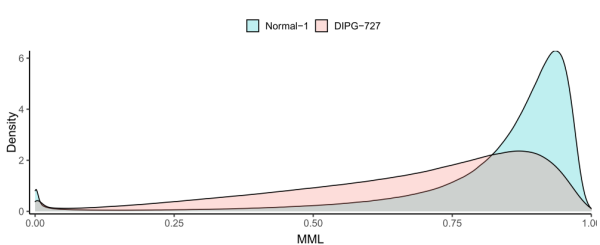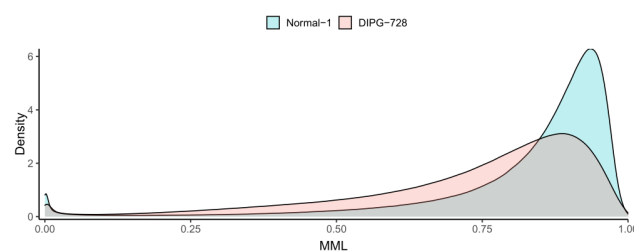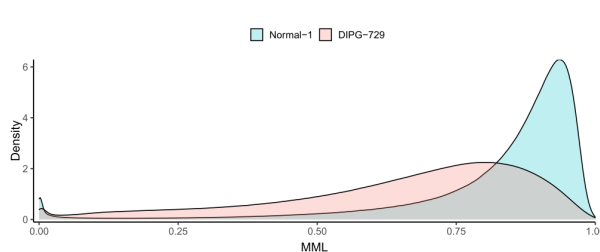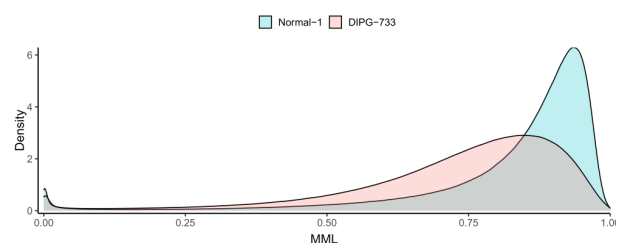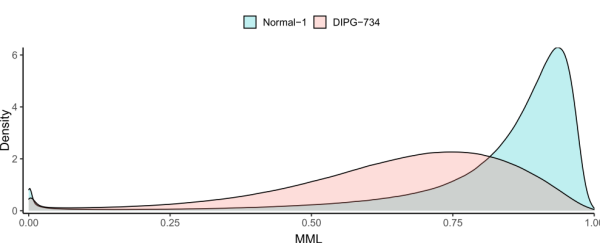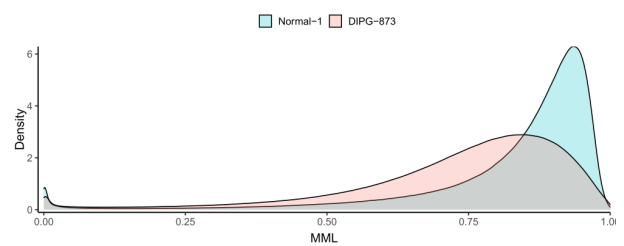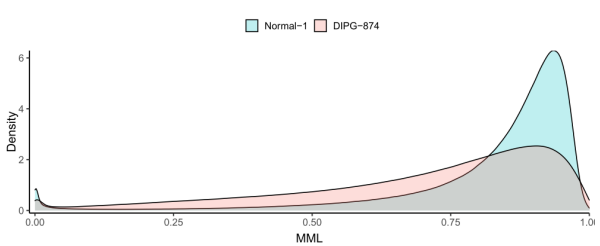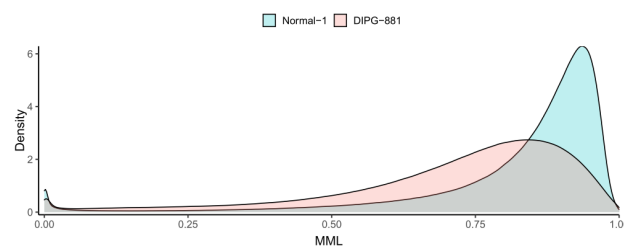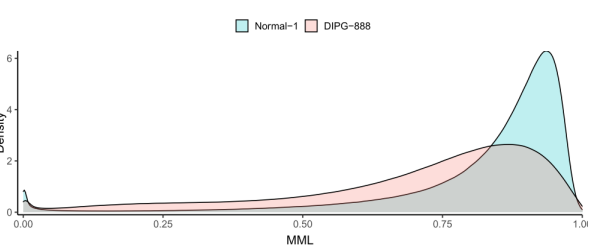

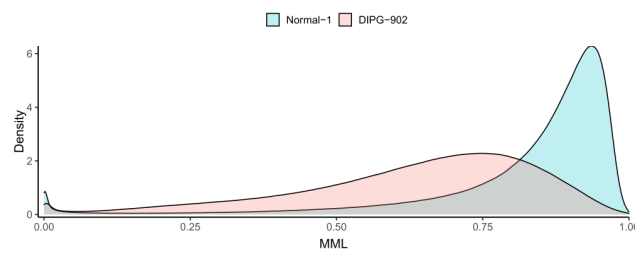

Supplementary Figure 1B

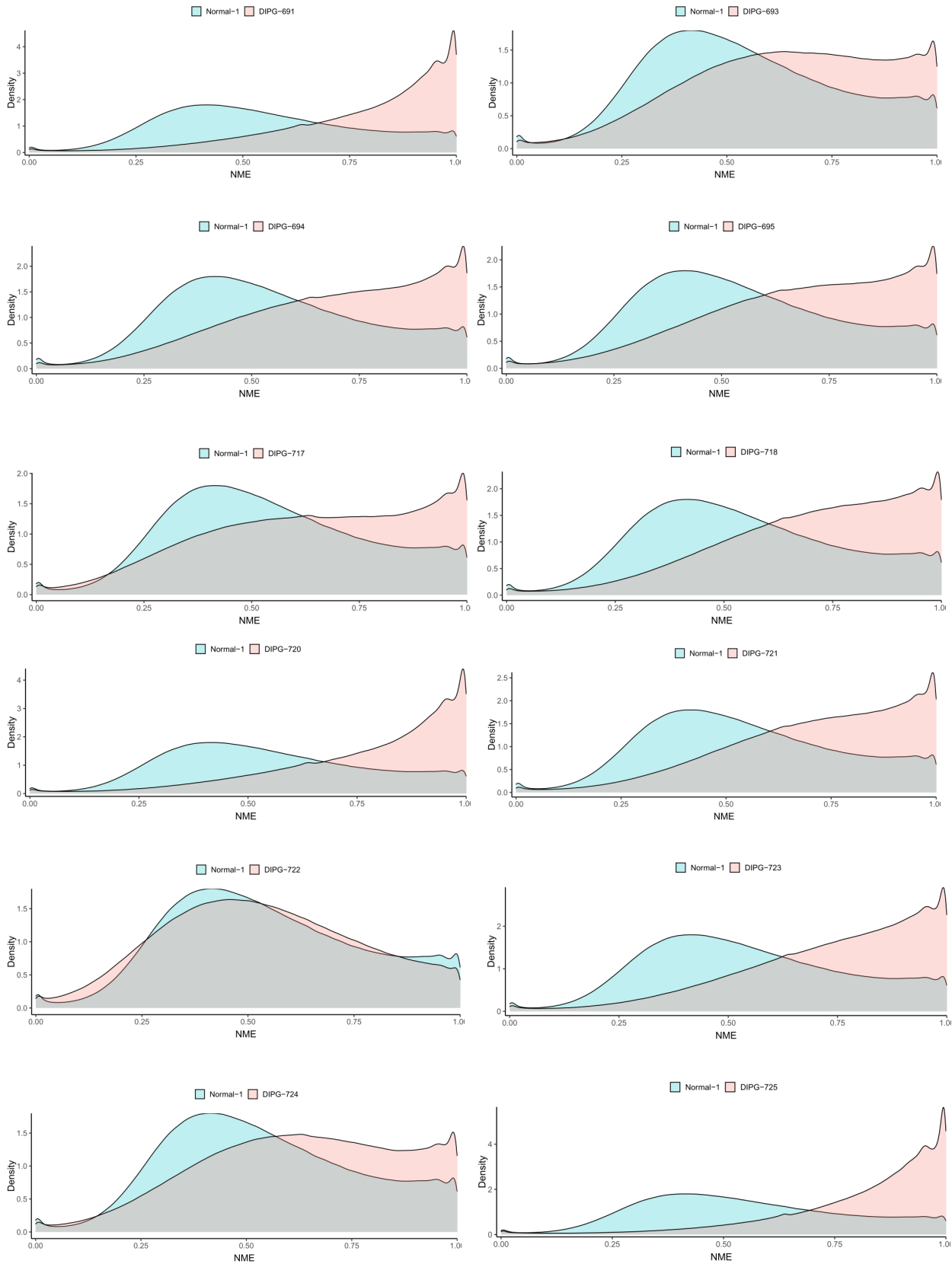

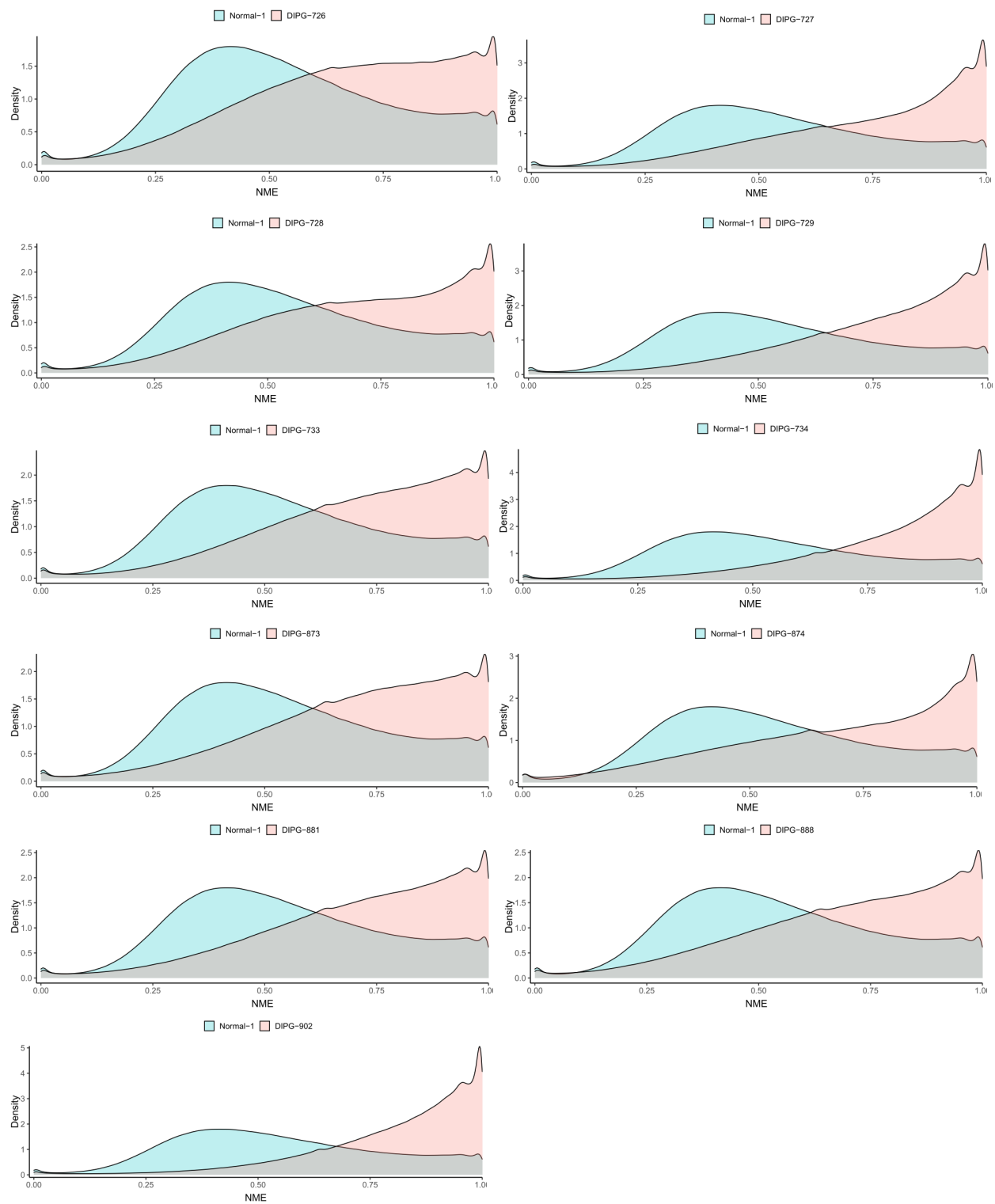

Supplementary Figure 1C

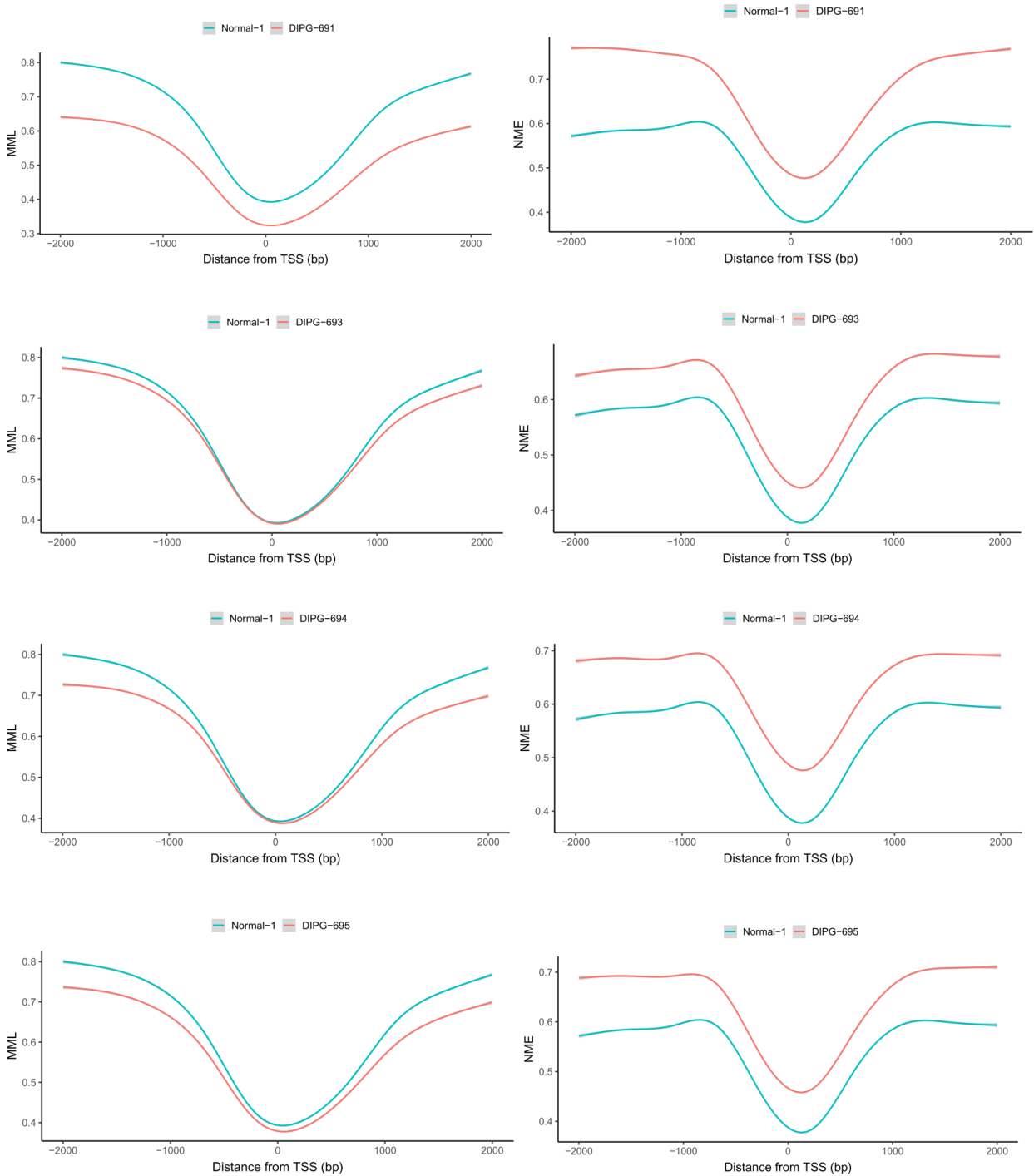

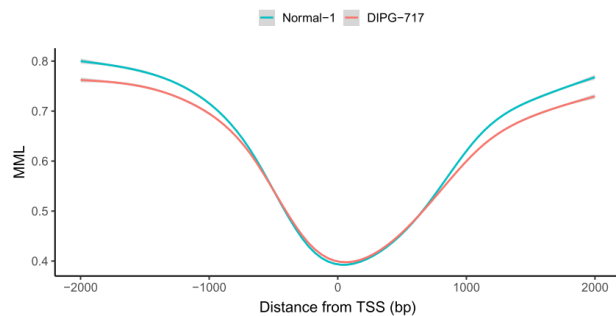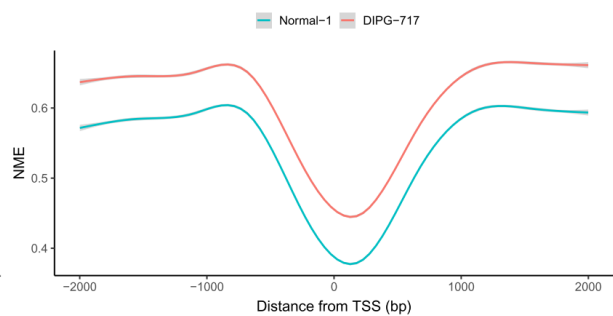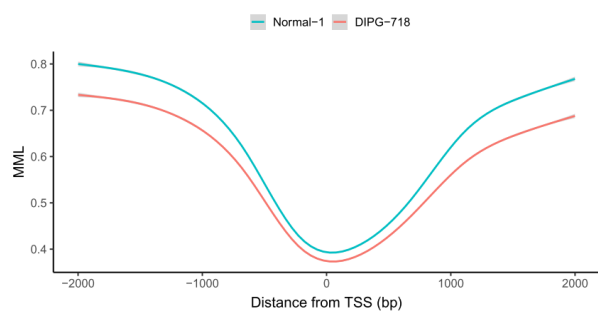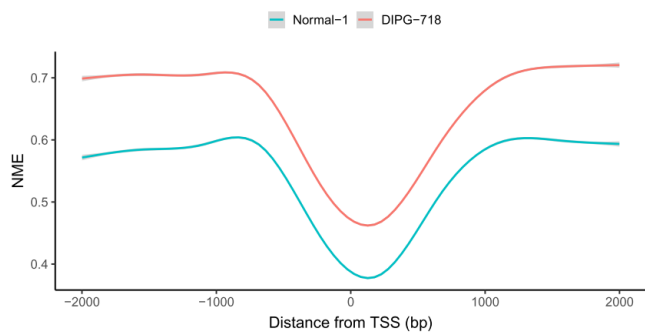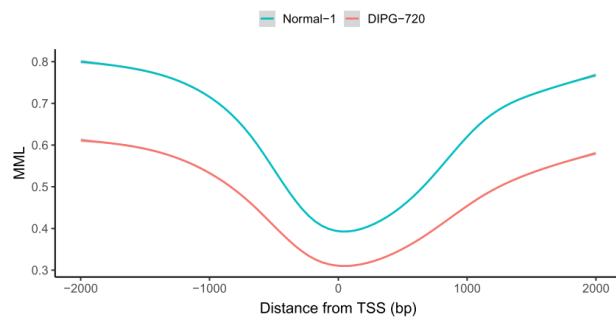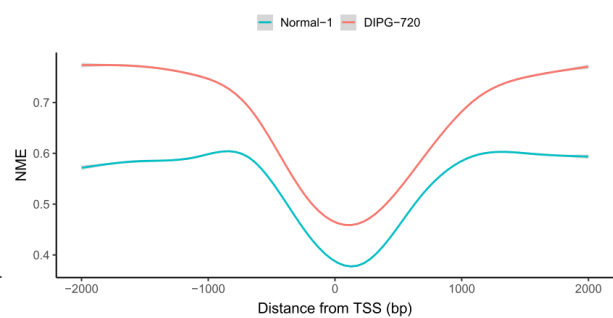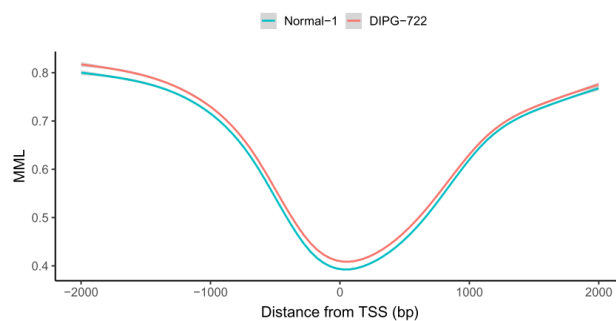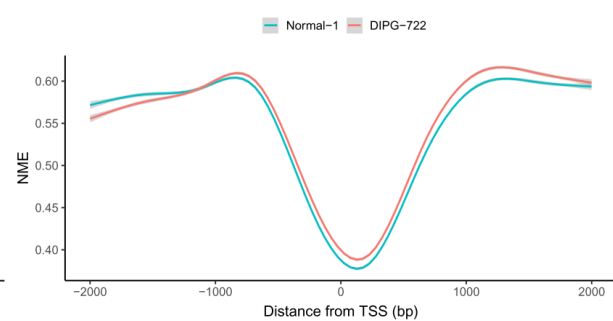

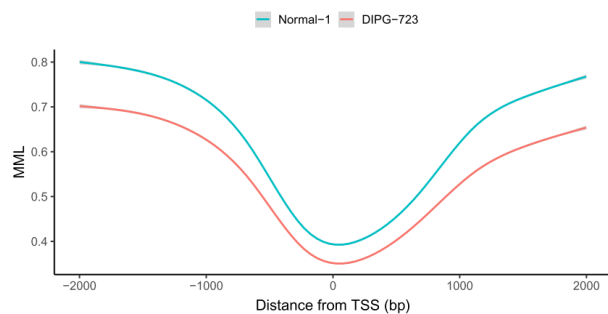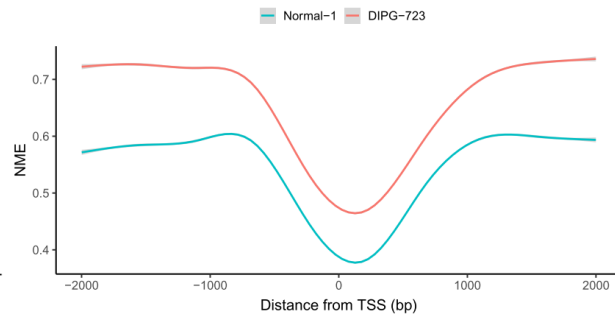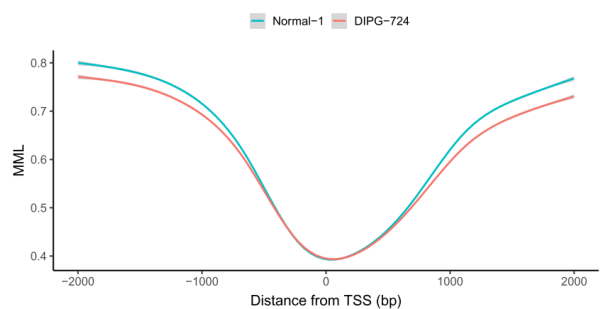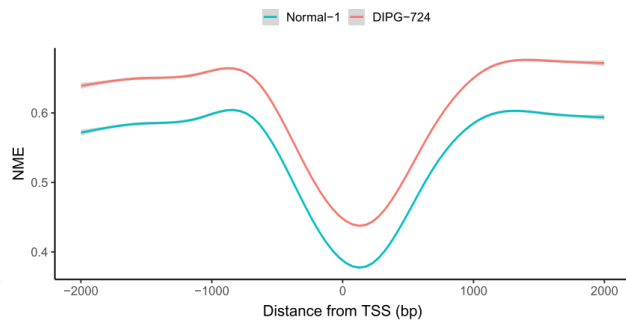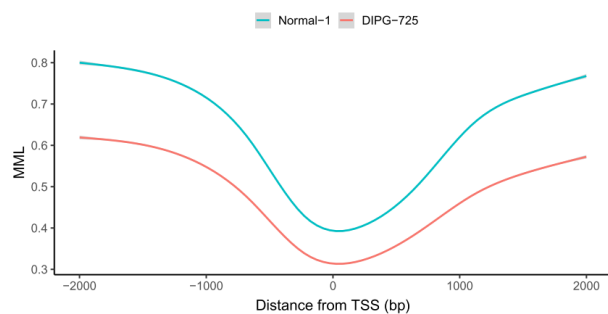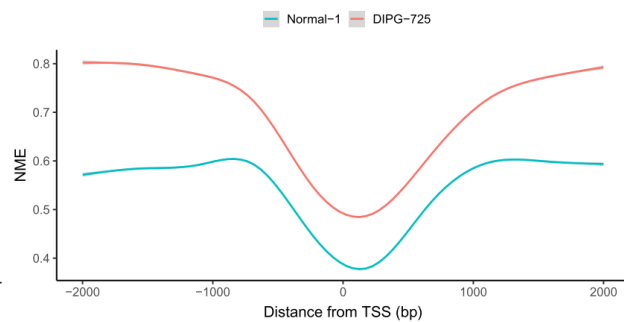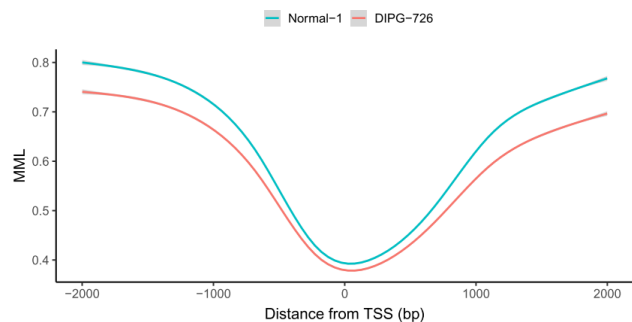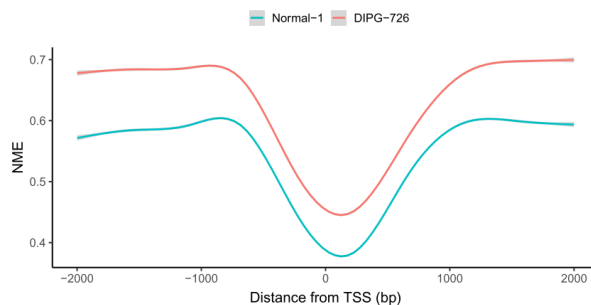



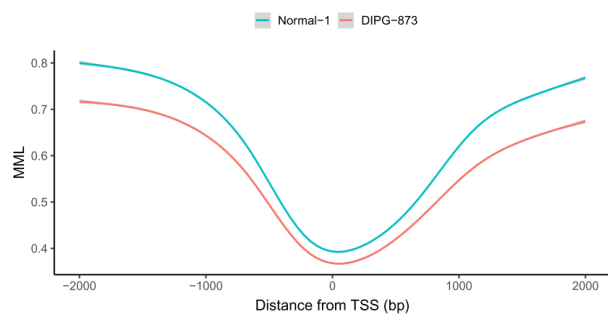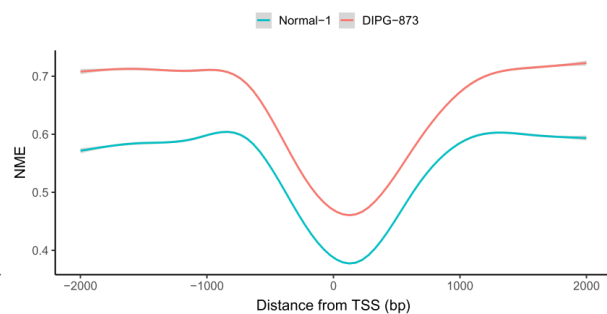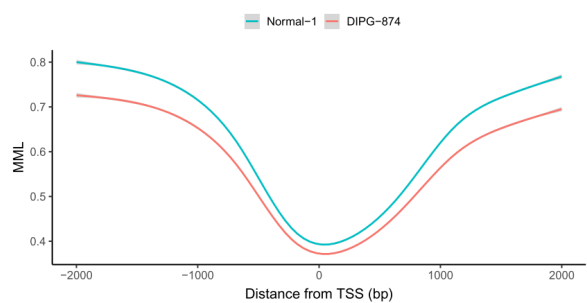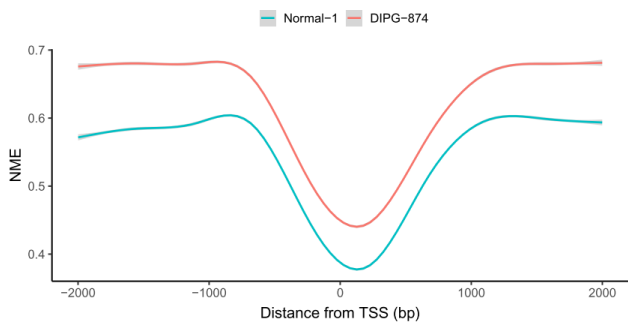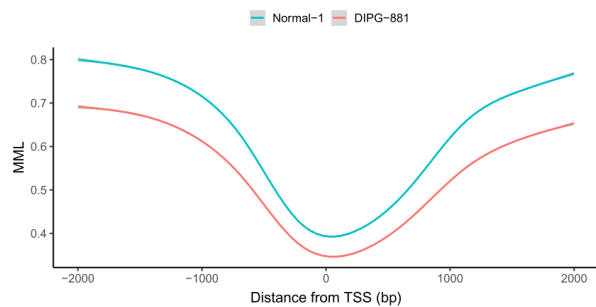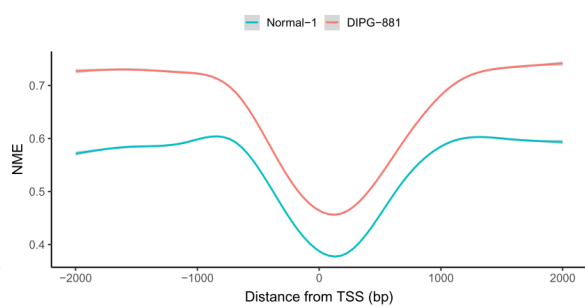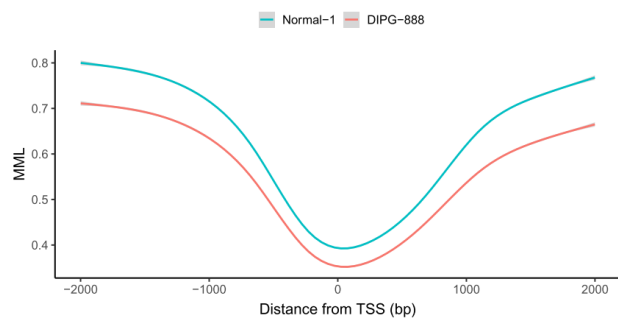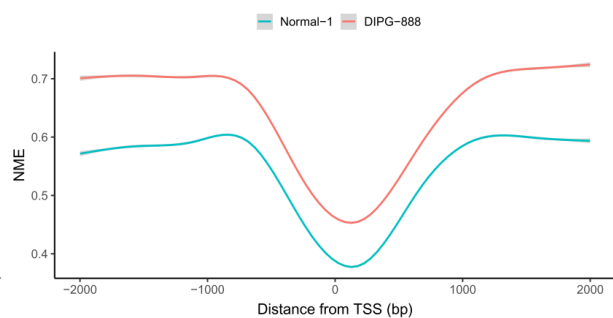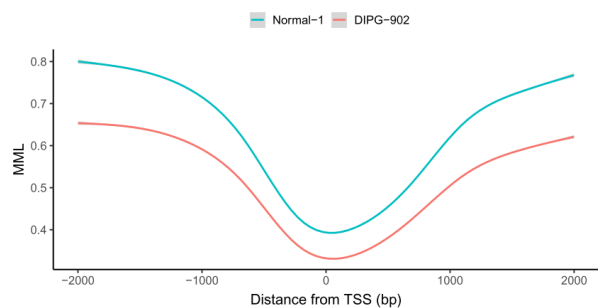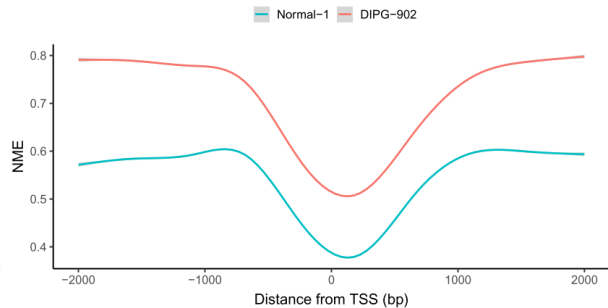

Supplement: vdae023_suppl_Supplementary_Figure_S1 [file vdae023_suppl_supplementary_figure_s1.pdf]

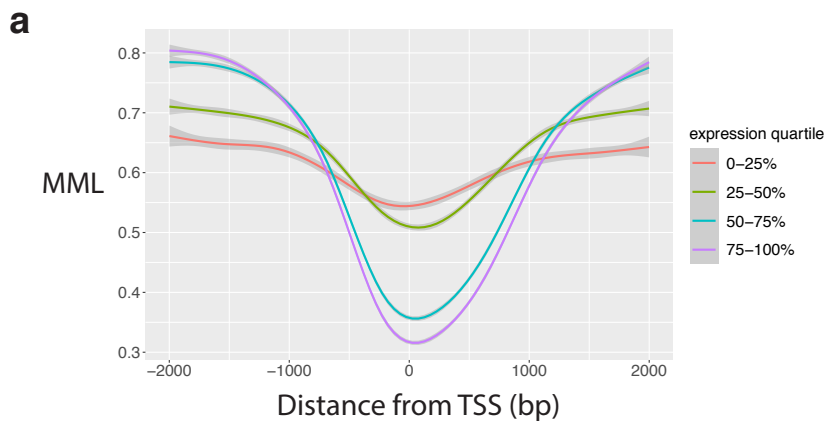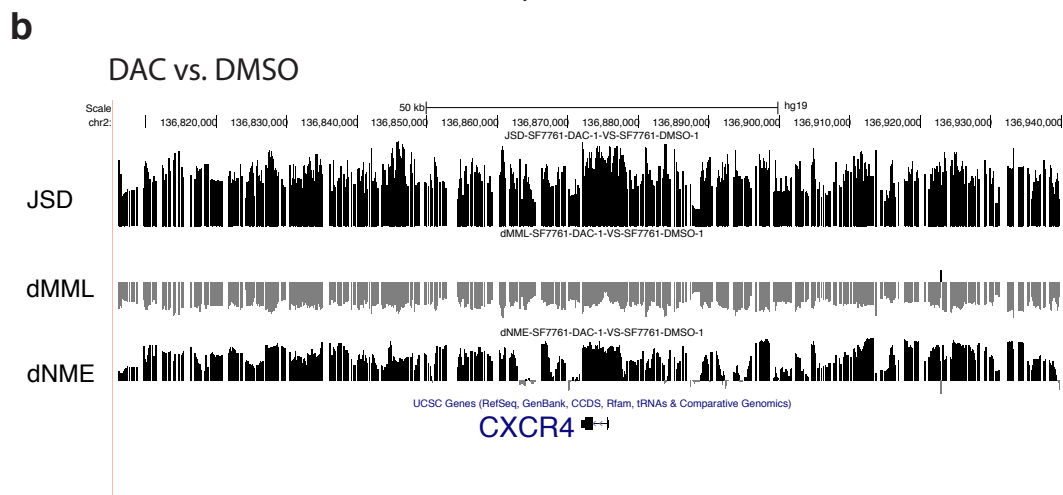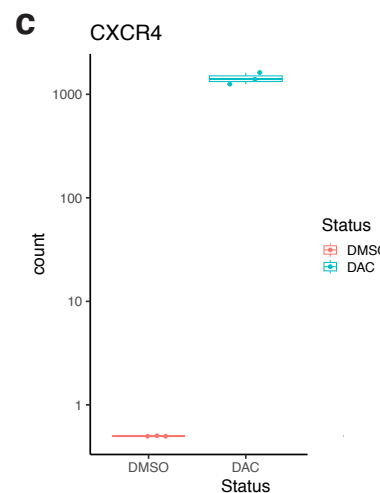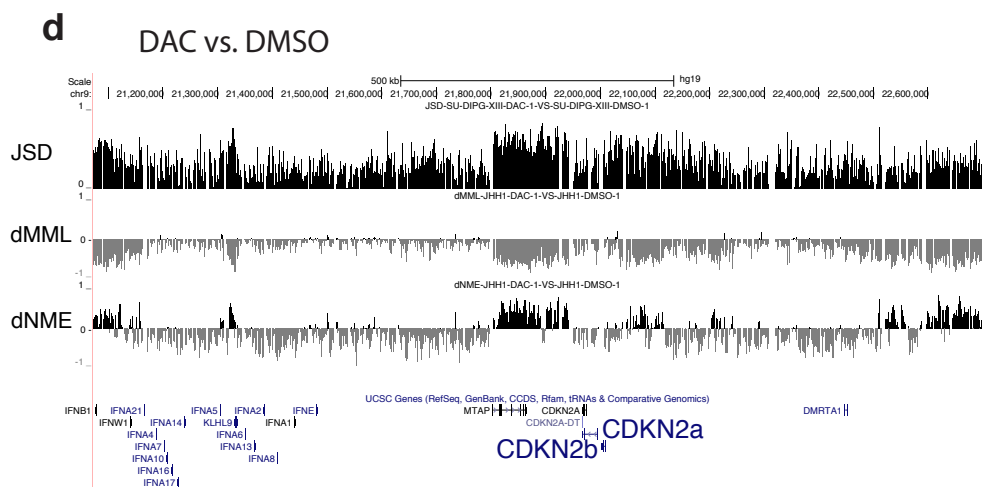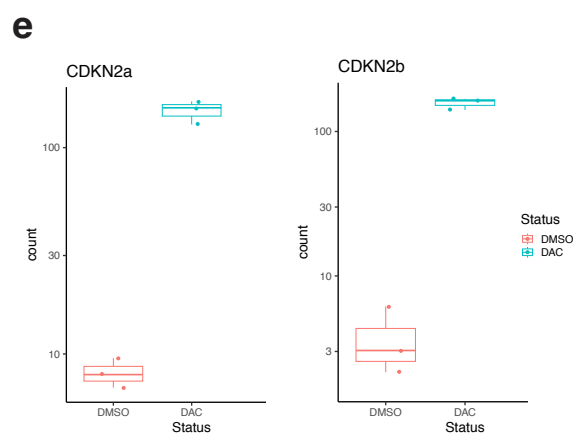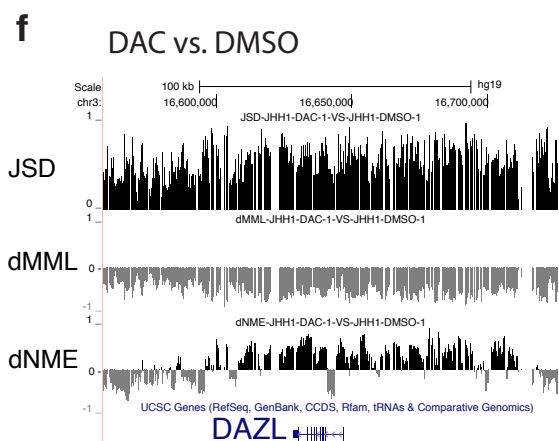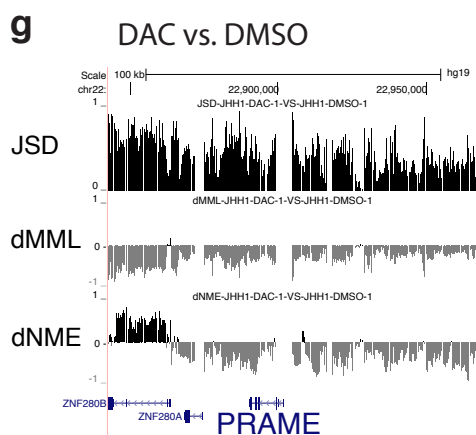

Supplement: vdae023_suppl_Supplementary_Figure_S3 [file vdae023_suppl_supplementary_figure_s3.pdf]

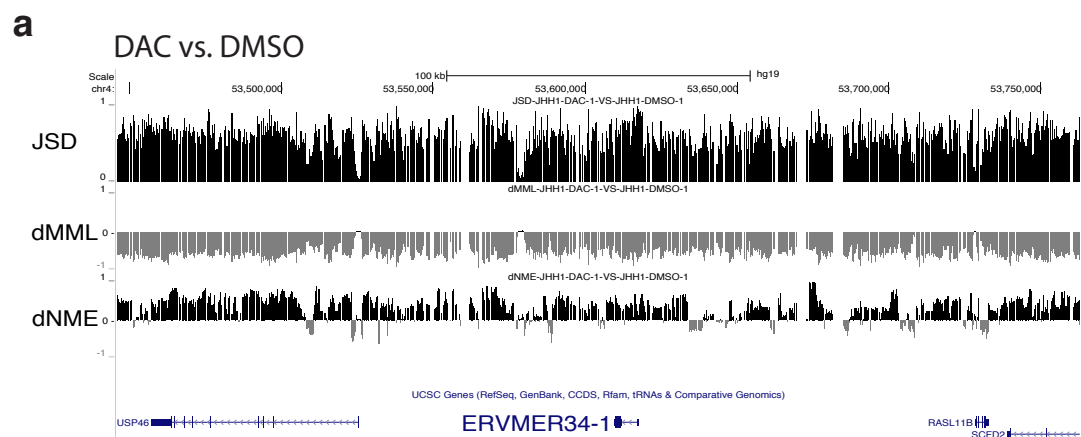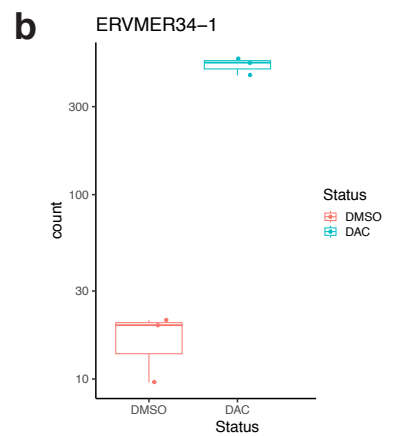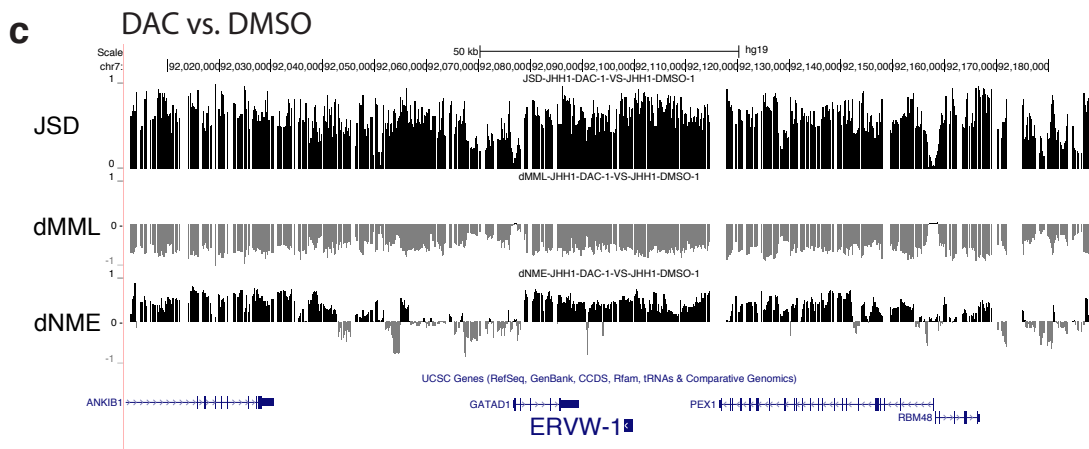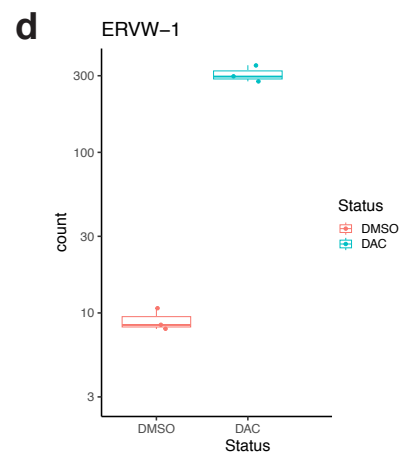

Supplement: vdae023_suppl_Supplementary_Figure_S4 [file vdae023_suppl_supplementary_figure_s4.pdf]
