# Supplementary material for: DNA methylation landscapes in DIPG reveal methylome variability that can be modified pharmacologically
Source: Neurooncol Adv. 2024 Feb 19;6(1):vdae023. doi: 10.1093/noajnl/vdae023 (PMC10926944; doi:10.1093/noajnl/vdae023)
Supplement: vdae023_suppl_Supplementary_Figure_S2 [file vdae023_suppl_supplementary_figure_s2.pdf]

Supplementary Figure 2A

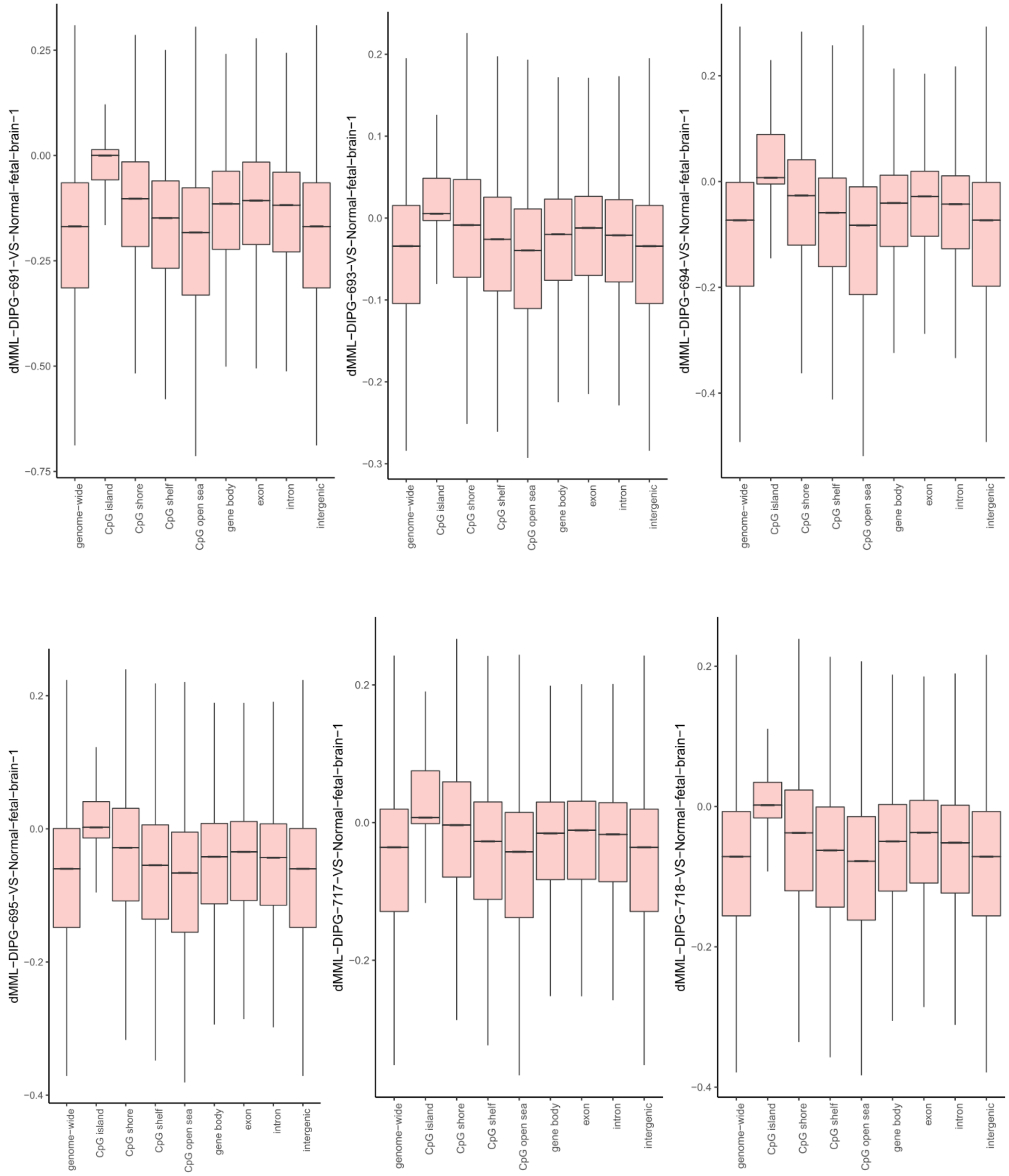

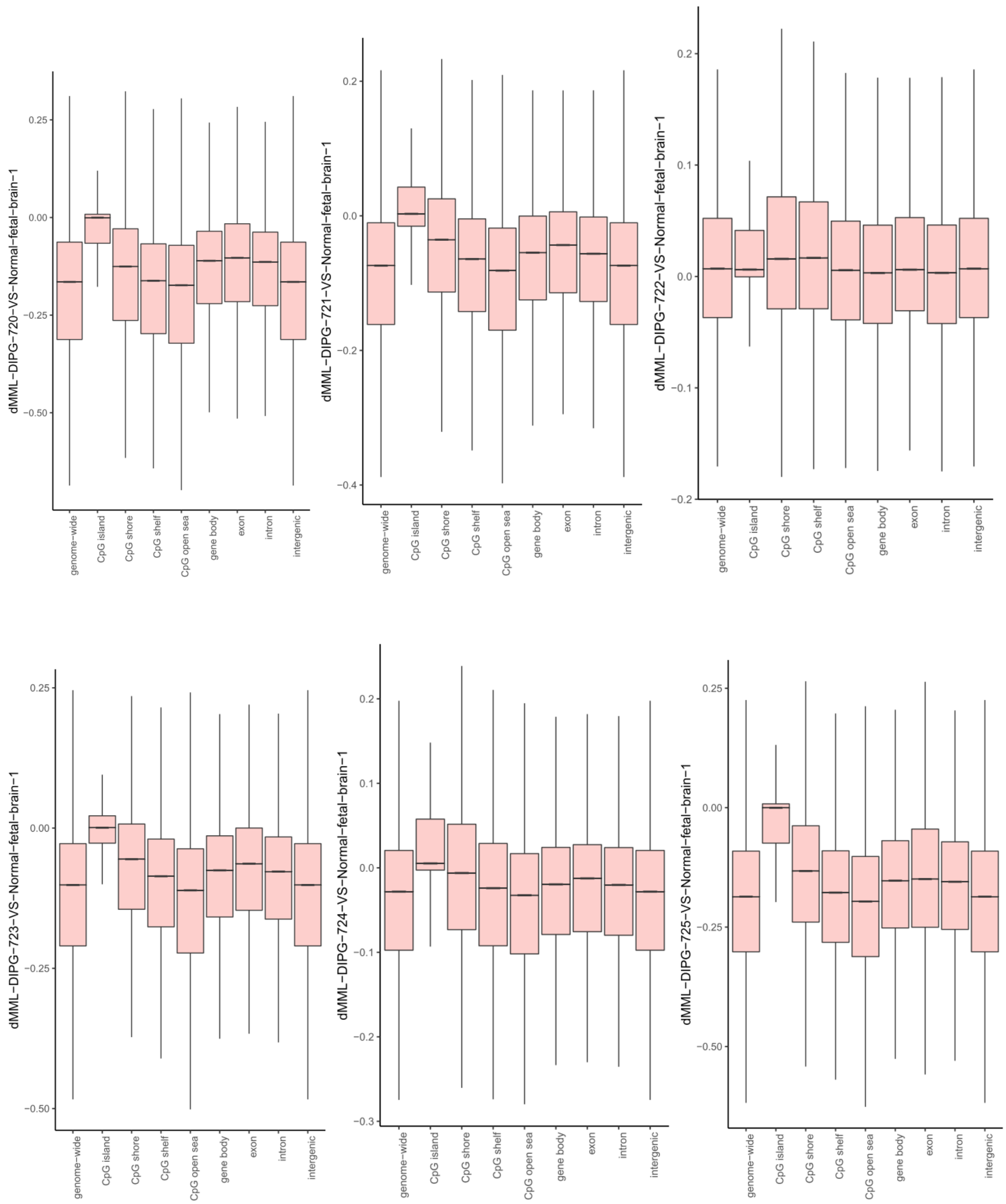
dMML−DIPG−720−VS−Normal−fetal−brain−1
0.25
0.00
−0.25
−0.50
dMML−DIPG−721−VS−Normal−fetal−brain−1
0.2
0.0
−0.2
−0.4
dMML−DIPG−722−VS−Normal−fetal−brain−1
0.2
0.1
0.0
−0.1
−0.2
dMML−DIPG−723−VS−Normal−fetal−brain−1
0.25
0.00
−0.25
−0.50
dMML−DIPG−724−VS−Normal−fetal−brain−1
0.2
0.1
0.0
−0.1
−0.2
−0.3
dMML−DIPG−725−VS−Normal−fetal−brain−1
0.25
0.00
−0.25
−0.50
genome−wide
CpG island
CpG shore
CpG shelf
CpG open sea
gene body
exon
intron
intergenic

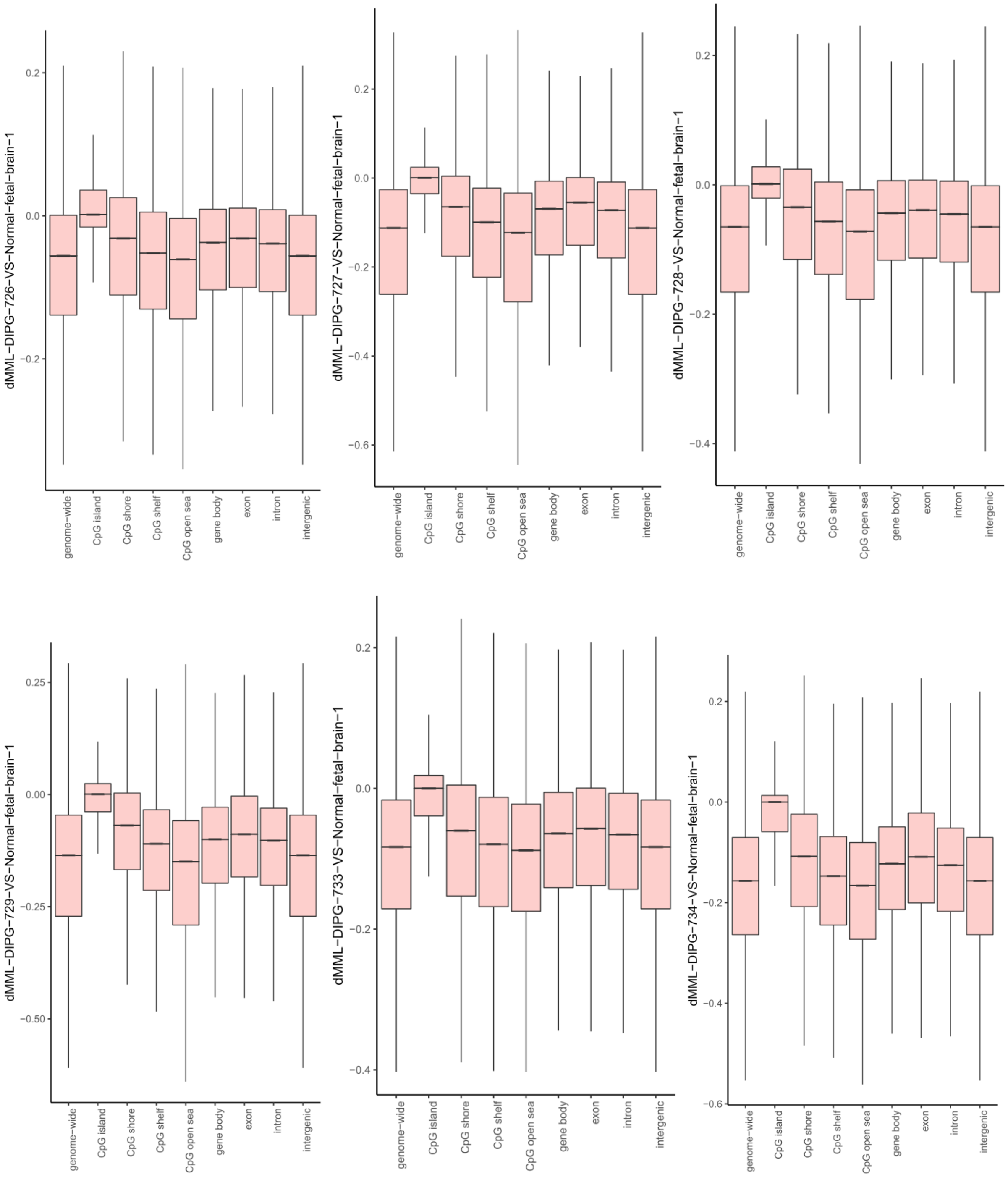

dMML−DIPG−726−VS−Normal−fetal−brain−1
0.2
0.0
−0.2
genome−wide
CpG island
CpG shore
CpG shelf
CpG open sea
gene body
exon
intron
intergenic
dMML−DIPG−727−VS−Normal−fetal−brain−1
0.2
0.0
−0.2
−0.4
−0.6
genome−wide
CpG island
CpG shore
CpG shelf
CpG open sea
gene body
exon
intron
intergenic
dMML−DIPG−728−VS−Normal−fetal−brain−1
0.2
0.0
−0.2
−0.4
genome−wide
CpG island
CpG shore
CpG shelf
CpG open sea
gene body
exon
intron
intergenic
dMML−DIPG−729−VS−Normal−fetal−brain−1
0.25
0.00
−0.25
−0.50
genome−wide
CpG island
CpG shore
CpG shelf
CpG open sea
gene body
exon
intron
intergenic
dMML−DIPG−733−VS−Normal−fetal−brain−1
0.2
0.0
−0.2
−0.4
genome−wide
CpG island
CpG shore
CpG shelf
CpG open sea
gene body
exon
intron
intergenic
dMML−DIPG−734−VS−Normal−fetal−brain−1
0.2
0.0
−0.2
−0.4
−0.6
genome−wide
CpG island
CpG shore
CpG shelf
CpG open sea
gene body
exon
intron
intergenic

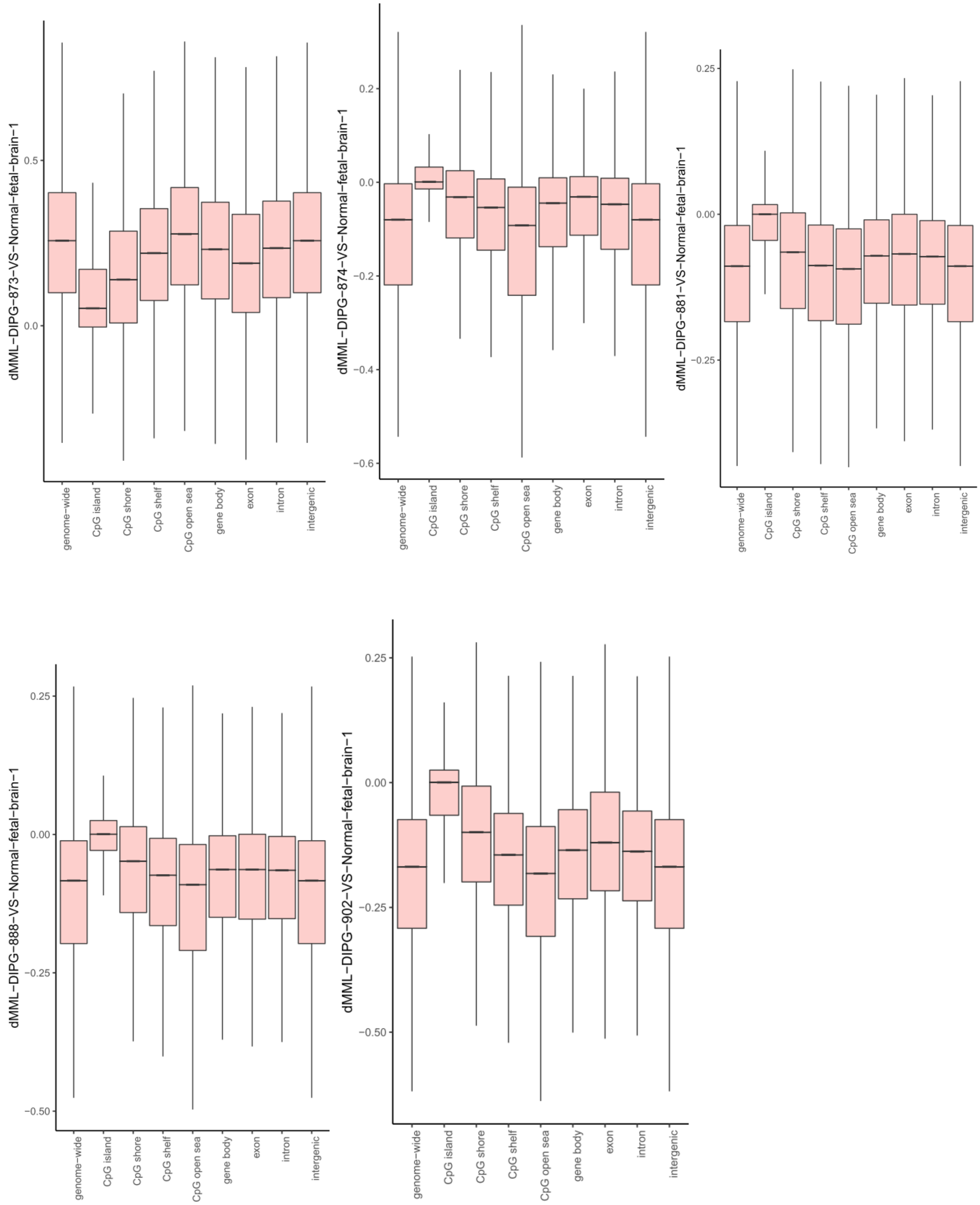
dMML-DIPG-873-VS-Normal-fetal-brain-1
0.5
0.0
dMML-DIPG-874-VS-Normal-fetal-brain-1
0.2
0.0
-0.2
-0.4
-0.6
dMML-DIPG-881-VS-Normal-fetal-brain-1
0.25
0.00
-0.25
dMML-DIPG-888-VS-Normal-fetal-brain-1
0.25
0.00
-0.25
-0.50
dMML-DIPG-902-VS-Normal-fetal-brain-1
0.25
0.00
-0.25
-0.50
genome-wide
CpG island
CpG shore
CpG shelf
CpG open sea
gene body
exon
intron
intergenic

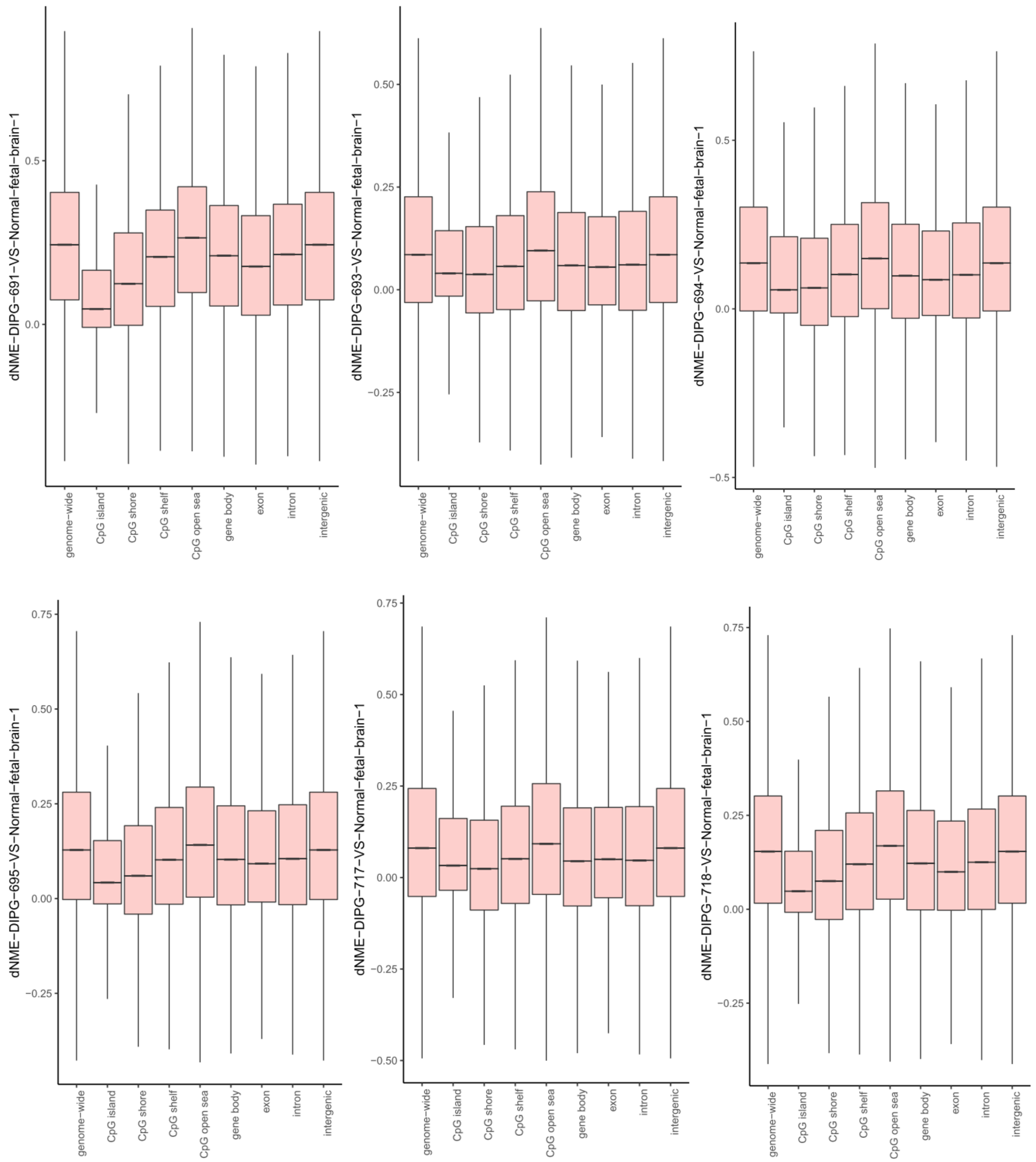

dNME−DIPG−691−VS−Normal−fetal−brain−1
0.0
0.5
dNME−DIPG−693−VS−Normal−fetal−brain−1
−0.25
0.00
0.25
0.50
dNME−DIPG−694−VS−Normal−fetal−brain−1
−0.5
0.0
0.5
dNME−DIPG−695−VS−Normal−fetal−brain−1
−0.25
0.00
0.25
0.50
0.75
dNME−DIPG−717−VS−Normal−fetal−brain−1
−0.50
−0.25
0.00
0.25
0.50
0.75
dNME−DIPG−718−VS−Normal−fetal−brain−1
−0.25
0.00
0.25
0.50
0.75
genome−wide
CpG island
CpG shore
CpG shelf
CpG open sea
gene body
exon
intron
intergenic

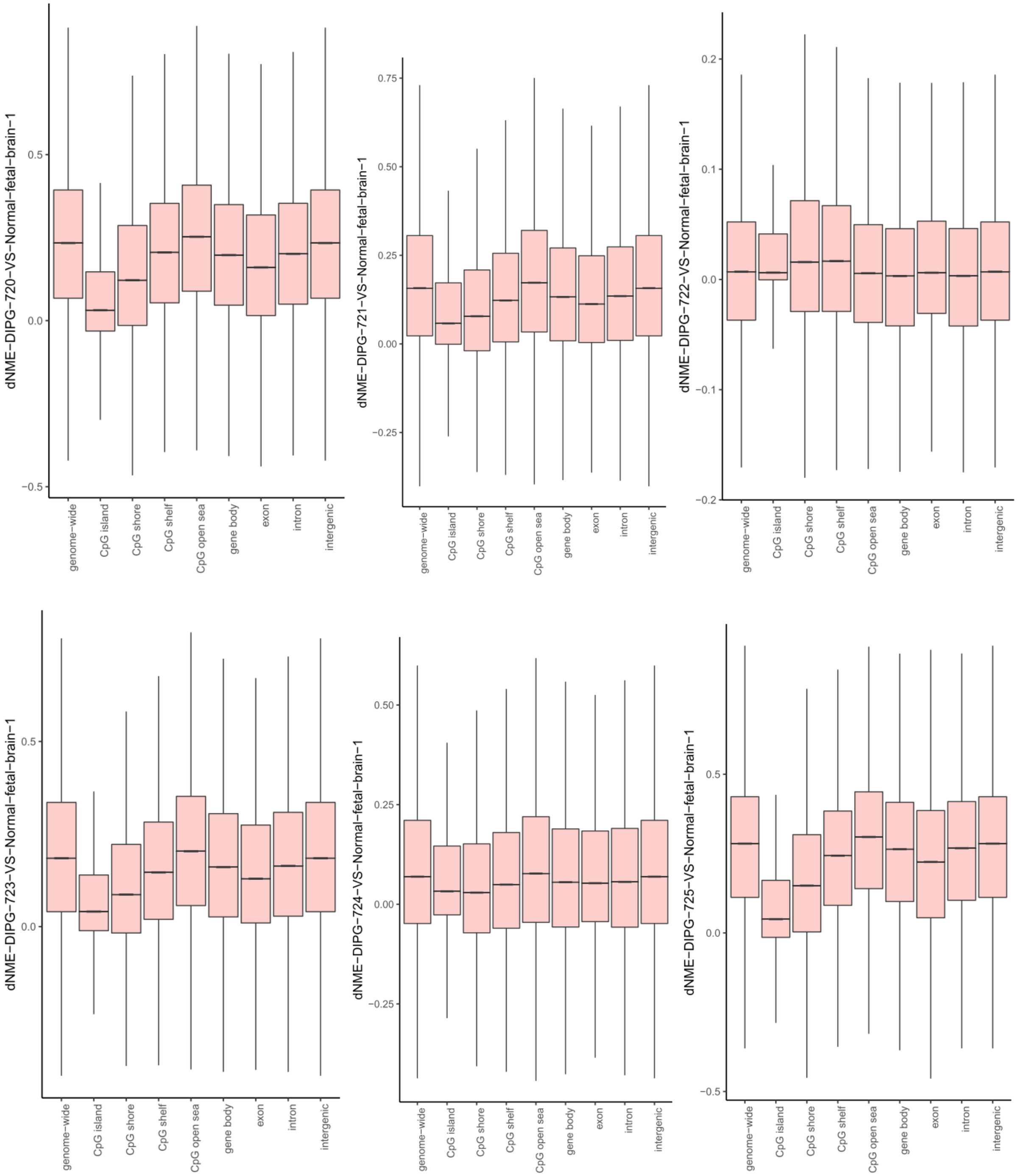
dNME−DIPG−720−VS−Normal−fetal−brain−1
0.5
0.0
−0.5
dNME−DIPG−721−VS−Normal−fetal−brain−1
0.75
0.50
0.25
0.00
−0.25
dNME−DIPG−722−VS−Normal−fetal−brain−1
0.2
0.1
0.0
−0.1
−0.2
dNME−DIPG−723−VS−Normal−fetal−brain−1
0.5
0.0
dNME−DIPG−724−VS−Normal−fetal−brain−1
0.50
0.25
0.00
−0.25
dNME−DIPG−725−VS−Normal−fetal−brain−1
0.5
0.0
−0.5
genome−wide
CpG island
CpG shore
CpG shelf
CpG open sea
gene body
exon
intron
intergenic

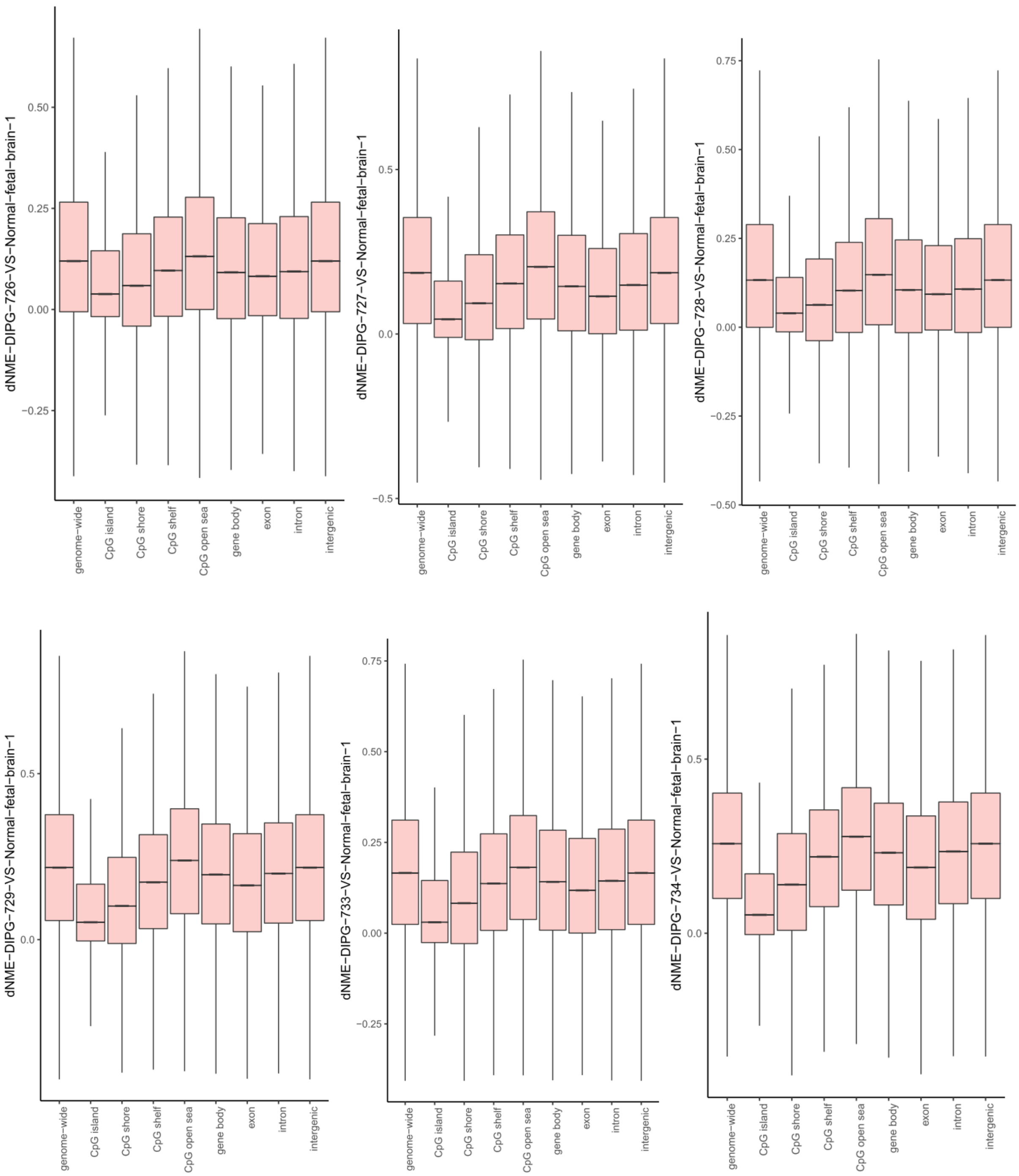

dNME−DIPG−726−VS−Normal−fetal−brain−1
0.50
0.25
0.00
−0.25
genome−wide
CpG island
CpG shore
CpG shelf
CpG open sea
gene body
exon
intron
intergenic
dNME−DIPG−727−VS−Normal−fetal−brain−1
0.5
0.0
−0.5
dNME−DIPG−728−VS−Normal−fetal−brain−1
0.75
0.50
0.25
0.00
−0.25
−0.50
dNME−DIPG−729−VS−Normal−fetal−brain−1
0.5
0.0
dNME−DIPG−733−VS−Normal−fetal−brain−1
0.75
0.50
0.25
0.00
−0.25
dNME−DIPG−734−VS−Normal−fetal−brain−1
0.5
0.0

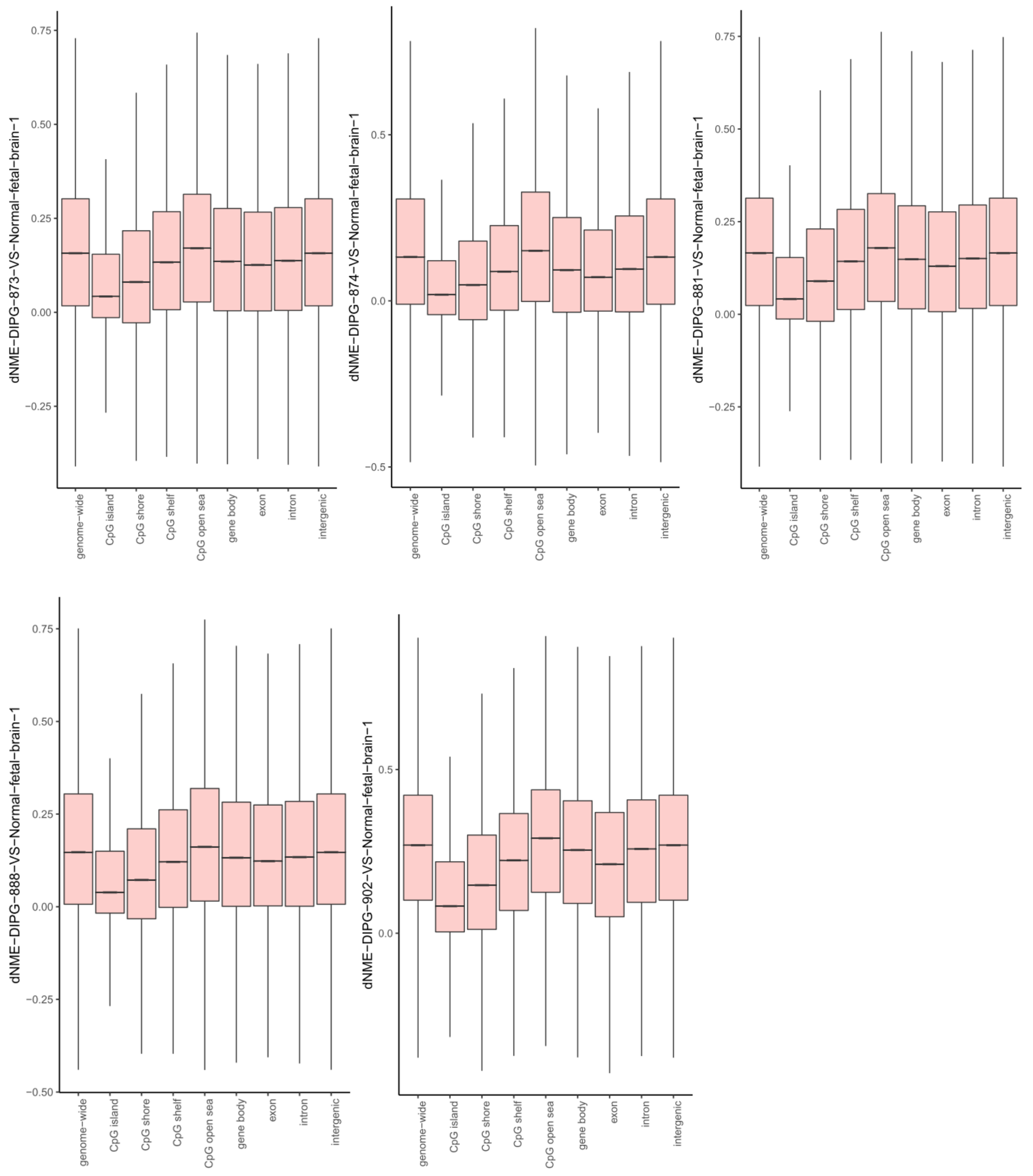
dNME-DIPG-873-VS-Normal-fetal-brain-1
dNME-DIPG-874-VS-Normal-fetal-brain-1
dNME-DIPG-881-VS-Normal-fetal-brain-1
dNME-DIPG-888-VS-Normal-fetal-brain-1
dNME-DIPG-902-VS-Normal-fetal-brain-1
0.75
0.50
0.25
0.00
-0.25
-0.50
0.5
0.0
-0.5
genome-wide
CpG island
CpG shore
CpG shelf
CpG open sea
gene body
exon
intron
intergenic

Supplementary Figure 2B

DIPG-691

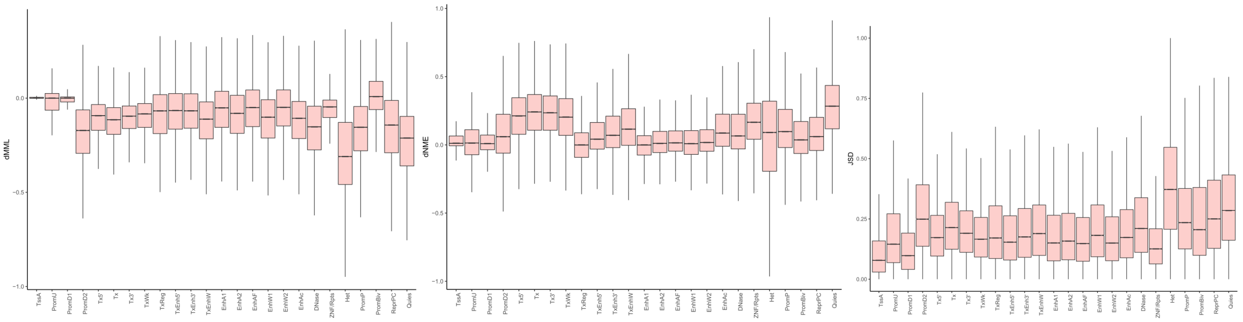

DIPG-693

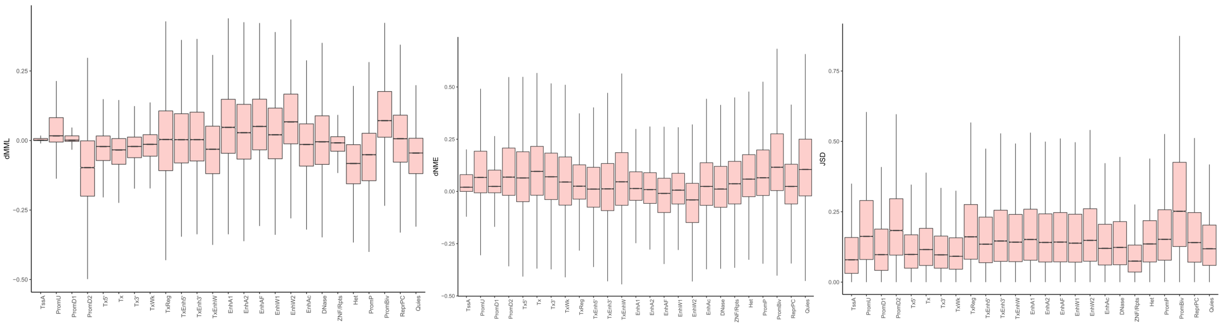

DIPG-694

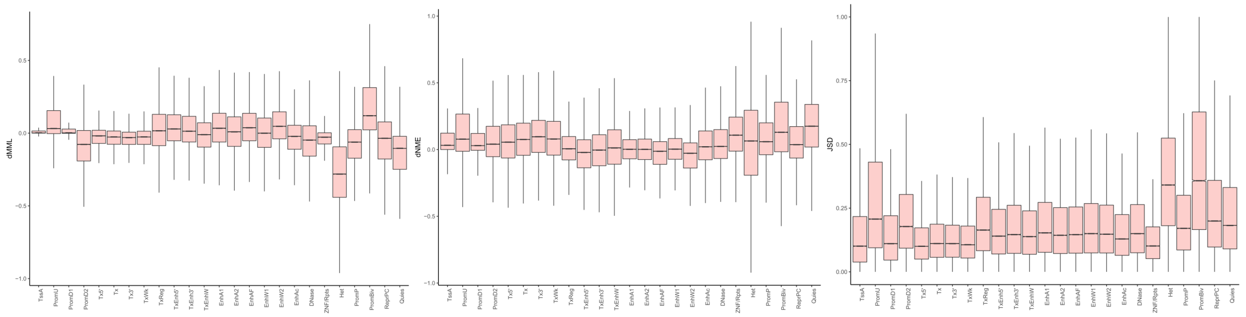

DIPG-695

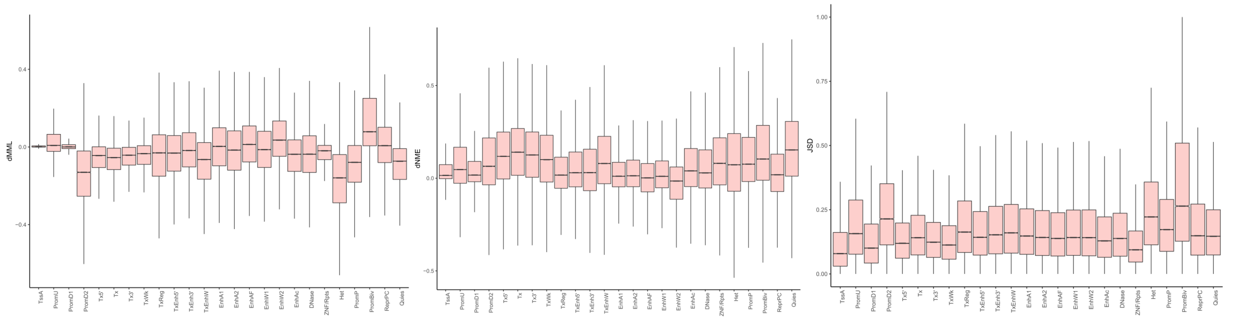

DIPG-717

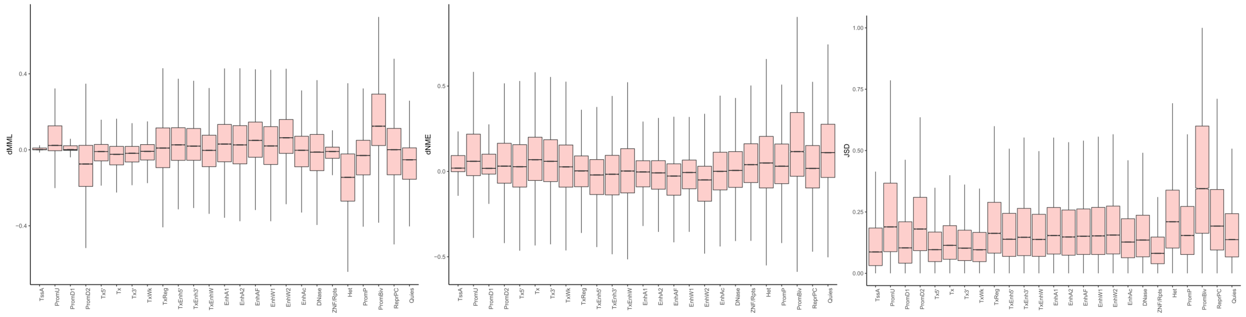

DIPG-718

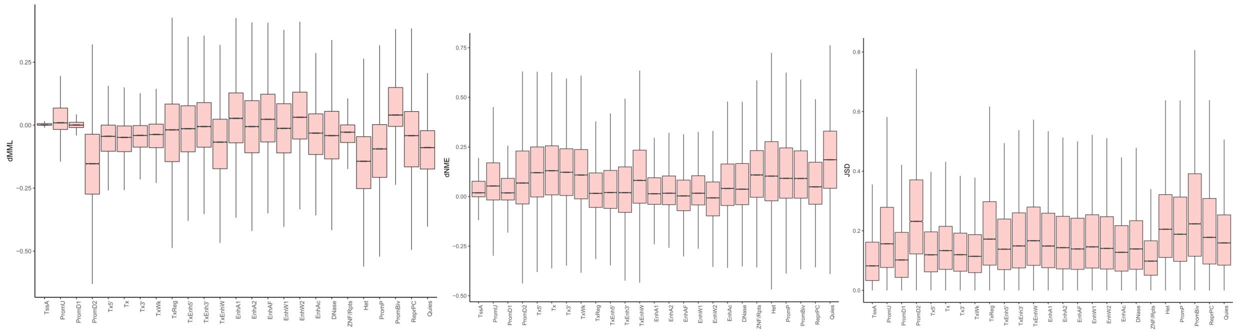

DIPG-720

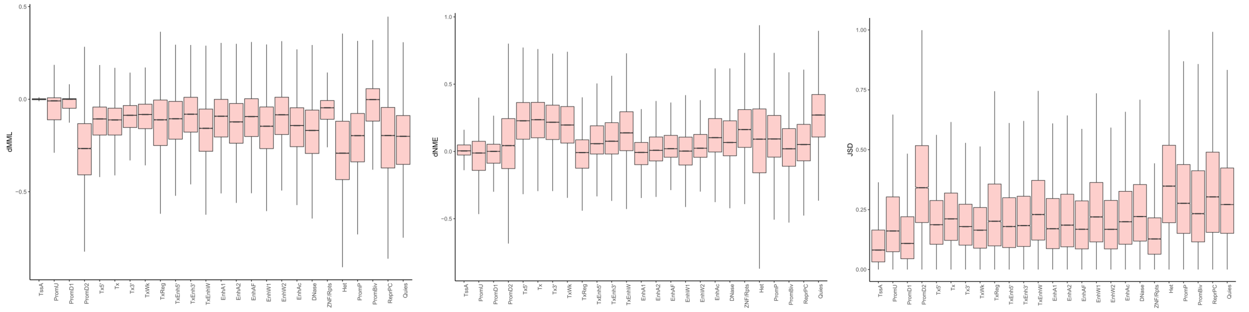

DIPG-721

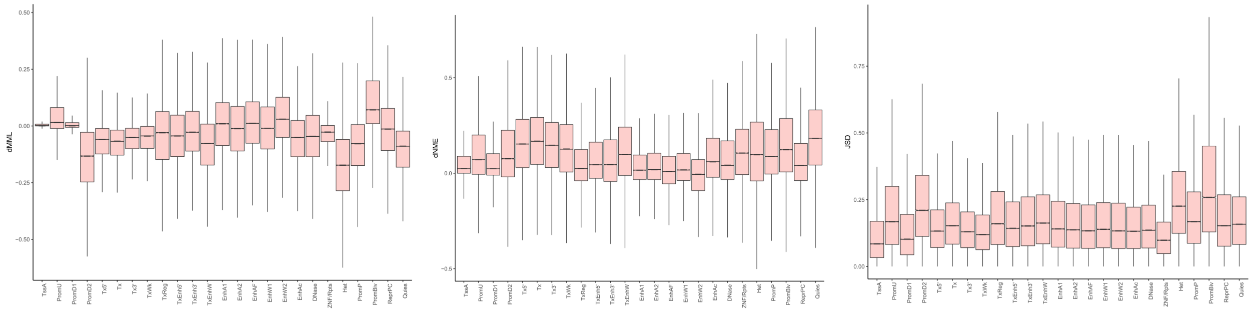

DIPG-722

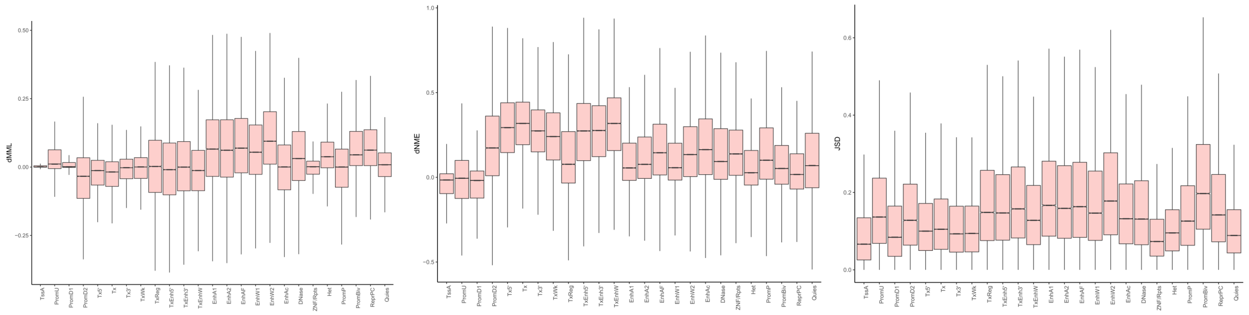

DIPG-723

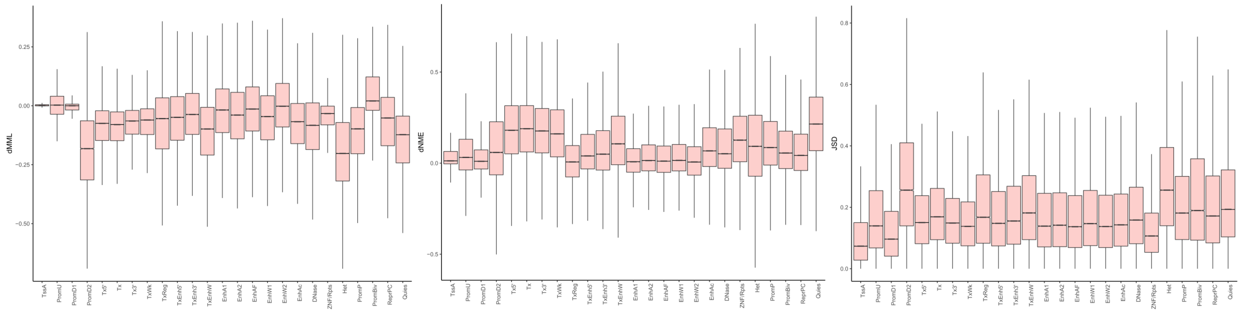

DIPG-724

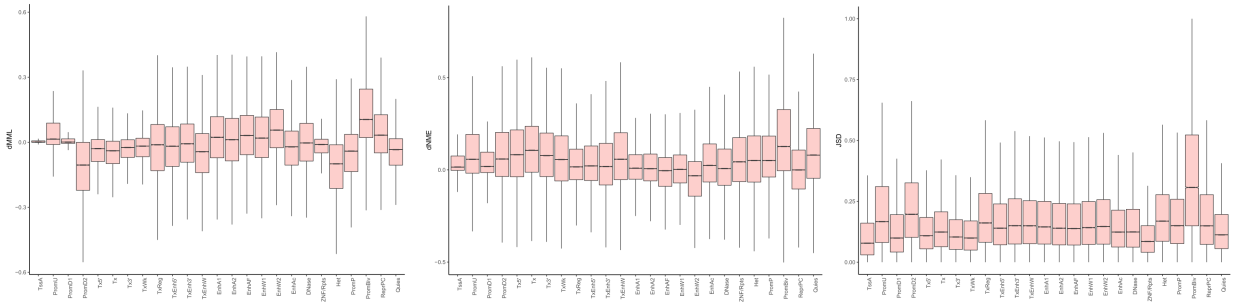

DIPG-725

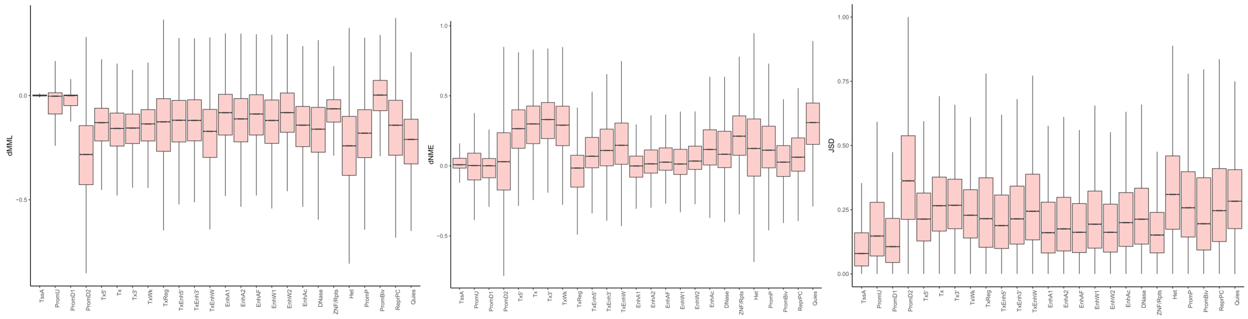

DIPG-726

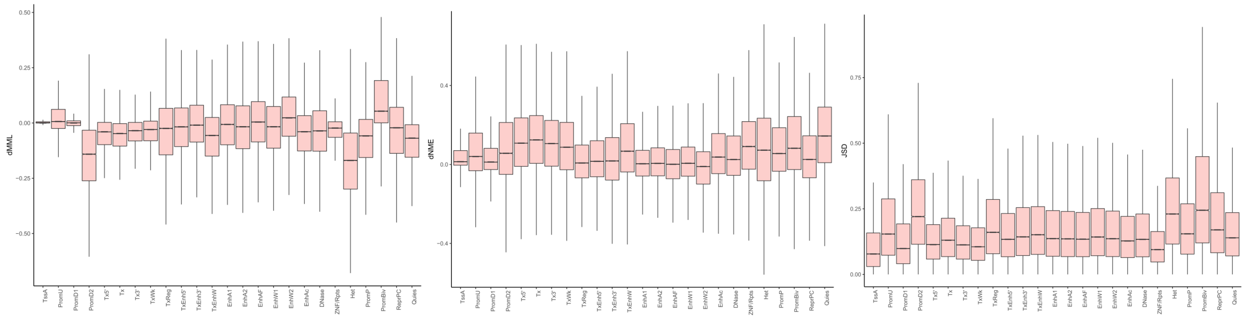

DIPG-727

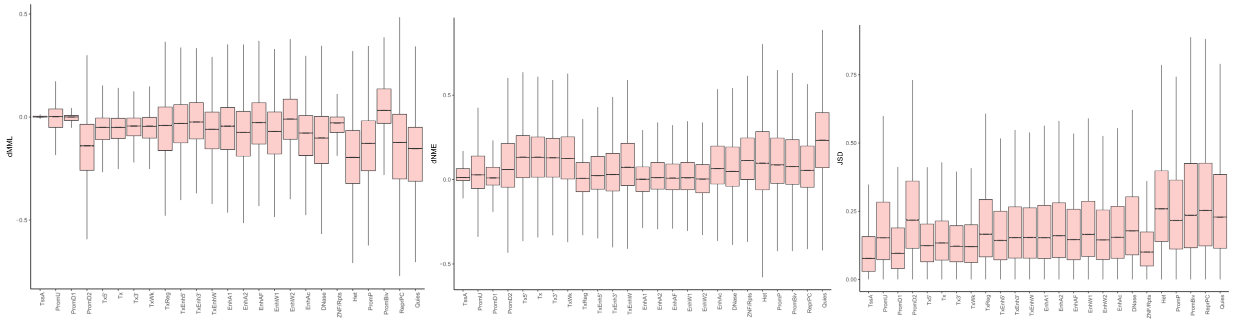

DIPG-728

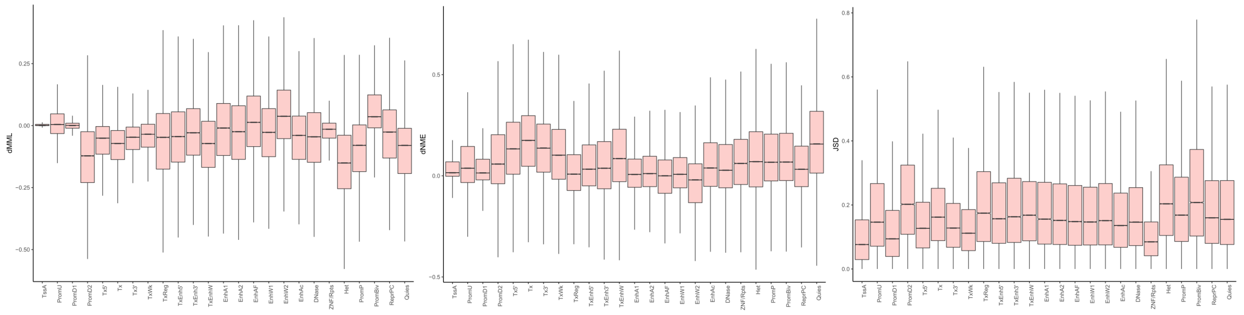

DIPG-729

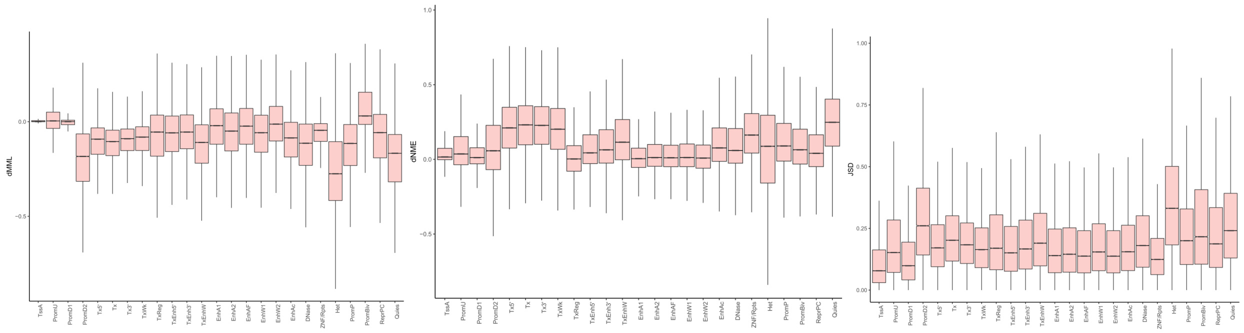

DIPG-733

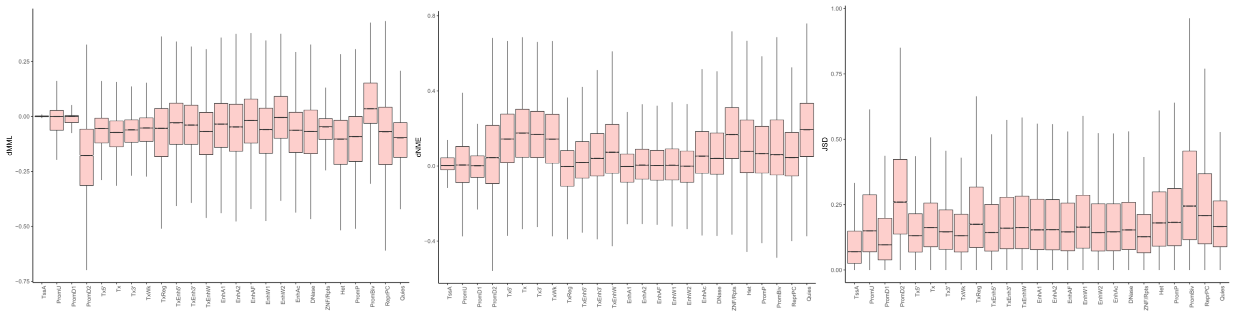

DIPG-734

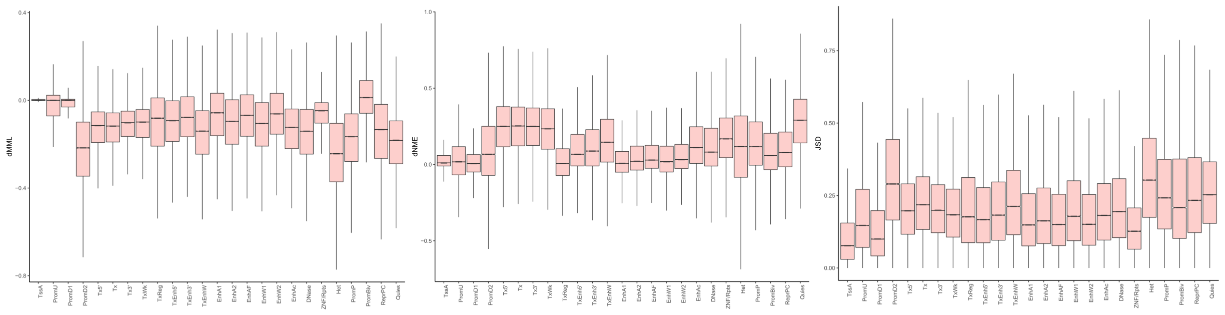

DIPG-873

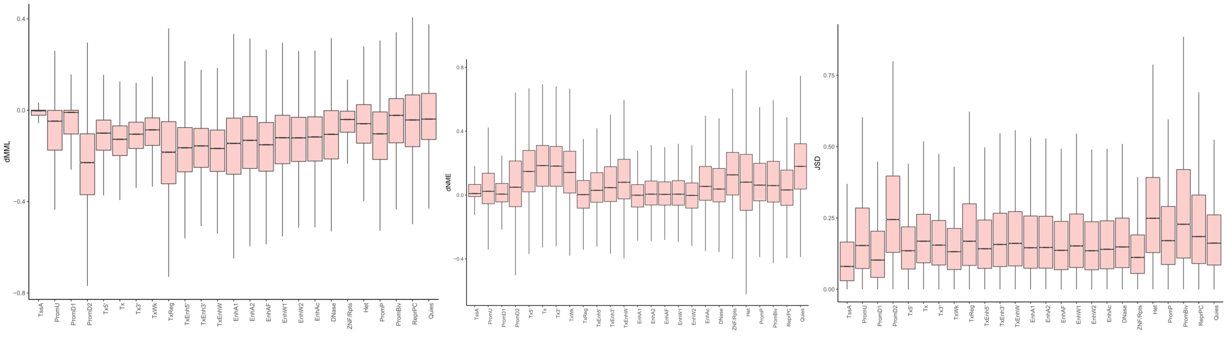

DIPG-874

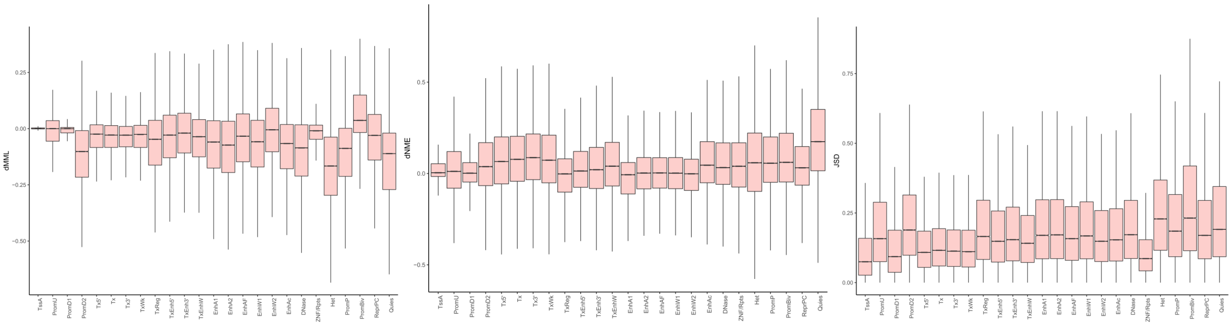

DIPG-881

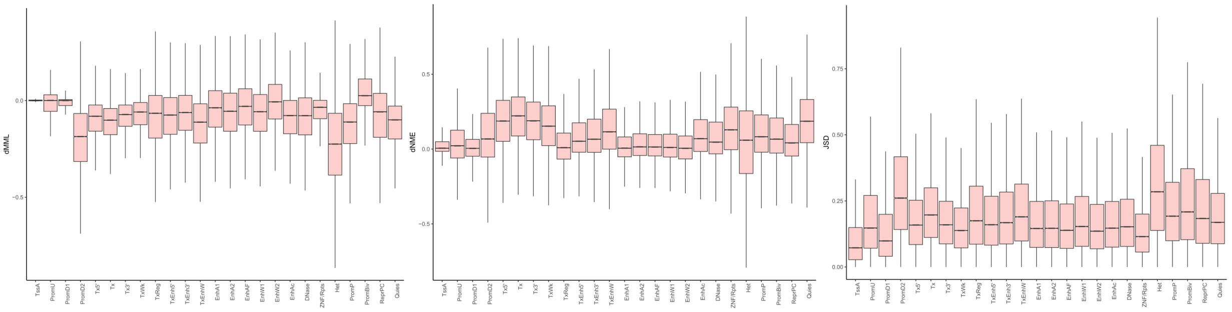

DIPG-888

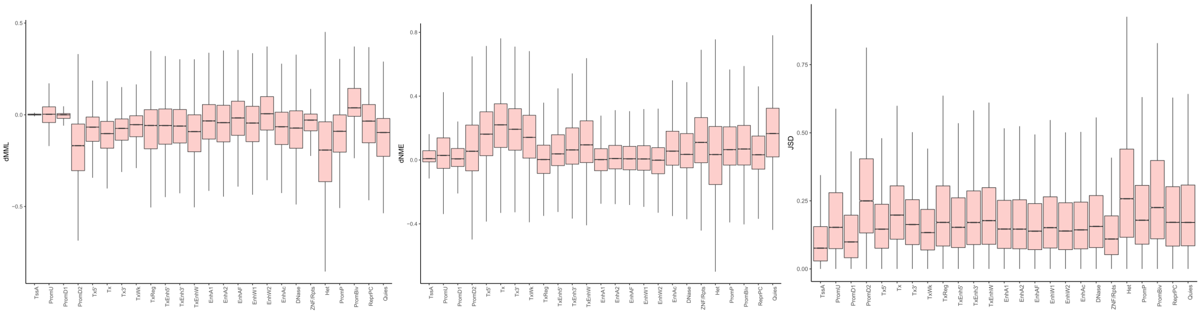

DIPG-902

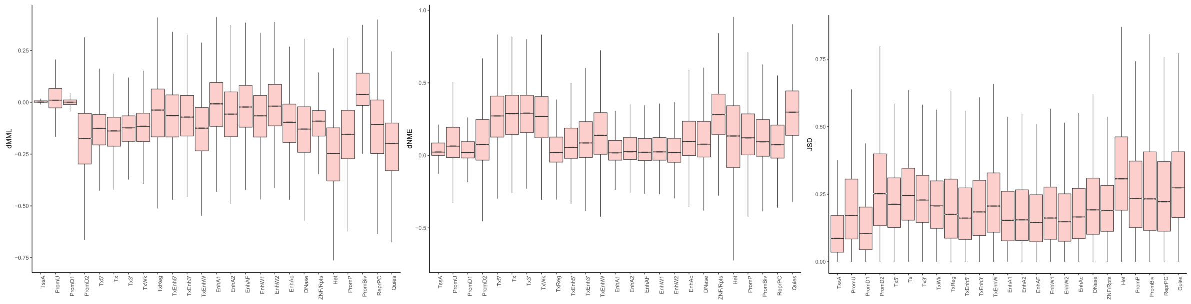
dMML
0.25
0.00
−0.25
−0.50
−0.75
TssA
PromU
PromD1
PromD2
Tx5'
Tx
Tx3'
TxWk
TxReg
TxEnh5'
TxEnh3'
TxEnhW
EnhA1
EnhA2
EnhAF
EnhW1
EnhW2
EnhAc
DNase
ZNF/Rpts
Het
PromP
PromBiv
ReprPC
Quies
dNME
1.0
0.5
0.0
−0.5
JSD
0.75
0.50
0.25
0.00

Supplementary Figure 2C

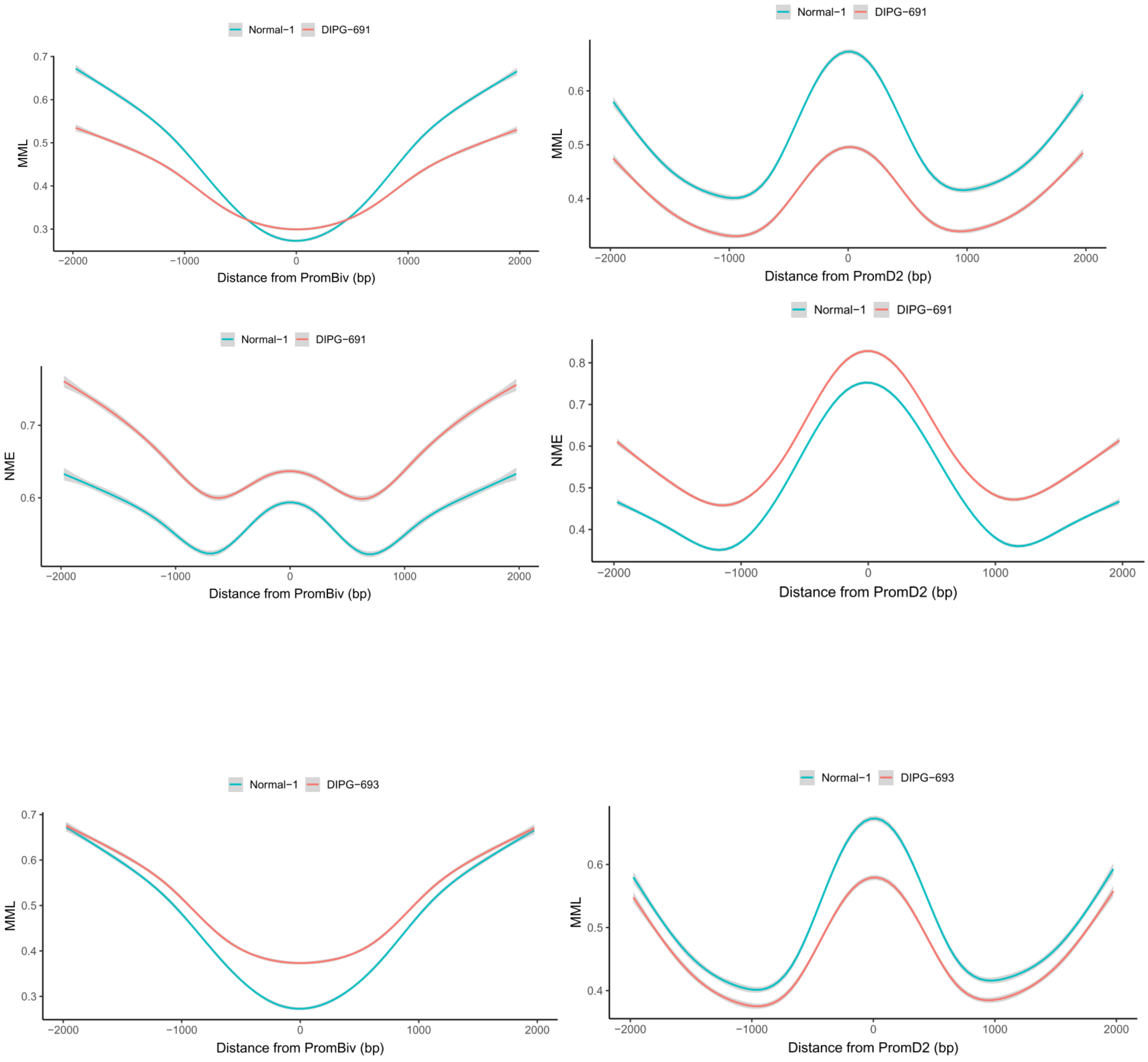

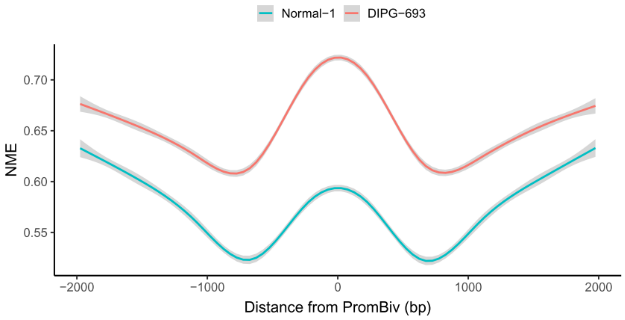
Normal−1
DIPG−693
NME
0.70
0.65
0.60
0.55
−2000
−1000
0
1000
2000
Distance from PromBiv (bp)

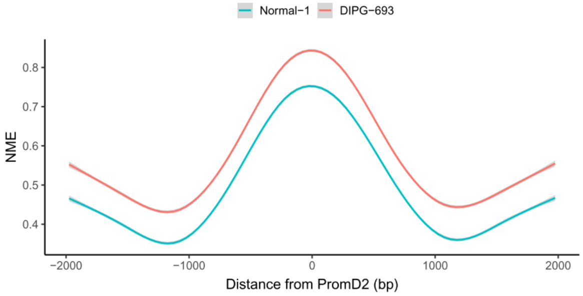
Normal−1
DIPG−693
NME
0.8
0.7
0.6
0.5
0.4
−2000
−1000
0
1000
2000
Distance from PromD2 (bp)

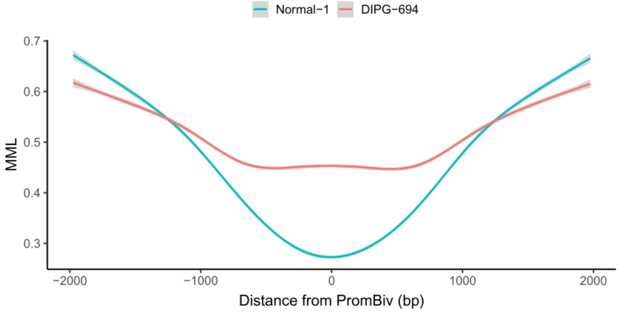
Normal−1
DIPG−694
MML
0.7
0.6
0.5
0.4
0.3
−2000
−1000
0
1000
2000
Distance from PromBiv (bp)

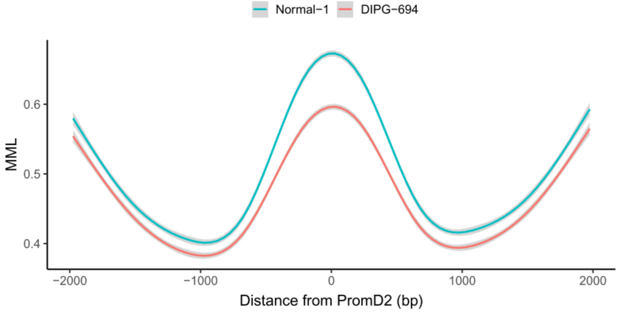
Normal−1
DIPG−694
MML
0.6
0.5
0.4
−2000
−1000
0
1000
2000
Distance from PromD2 (bp)

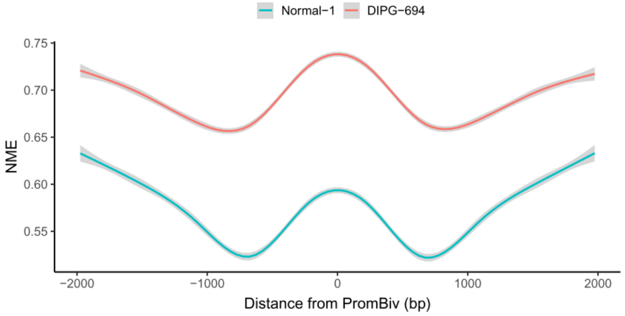
Normal−1
DIPG−694
NME
0.75
0.70
0.65
0.60
0.55
−2000
−1000
0
1000
2000
Distance from PromBiv (bp)

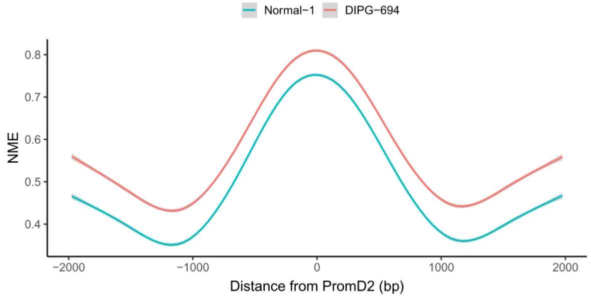
Normal−1
DIPG−694
NME
0.8
0.7
0.6
0.5
0.4
−2000
−1000
0
1000
2000
Distance from PromD2 (bp)

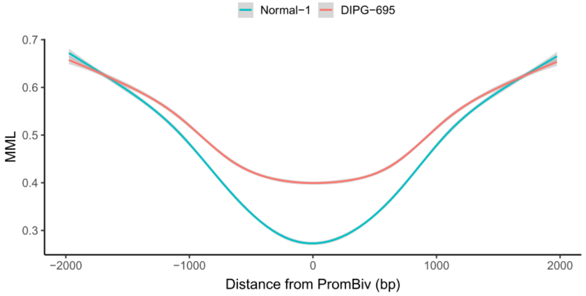
Normal−1
DIPG−695
MML
0.7
0.6
0.5
0.4
0.3
−2000
−1000
0
1000
2000
Distance from PromBiv (bp)

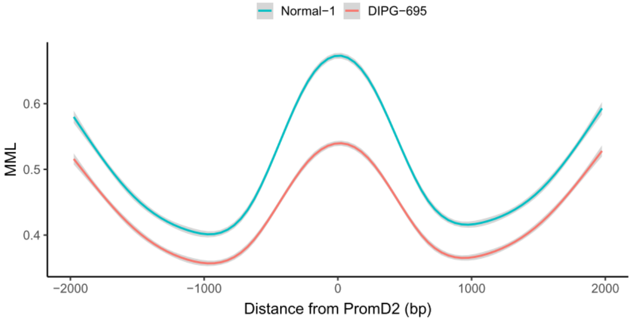
Normal−1
DIPG−695
MML
0.6
0.5
0.4
−2000
−1000
0
1000
2000
Distance from PromD2 (bp)

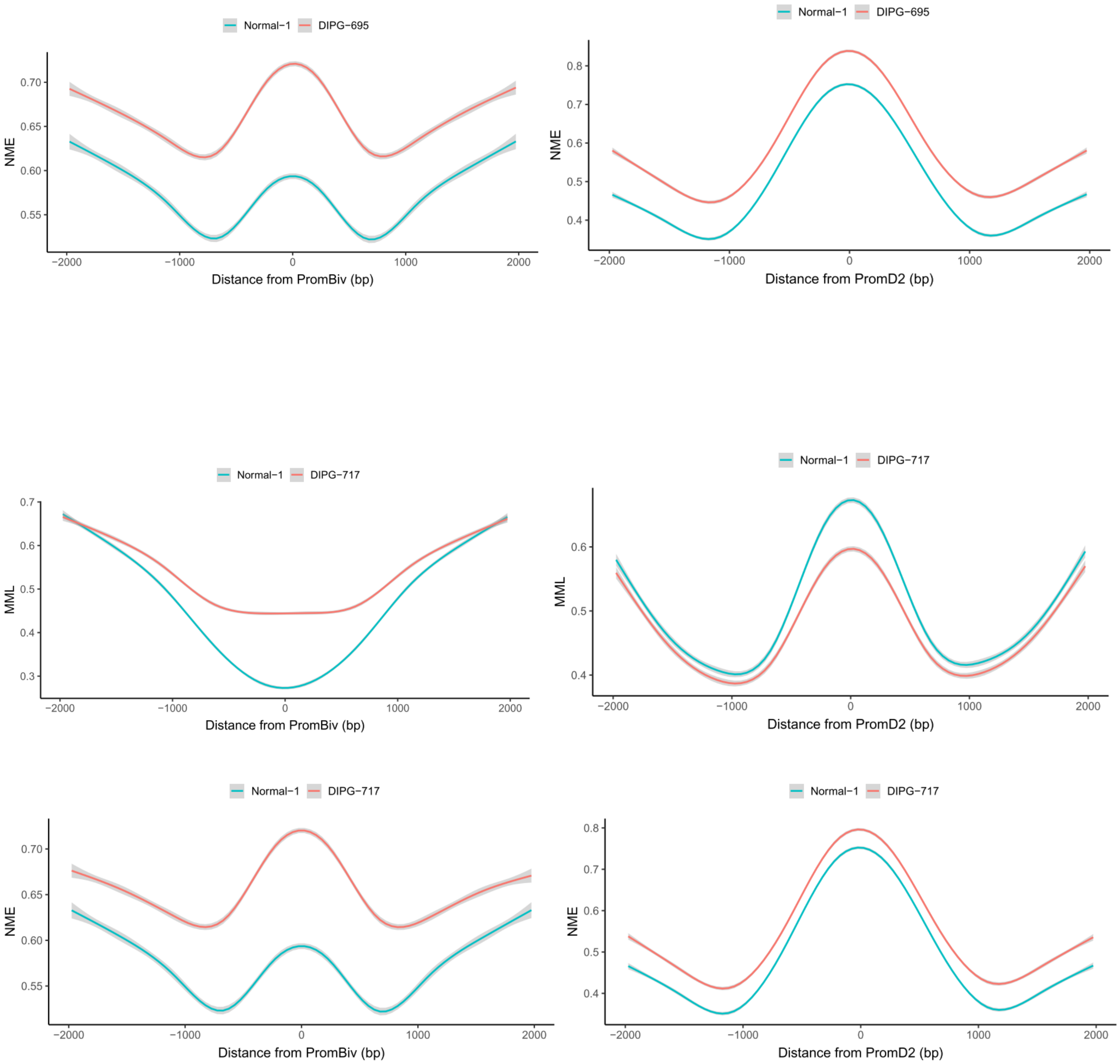
Normal−1
DIPG−695
NME
0.55
0.60
0.65
0.70
−2000
−1000
0
1000
2000
Distance from PromBiv (bp)
Normal−1
DIPG−695
NME
0.4
0.5
0.6
0.7
0.8
−2000
−1000
0
1000
2000
Distance from PromD2 (bp)
Normal−1
DIPG−717
MML
0.3
0.4
0.5
0.6
0.7
−2000
−1000
0
1000
2000
Distance from PromBiv (bp)
Normal−1
DIPG−717
MML
0.4
0.5
0.6
−2000
−1000
0
1000
2000
Distance from PromD2 (bp)
Normal−1
DIPG−717
NME
0.55
0.60
0.65
0.70
−2000
−1000
0
1000
2000
Distance from PromBiv (bp)
Normal−1
DIPG−717
NME
0.4
0.5
0.6
0.7
0.8
−2000
−1000
0
1000
2000
Distance from PromD2 (bp)

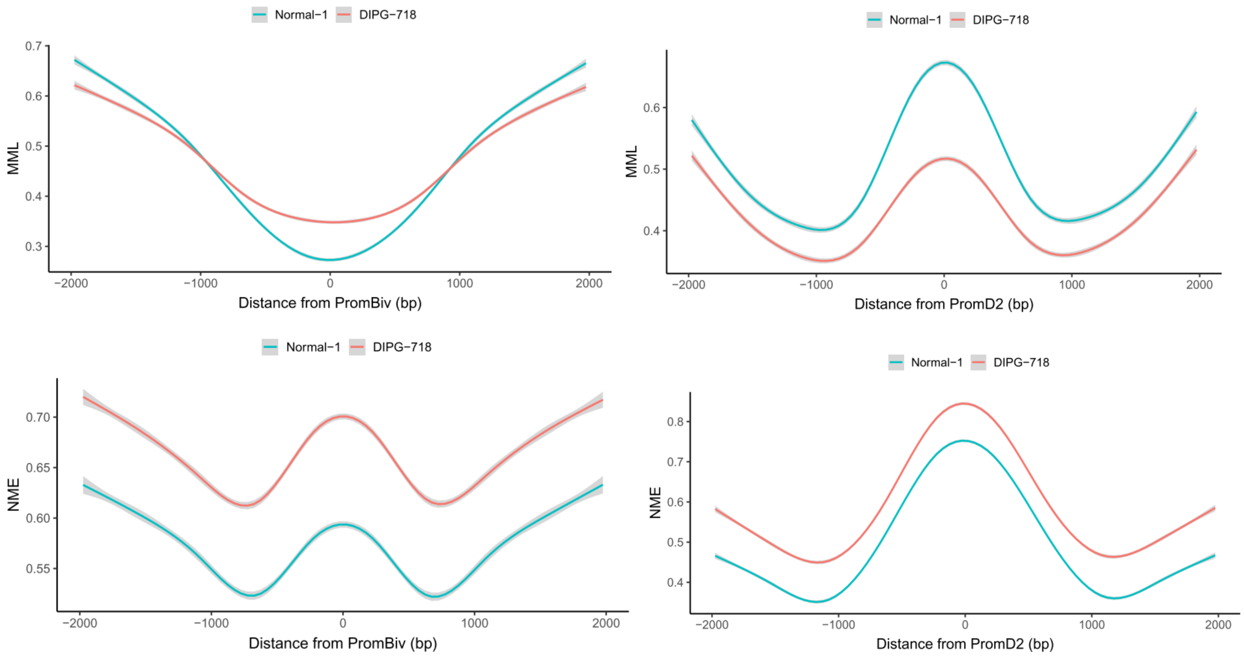
Normal−1
DIPG−718
MML
NME
Distance from PromBiv (bp)
Distance from PromD2 (bp)
−2000
−1000
0
1000
2000

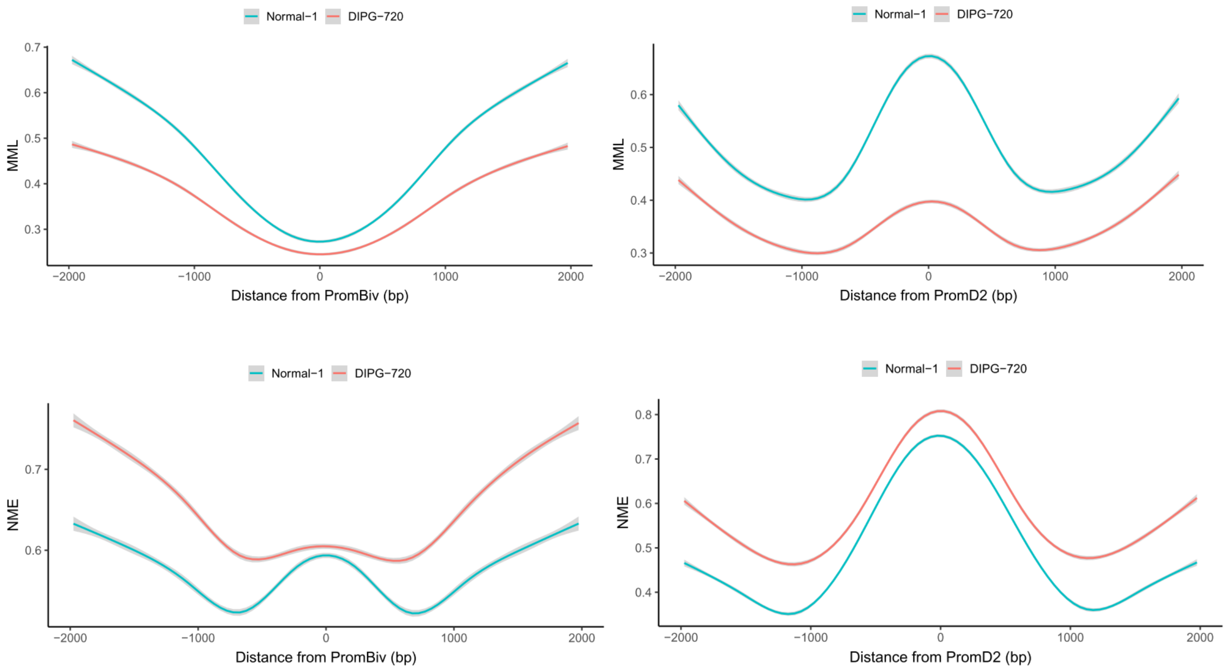
Normal−1
DIPG−720
MML
NME
Distance from PromBiv (bp)
Distance from PromD2 (bp)
−2000
−1000
0
1000
2000

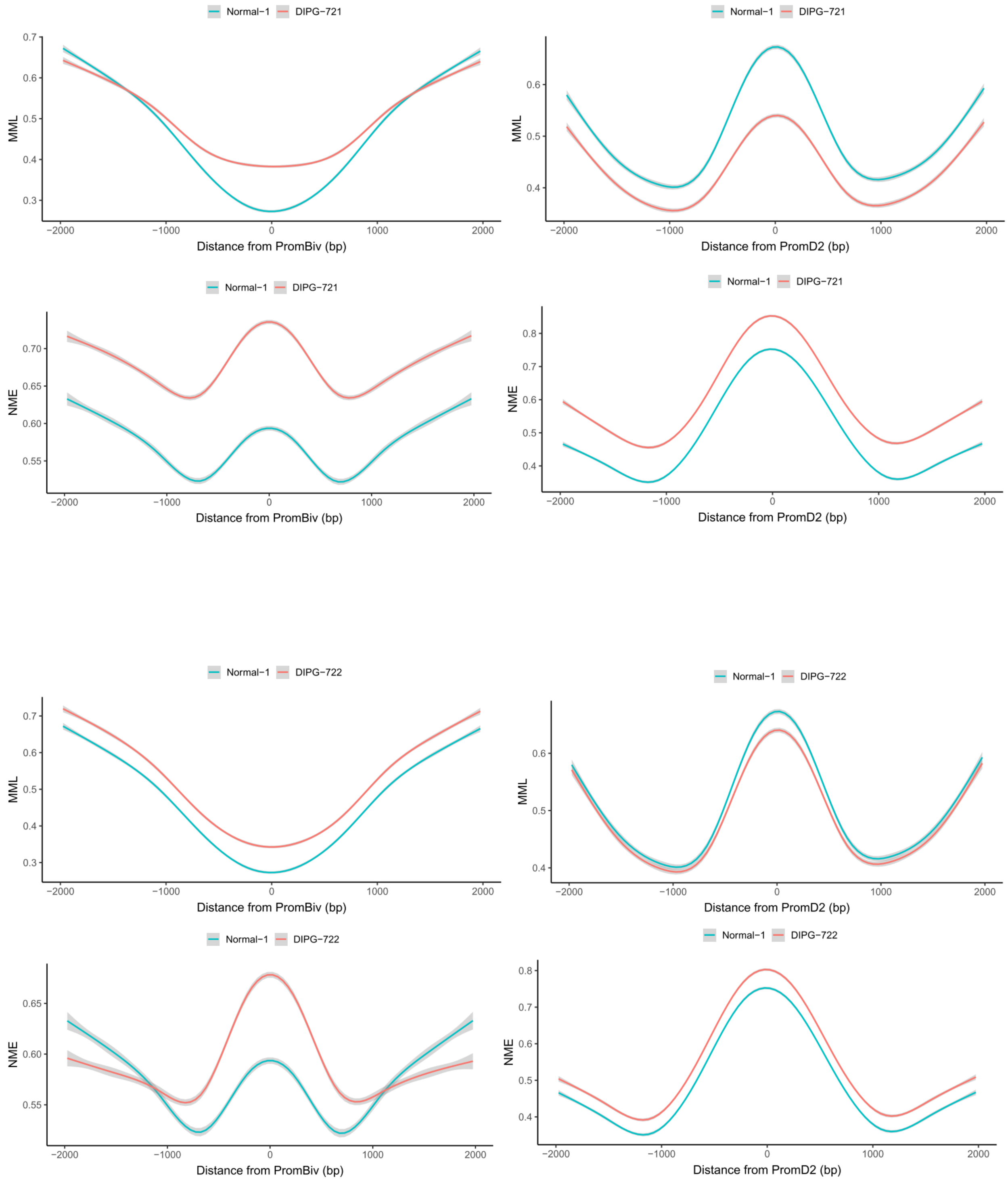

Normal−1
DIPG−721
DIPG−722
MML
NME
Distance from PromBiv (bp)
Distance from PromD2 (bp)
−2000
−1000
0
1000
2000

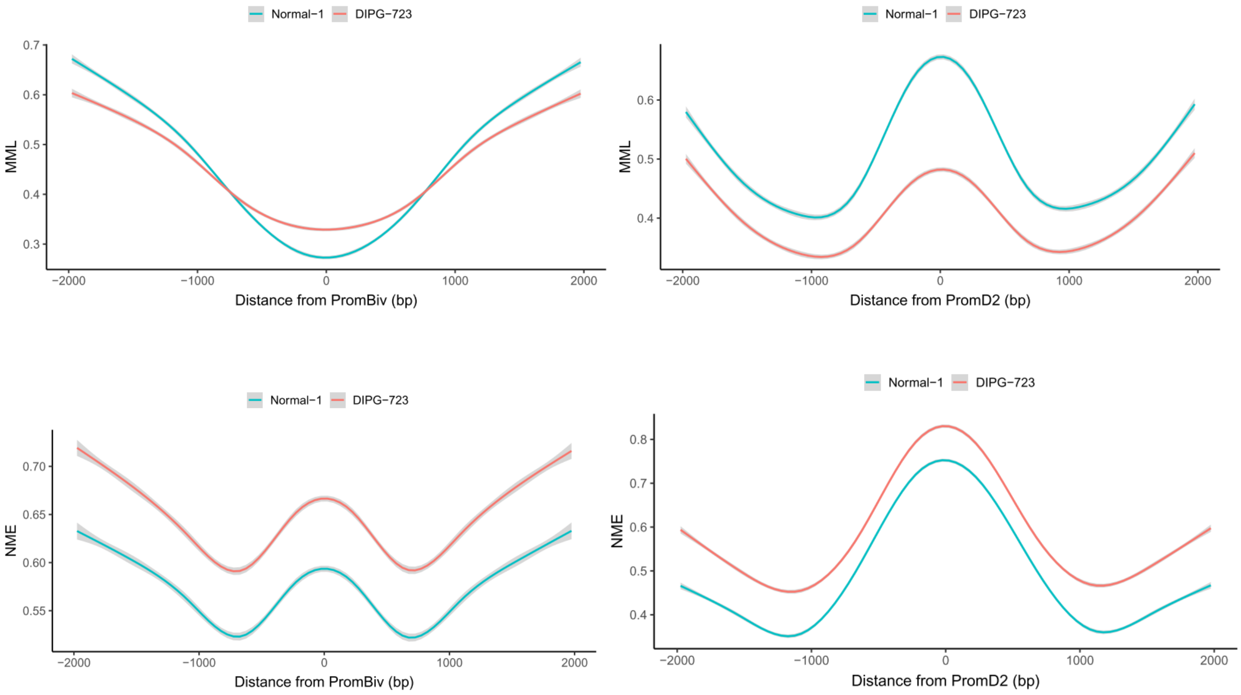

Normal−1
DIPG−723
MML
0.7
0.6
0.5
0.4
0.3
−2000
−1000
0
1000
2000
Distance from PromBiv (bp)
Distance from PromD2 (bp)
NME
0.70
0.65
0.60
0.55
0.8

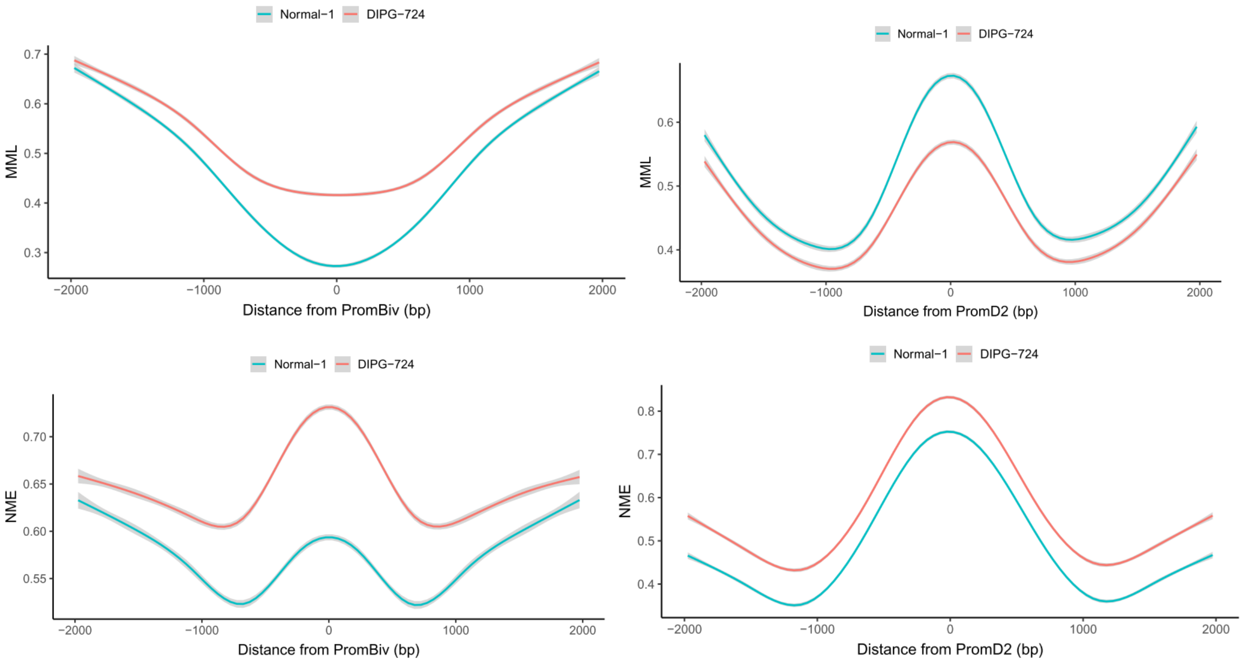

Normal−1
DIPG−724
MML
0.7
0.6
0.5
0.4
0.3
−2000
−1000
0
1000
2000
Distance from PromBiv (bp)
Distance from PromD2 (bp)
NME
0.70
0.65
0.60
0.55
0.8

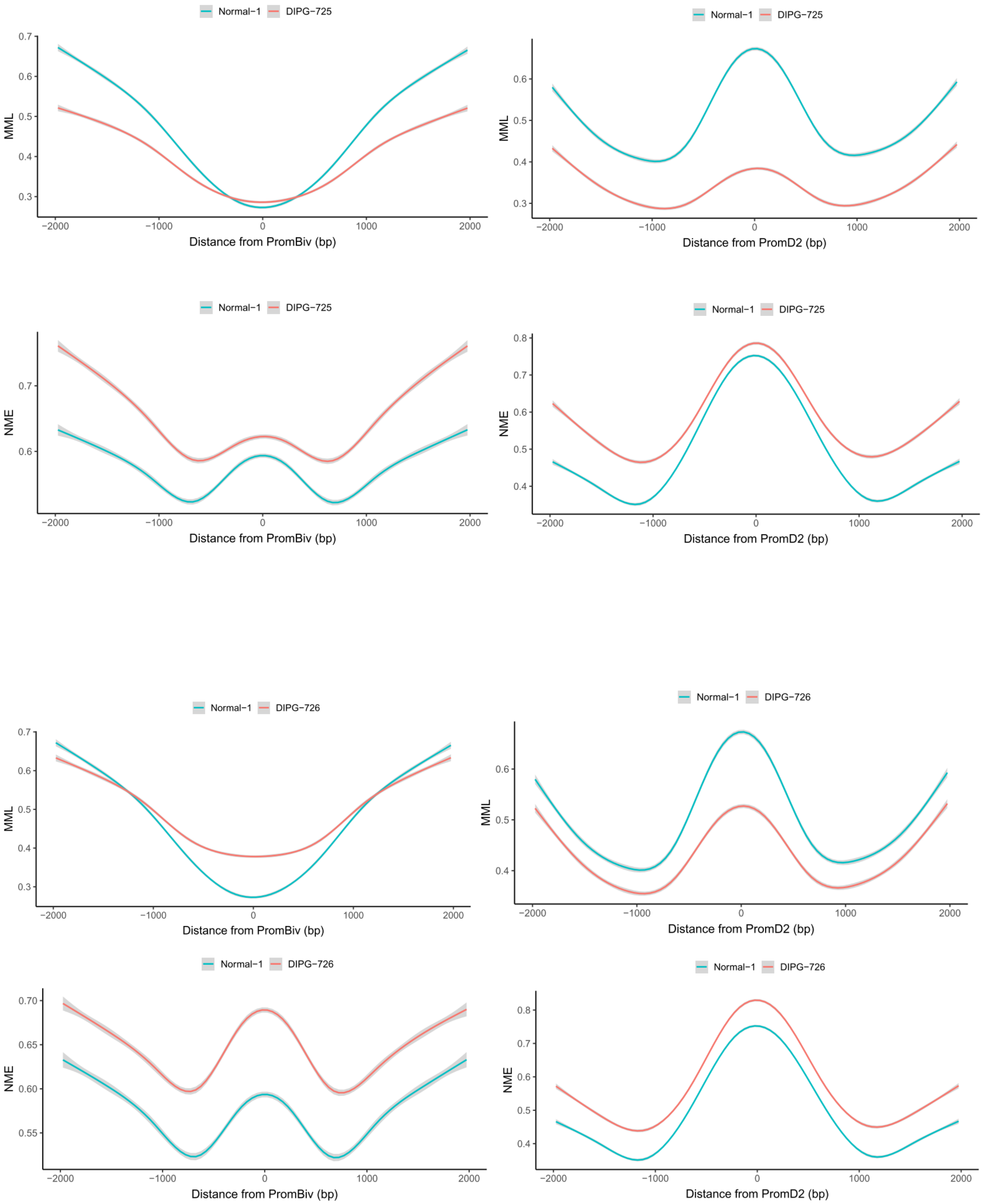

Normal−1
DIPG−725
MML
Distance from PromBiv (bp)
Distance from PromD2 (bp)
NME
Normal−1
DIPG−726
MML
Distance from PromBiv (bp)
Distance from PromD2 (bp)
NME
−2000
−1000
0
1000
2000

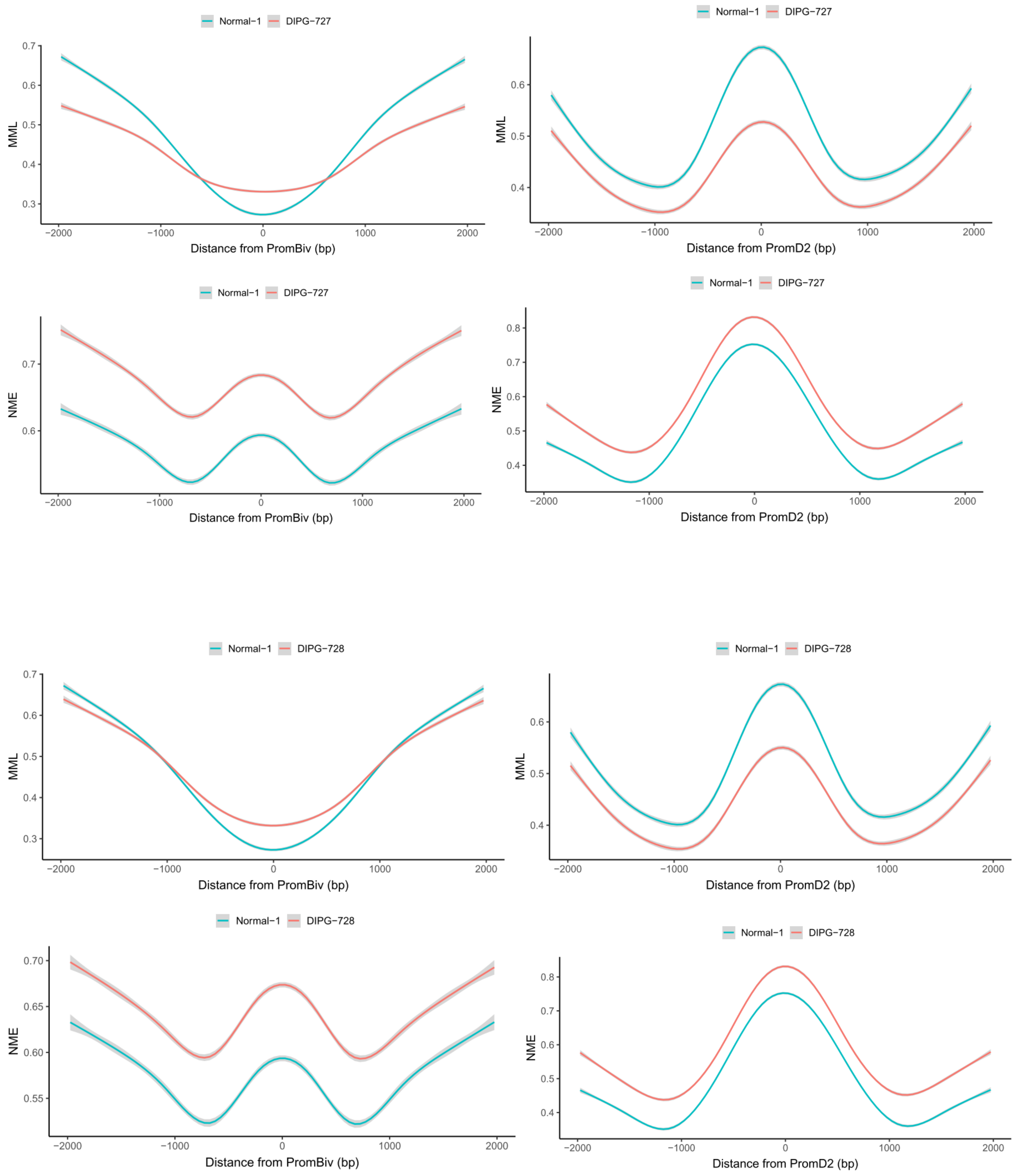

Normal−1
DIPG−727
MML
Distance from PromBiv (bp)
Normal−1
DIPG−727
MML
Distance from PromD2 (bp)
Normal−1
DIPG−727
NME
Distance from PromBiv (bp)
Normal−1
DIPG−727
NME
Distance from PromD2 (bp)
Normal−1
DIPG−728
MML
Distance from PromBiv (bp)
Normal−1
DIPG−728
MML
Distance from PromD2 (bp)
Normal−1
DIPG−728
NME
Distance from PromBiv (bp)
Normal−1
DIPG−728
NME
Distance from PromD2 (bp)

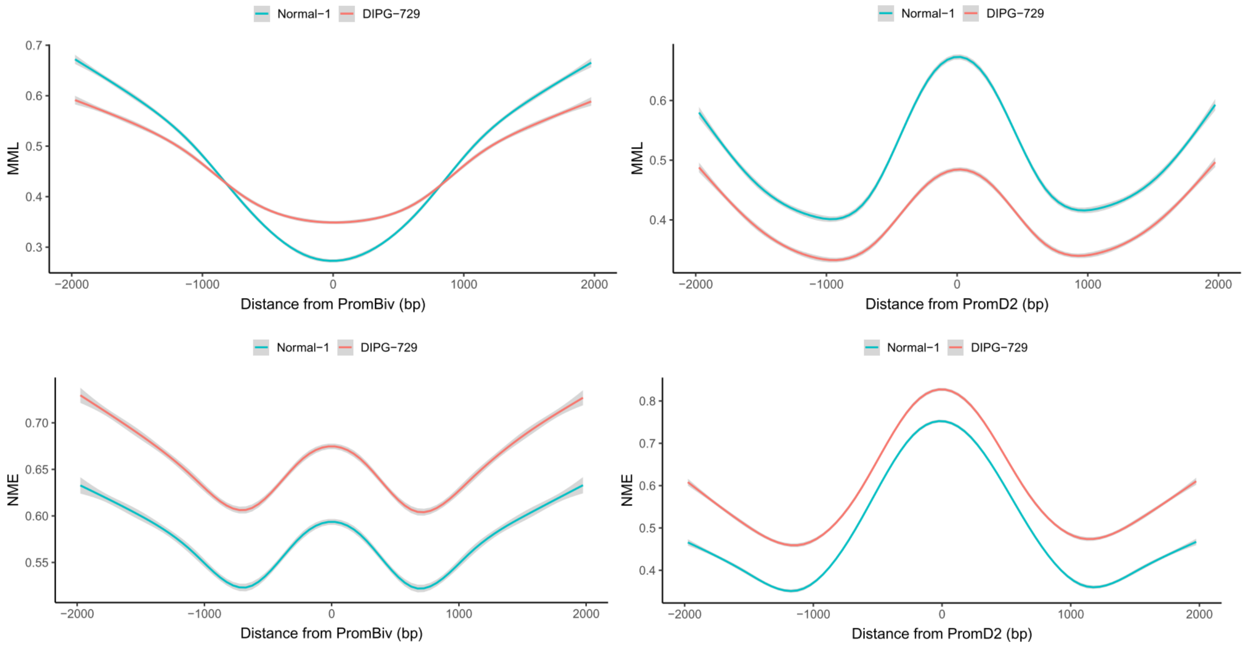

Normal−1
DIPG−729
MML
0.7
0.6
0.5
0.4
0.3
−2000
−1000
0
1000
2000
Distance from PromBiv (bp)
Normal−1
DIPG−729
MML
0.6
0.5
0.4
Distance from PromD2 (bp)
Normal−1
DIPG−729
NME
0.70
0.65
0.60
0.55
Distance from PromBiv (bp)
Normal−1
DIPG−729
NME
0.8
0.7
0.6
0.5
0.4
Distance from PromD2 (bp)

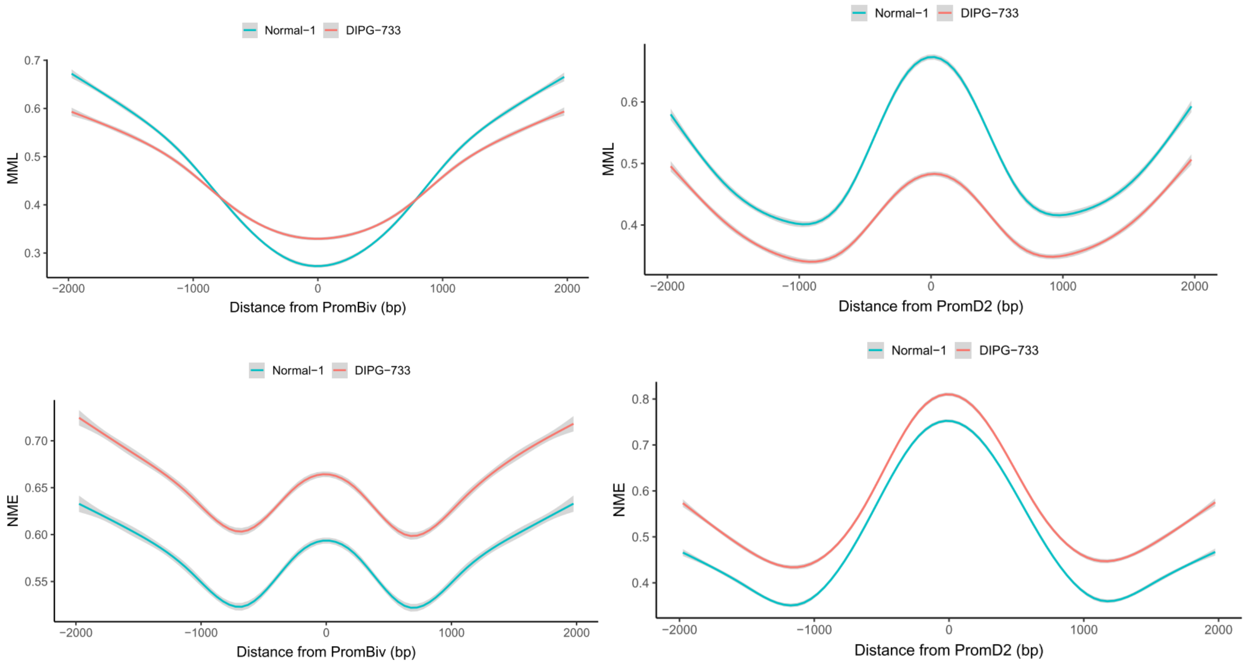

Normal−1
DIPG−733
MML
0.7
0.6
0.5
0.4
0.3
−2000
−1000
0
1000
2000
Distance from PromBiv (bp)
Normal−1
DIPG−733
MML
0.6
0.5
0.4
Distance from PromD2 (bp)
Normal−1
DIPG−733
NME
0.70
0.65
0.60
0.55
Distance from PromBiv (bp)
Normal−1
DIPG−733
NME
0.8
0.7
0.6
0.5
0.4
Distance from PromD2 (bp)

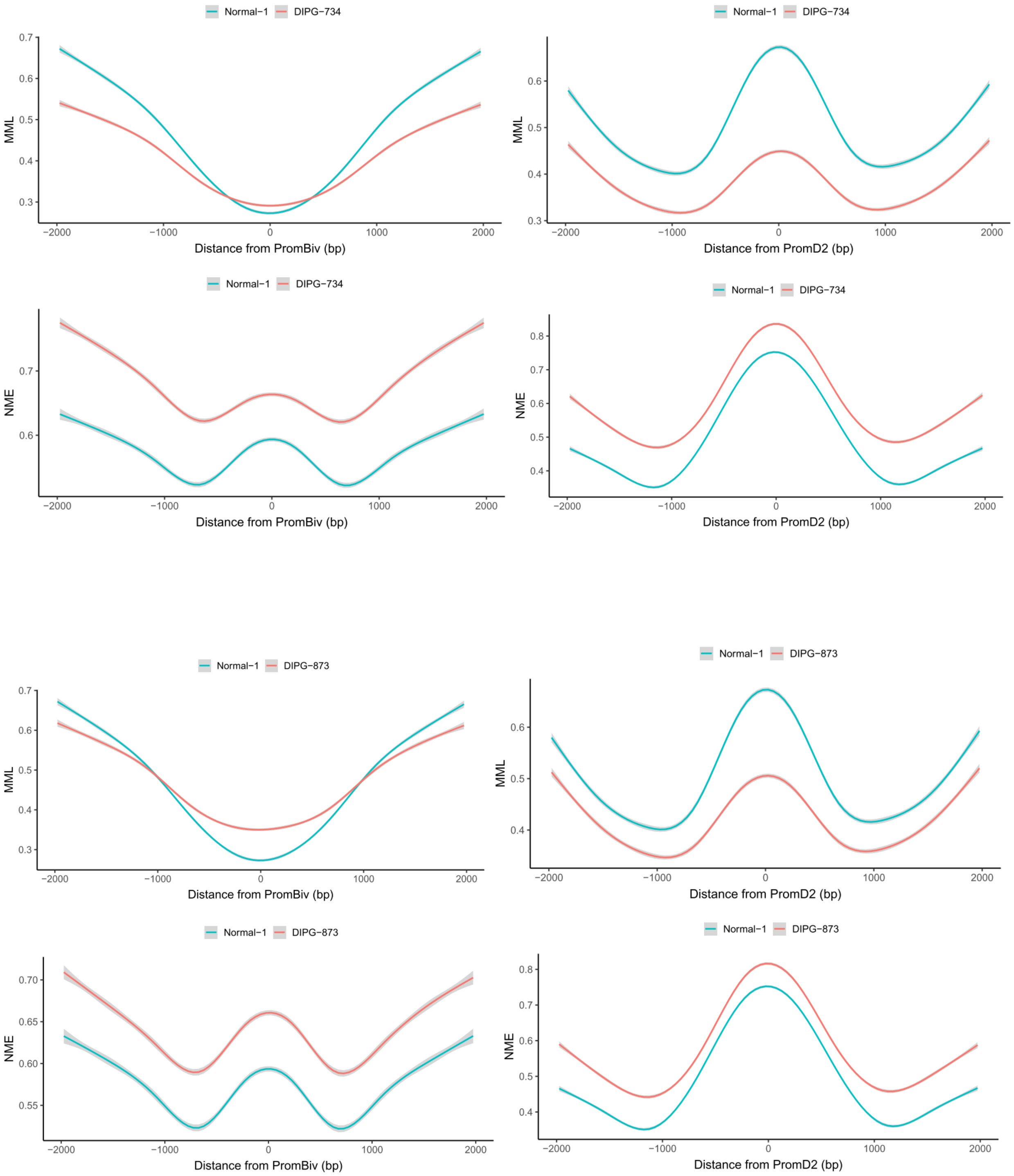

Normal−1
DIPG−734
MML
NME
Distance from PromBiv (bp)
Distance from PromD2 (bp)
Normal−1
DIPG−873
−2000
−1000
0
1000
2000
0.3
0.4
0.5
0.6
0.7
0.8
0.55
0.60
0.65
0.70

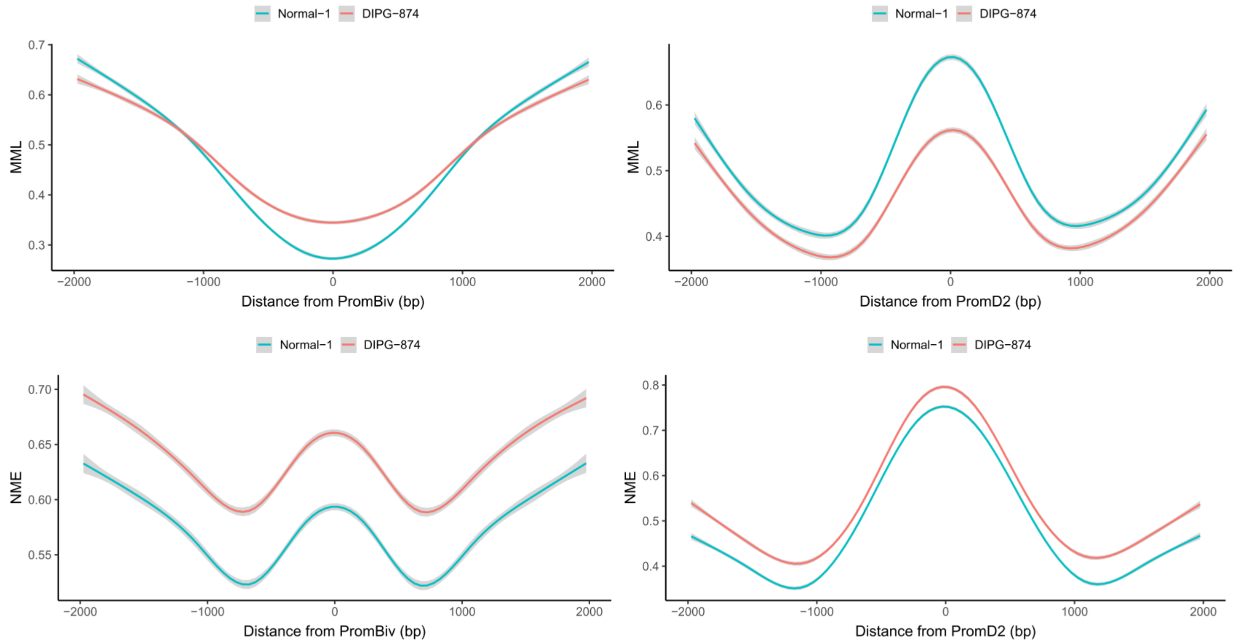
Normal−1
DIPG−874
MML
Distance from PromBiv (bp)
MML
Distance from PromD2 (bp)
NME
Distance from PromBiv (bp)
NME
Distance from PromD2 (bp)

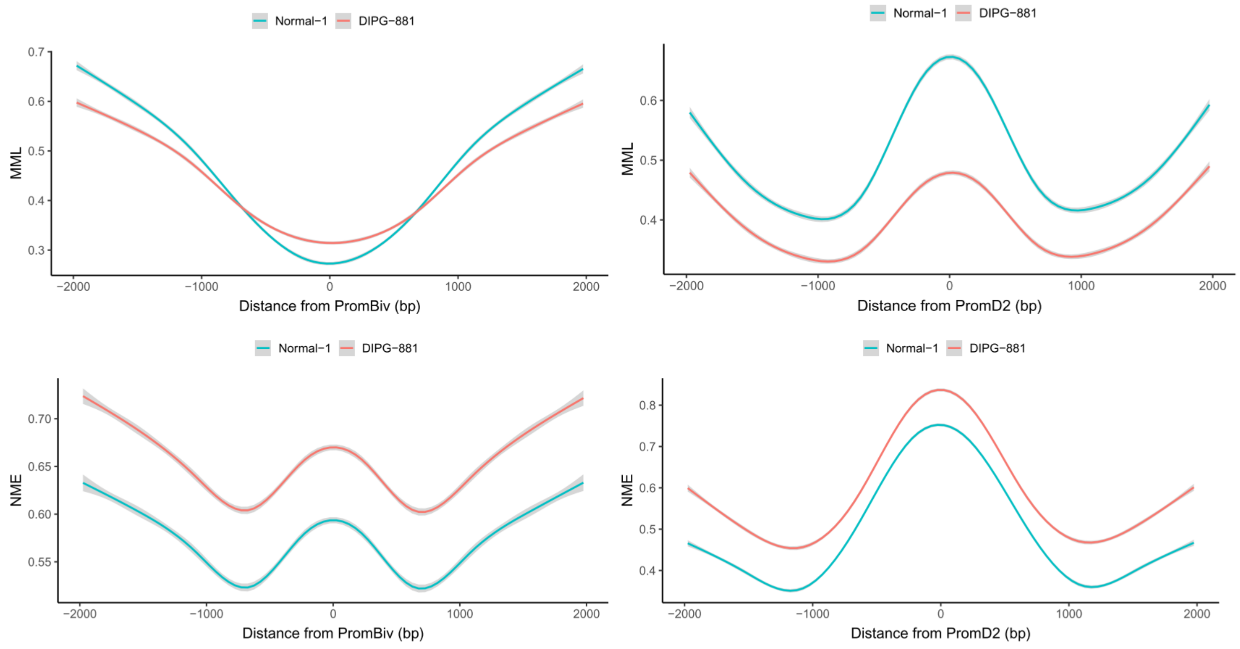
Normal−1
DIPG−881
MML
Distance from PromBiv (bp)
MML
Distance from PromD2 (bp)
NME
Distance from PromBiv (bp)
NME
Distance from PromD2 (bp)

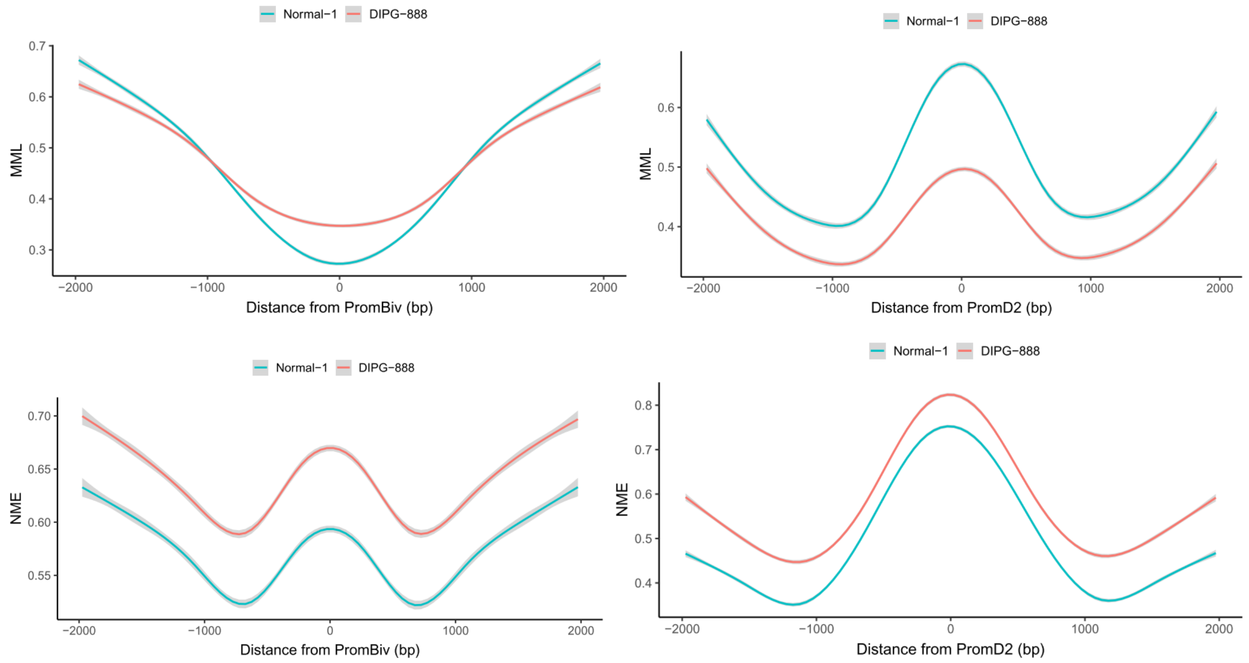

Normal−1
DIPG−888
MML
Distance from PromBiv (bp)
Distance from PromD2 (bp)
NME
Distance from PromBiv (bp)
Distance from PromD2 (bp)
−2000
−1000
0
1000
2000

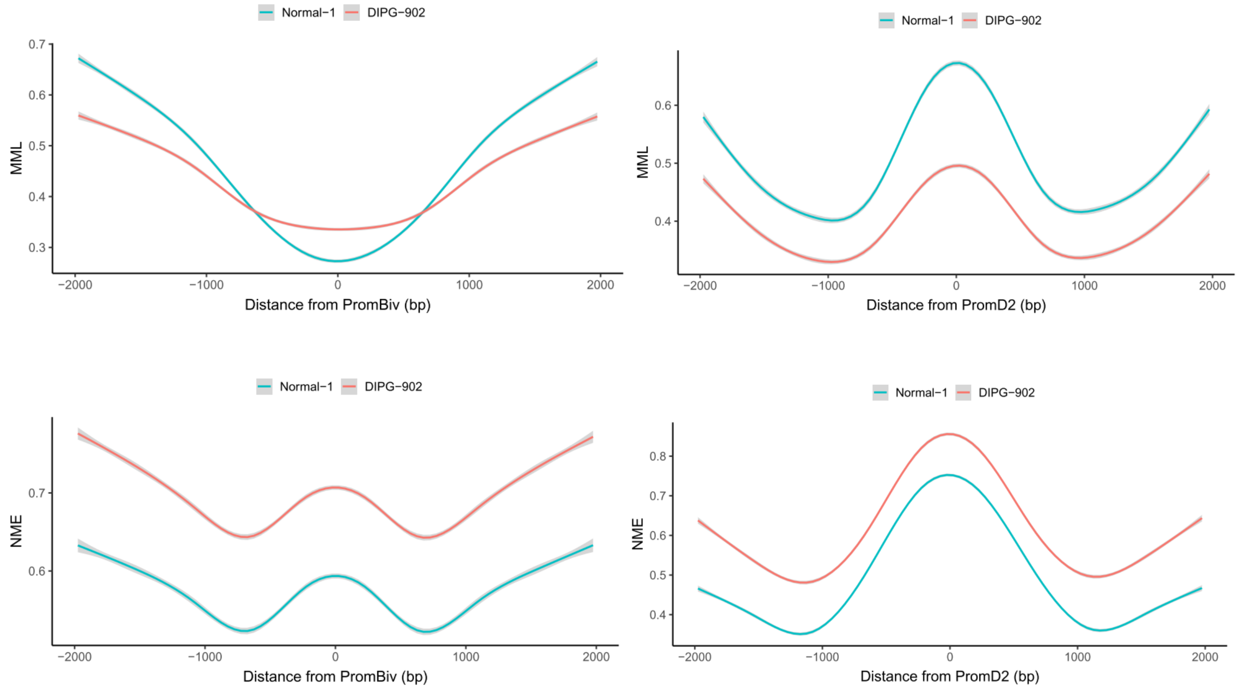

Normal−1
DIPG−902
MML
Distance from PromBiv (bp)
Distance from PromD2 (bp)
NME
Distance from PromBiv (bp)
Distance from PromD2 (bp)
−2000
−1000
0
1000
2000
